# *Metarhizium caribense* sp. nov., a Novel Species of Entomopathogenic *Metarhizium* Fungi Associated with Weevils Impairing Coffee, Sugar Cane and Sweet Potato Cultivation

**DOI:** 10.3390/jof10090612

**Published:** 2024-08-28

**Authors:** Yamilé Baró Robaina, Christina Schuster, Rafael F. Castañeda-Ruiz, Yohana Gato Cárdenas, María Elena Márquez Gutiérrez, Amaia Ponce de la Cal, Andreas Leclerque

**Affiliations:** 1 Plant Health Research Institute (INISAV), 110 Str. 514, Havana 11600, Cuba; 2 Department of Biology, Technische Universität Darmstadt (TUDa), Schnittspahnstraße 10, 64287 Darmstadt, Germany

**Keywords:** *Metarhizium anisopliae* species complex, *Metarhizum pinghaense*, ribosomal intergenic spacer (rIGS), species-discriminating diagnostic PCR, self-splicing group-I intron, MzIGS3 (DUF895), 5TEF, sweet potato weevil *Cylas formicarius*, coffee berry borer *Hypothenemus hampei*, sugarcane rootstalk borer *Diaprepes abbreviate*

## Abstract

(1) Background: Insect pathogenic fungi of the genus *Metarhizium* are under study and in application as highly solicited, more eco-system friendly substitutes for chemical insecticides in many countries and in different agricultural contexts. In Cuba and Florida, *Metarhizium* strains have previously been isolated from economically important coffee and sugar cane pests. (2) Methods: Unambiguous species delineation within the *Metarhizium anisopliae* species complex is methodologically challenging. Recently, a species-discriminating PCR approach has been developed based on ribosomal intergenic spacer (rIGS) sequences that covered the prominent four “PARB” species within the complex. This approach is combined here with further genetic markers and is extended to a further species. (3) Results: *Metarhizium* isolates from Cuba, found to be more naturally associated with the coffee berry borer, *Hypothenemus hampei*, were morphologically, microscopically and molecular taxonomically characterized. Multilocus sequence analysis based on 5TEF, MzIGS3 and rIGS markers delineated these weevil-associated strains from all previously established *Metarhizium* species. (4) Conclusions: The isolates under study represent a new fungal taxon proposed to be designated *Metarhizium caribense*. The rIGS-based species-discriminating diagnostic PCR is a suitable tool for the identification of new *Metarhizium* species and can be productively combined to approaches using further genetic markers.

## 1. Introduction

The taxonomic genus *Metarhizium* Sorokin (Hypocreales; Clavicipitaceae) comprises fungal pathogens of a wide range of insect hosts and is globally one of the most widely studied fungi for biological control. *Metarhizium* fungi are widespread in nature and may fulfill diverse functions in ecosystems [1]. Their efficacy and biocontrol potential has been successfully exploited to develop microbial insecticides [2].

Traditionally, species identification within the genus *Metarhizium* has been based on phenotypic characters such as the morphology and dimensions of conidia, conidiophores and phialides. However, these morphological criteria alone are not sufficient to identify fungal isolates at the species level. Several studies [3,4,5] have underlined the importance of molecular taxonomy to consistently differentiate between *Metarhizium* species. During the past two decades, a multilocus sequence analysis (MLSA) scheme, formed as taxonomic markers of partial sequences of the genes encoding translation elongation factor 1 alpha (EF1A) and the RNA polymerase II subunits 1 and 2 (RPB1 and RPB2, respectively), together with the intron-rich 5′-region of the translation elongation factor 1 alpha gene, referred to as 5TEF, have gained particular relevance in *Metarhizium* systematics [6,7]. As a result of systematic studies employing these genetic markers, the boundaries of the genus *Metarhizium* were redefined [4,8] and numerous new *Metarhizium* species were introduced [5,6,9,10,11,12,13]. By now, a total of 111 *Metarhizium* species have been described (Index Fungorum). In the currently accepted internal structure of the genus, two subsets of taxa clustered into species complexes referred to as *Metarhizium anisopliae sensu lato* and *Metarhizium flavoviride sensu lato*. Within the *M. anisopliae* complex, a tight cluster of species informally termed the “PARB clade”, in accordance with the species epithets of the four originally included taxa, namely *M. pinghaense*, *M. anisopliae*, *M. robertsii* and *M. brunneum*, comprises a group of fungi of high relevance for both fundamental research and insect biocontrol [14]. Recently, the species *Metarhizium humberi* has been described as a fifth taxon within the PARB clade [15,16,17].

In order to improve species delineation within these species complexes, seven intergenic regions of nuclear genomes were identified as additional markers across the *M. anisopliae* complex [18]. One of these sequences, termed MzIGS3 or DUF895, has been successfully employed in both species delineation [18,19] and diversity studies [15,20]. Moreover, the intergenic spacer sequence of the ribosomal RNA operon (rIGS) has been evaluated as a molecular taxonomic marker for *Metarhizium* fungi [18,19,21,22]. Recently, an rIGS sequence-based diagnostic PCR protocol for the sequencing and independent delineation of *Metarhizium* PARB clade species has been established [19].

In several genera of entomopathogenic fungi, including *Metarhizium*, 28S rRNA genes have been found to be interrupted by self-splicing group-I introns at four conserved positions [23,24,25]. Sequence variation and polymorphism present at these “hot spots” have been exploited in the molecular identification of these fungi [26,27,28,29], including *Metarhizium* [21,30]

In Cuba, systematic studies in microbial control involving *Metarhizium* strains started about four decades ago, targeting economically important pests, including the sweet potato weevil, *Cylas formicarius* Fabricius (Coleoptera: Brentidae). This insect is one of the main pests in sweet potato cultivation in Cuba and numerous other countries worldwide. Available microbial control methods are not sufficient to satisfy growers’ demand, and a screening program for new fungal isolates that hold potential for the development of innovative bio-insecticides is in continuous implementation. As part of these R&D activities, a set of *Metarhizium* isolates from Cuba had been previously investigated. Five of the isolates were assigned to the species *M. anisopliae sensu stricto* and one to *M. robertsii* [31]. However, for three further isolates that had previously been found pathogenic to adults of *C. formicarius* [32], conclusive species-level assignment appeared difficult.

The purpose of the present study is to conclusively identify these potential biocontrol strains at the species-level. Consequently, our investigations have led us to propose the introduction of a new species within the genus *Metarhizium*.

## 2. Materials and Methods

### 2.1. Fungal Strains and Isolates

Three fungal isolates from the INISAV culture collection of micro-organisms were used in this study, which originate from insect and soil samples from different environments in Cuba (Table 1). Isolates were recovered from sterile mineral oil preservation and grown on Sabouraud dextrose agar (SDA) at 26 °C for approximately 15 days. Single-spore cultures were prepared using the method described by Inglis et al. [33]. Reference strains for taxonomic studies were provided by the ARS Collection of Entomopathogenic Fungal Cultures (ARSEF) in Beltsville, Maryland, U.S.A., and by the Quesada-Moraga laboratory at University of Cordoba, Spain (Supplementary Table S1).

### 2.2. Morphological and Cultural Characterization of Fungal Isolates

For morphological and cultural characterization, a conidial suspension at 10^7^ conidia per ml was prepared for each fungal isolate. Fungal suspension (0.1 mL) was spread on a Petri dish with SDA. Petri dishes were incubated in the dark at 26 °C for 72 h. A mycelium disc of 5 mm diameter from 3-day-old cultures was transferred to the center of a 90 mm Petri dish containing potato dextrose agar (PDA), Sabouraud dextrose agar (SDA) or complete medium (MC: 0.4 g/L KH_2_PO_4_, 1.4 g/L Na_2_HPO_4_, 0.6 g/L MgSO_4_, 1.0 g/L KCl, 0.7 g/L NH_4_NO_3_, 10 g/L glucose, 5 g/L yeast extract, 15 g/L agar). Five replicates were prepared for each isolate and incubated under the conditions described above. The growth of the isolates was observed from 72 h to 14 days post-inoculation. Cultural characteristics of fungal growth as colony diameter, color, border and texture were determined. 

For microscopic characterization, fungi were grown in a synthetic low-nutrient agar (SNA) culture medium (1 g/L K_2_HPO_4_, 1 g/L KNO_3_, 0.5 g/L MgSO_4_∙7H_2_O, 0.5 g/L KCl, 0.2 g/L glucose, 0.2 g/L sucrose, 20 g/L agar) at 26 ± 1 °C in darkness for 5 days. Fungal mycelium was mounted in a glass slide with 90% lactate acid solution and 0.01% lactophenol cotton blue and observed with an optical phase–contrast microscope model Nikon Eclipse 80i (Nikon, Tokyo, Japan) using a 40× magnifying microscope objective. The conidial morphology was described, and the conidial dimensions (length × width) were determined. Fungal structures were photographed with a Nikon DS-Fi1 Camera (Nikon, Tokyo, Japan).

*Metarhizium* fungi were morphologically identified according to the taxonomic descriptions by Driver et al. [3,6].

### 2.3. DNA Extraction

For DNA extraction, fungal isolates were grown at 25 °C on YPG agar (2 g/L yeast extract, 10 g/L peptone, 20 g/L glucose) containing 25 µg/mL of tetracycline for 1–2 weeks. Approximately 100 mg of mycelium were transferred to a screw-capped 2 ml microcentrifuge tube containing Lysing Matrix C (MP Biomedicals, Santa Ana, CA, USA). Samples were frozen in liquid nitrogen and processed for 30–60 s at intermediate speed in a Minilys homogenizer (Bertin technologies, Montigny-le-Bretonneux, France). DNA was extracted from homogenized samples using the DNeasy Plant kit (Qiagen, Venlo, The Netherlands) according to the standard protocol as provided by the manufacturer. Purified DNA was eluted in 100 µL EB buffer (10 mM Tris-Cl, pH = 8.5) and stored at −20 °C. DNA concentrations were determined using a NanoDrop One device (Thermo Scientific, Waltham, MA, USA).

### 2.4. Preparative PCR Amplification and Marker Sequence Determination

All PCR reactions performed in this study were run in a T-One thermocycler (Biometra, Göttingen, Germany) using 0.025 U/µL GoTaq polymerase (Promega, Fitchburg, MA, USA) with dNTP and oligonucleotide primer concentrations of 200 µM and 500 nM, respectively. Preparative PCR for DNA sequence determination was performed as 50 µL reactions using a generalized PCR protocol consisting of one initial denaturation step of 95 °C for 2 min, 35 cycles of 30 sec at 95 °C, 30 s at the primer-specific annealing temperature and a 72 °C elongation step of amplicon-specific time, followed by a 5 min final elongation step at 72 °C. Using this protocol, the following marker sequences were amplified from extracted fungal DNA: EF1A (using primer pair EF1A-983F/EF1A-2218R), RPB1 (RPB1Af/RPB1Cr), RPB2 (RPB2-5f/RPB2-7r), 5TEF (EF1T/EF2T), MzIGS3 (MzIGS3-1F/MzIGS3-4R), rIGS-ID800 (Migs1-F1/Migs850-R1), the complete rIGS sequence (Migs1-F2/Migs1200R and Migs1100F/Migs2000R), and the group-I intron insertion region of the 28S rRNA gene (28Si-I29F/28Si-E24R). Primer sequences as well as reaction-specific parameters are indicated in Supplementary Table S2. Formation of a single PCR product of expected apparent size was routinely controlled through horizontal electrophoresis in 1× TAE buffer (40 mM TRIS, 20 mM acetic acid, 1 mM EDTA, pH 8.3) of a 5 µL sample using 1% agarose gels stained with 5 µL/100 mL Roti Gelstain (Carl Roth, Karlsruhe, Germany). PCR products were purified using the Qiaquick PCR purification kit (Qiagen, Venlo, The Netherlands) according to the standard protocol provided by the manufacturer and comprising the final elution in 50 µL of EB buffer (10 mM Tris·Cl, pH 8.5). 

Sanger sequencing of PCR products was performed externally (by Starseq, Mainz, Germany or MicroSynth, Göttingen, Germany) using respective PCR primers and additional sequencing primers as indicated in Supplementary Table S2. Raw sequence data were combined into a single consensus sequence for each fungal specimen and marker using version 11 of the MEGA software package [34]. Sequences determined were submitted to the GenBank database under the accession numbers indicated in Supplementary Table S1. Homologous GenBank database entries were searched for using the BlastN software tool, version 2.15.0 [35,36].

### 2.5. Phylogenetic Reconstruction

Nucleotide sequences were aligned using the CLUSTAL W function [37] as implemented in version 11 of the MEGA software package [34]; for comprehensive analyses, concatenations of marker sequences were aligned in a marker-by-marker approach. The Tree-Puzzle version 5.2 software [38] was used to estimate data-set-specific parameters, such as nucleotide frequencies, the percentage of invariable sites, the transition/transversion ratio and the alpha-parameter for the gamma-distribution-based correction of rate heterogeneity among sites. Two algorithms were used to reconstruct *Metarhizium* phylogenies: (i) a p-distance matrix-based neighbor joining (NJ) method, as implemented in MEGA 11, and (ii) the maximum likelihood (ML) method, as implemented in version 3.1 of the PhyML software tool [39], using the Hasegawa–Kishino–Yano model of nucleotide substitution [40] under the assumption of a gamma-distribution-based model of rate heterogeneity [41], allowing for eight rate categories. For both reconstruction methods, alignment gaps and missing data were treated using a pairwise deletion setting, and tree topology confidence limits were explored in non-parametric bootstrap analyses over 1000 pseudo-replicates.

### 2.6. Diagnostic PCR Amplification for Species Discrimination

Prior to use as diagnostic PCR templates, extracted DNA samples were diluted in sterile 1× TE buffer (10 mM TRIS, 1 mM EDTA, pH 8.0) to a concentration of 3 ng/µL. Diagnostic PCRs were typically performed as 20 µL reactions containing 100 pg/µL of extracted genomic DNA template. The diagnostic PCR protocol established for rIGS-based species-discrimination consisted of one initial denaturation step of 95 °C for 2 min, 35 cycles of denaturation at 95 °C for 30 s, annealing at 60 °C for 30 s and elongation at 72 °C for 45 s, followed by a 2 min final elongation step at 72 °C. Diagnostic PCR primer pairs employed are listed in Supplementary Table S3. The rationale for the design of a new diagnostic primer pair specific for *M. caribense* was the same as that detailed in [19]. For identification of long 28Si4 intron variants, discriminating forward primers were combined to the same reverse primer, 28Si-E24Rshort (Supplementary Table S3). Diagnostic PCR results were controlled through horizontal gel electrophoresis typically using 10 µL samples on 1.5% agarose gels.

## 3. Results

### 3.1. Molecular Taxonomic Identification Based on Protein-Encoding Gene Sequences

For the three Cuban isolates investigated, consistent consensus sequences were obtained for the molecular taxonomic markers used. Comparisons with reference sequences gave rise to alignments comprising lengths of 629 bp (5TEF), 882 bp (EF1A), 552 bp (RPB1), and 1026 bp (RPB2), respectively. Pairwise sequence similarities between the marker sequences from strains LBM-30, LBM-41 and LBM-42 were 100% for both 5TEF and EF1A and 99.8–100% for both RPB1 and RPB2.

When used as a query in unfiltered BlastN searches across the GenBank database, the 5TEF sequence from isolate LBM-41 identified as best hit a single identical entry (i.e., displaying 100% similarity at 100% sequence coverage) assigned to the *M. pinghaense* strain ARSEF 5197. Moreover, eight second-best hits at >99.8% similarity (100% coverage) were identified, followed by a plethora of entries at <99.7% similarity. The group of second-best hits comprised 5TEF sequences assigned to the species *M. pinghaense*, including strains ARSEF 1448, ARSEF 2162, ARSEF 3180 and ARSEF 4290. GenBank searches using the EF1A, RPB1 and RPB2 marker sequences as query did not give rise to differentiated outcomes defining a best hit or a group of best hits. 

In the phylogenetic tree reconstructed from an alignment of the 5TEF sequences from the isolates under study with the identified best and second-best hit entries and 5TEF sequences from reference strains representing the five *Metarhizium* PARB clade species (Supplementary Figure S1), isolates LBM-30, LBM-41 and LBM-42, together with the identified entries were located within the clade comprising all *M. pinghaense* reference strains. However, this *M. pinghaense* clade received low (79%) bootstrap support at its root and lacked well supported sub-clades. 

**Figure 1:**
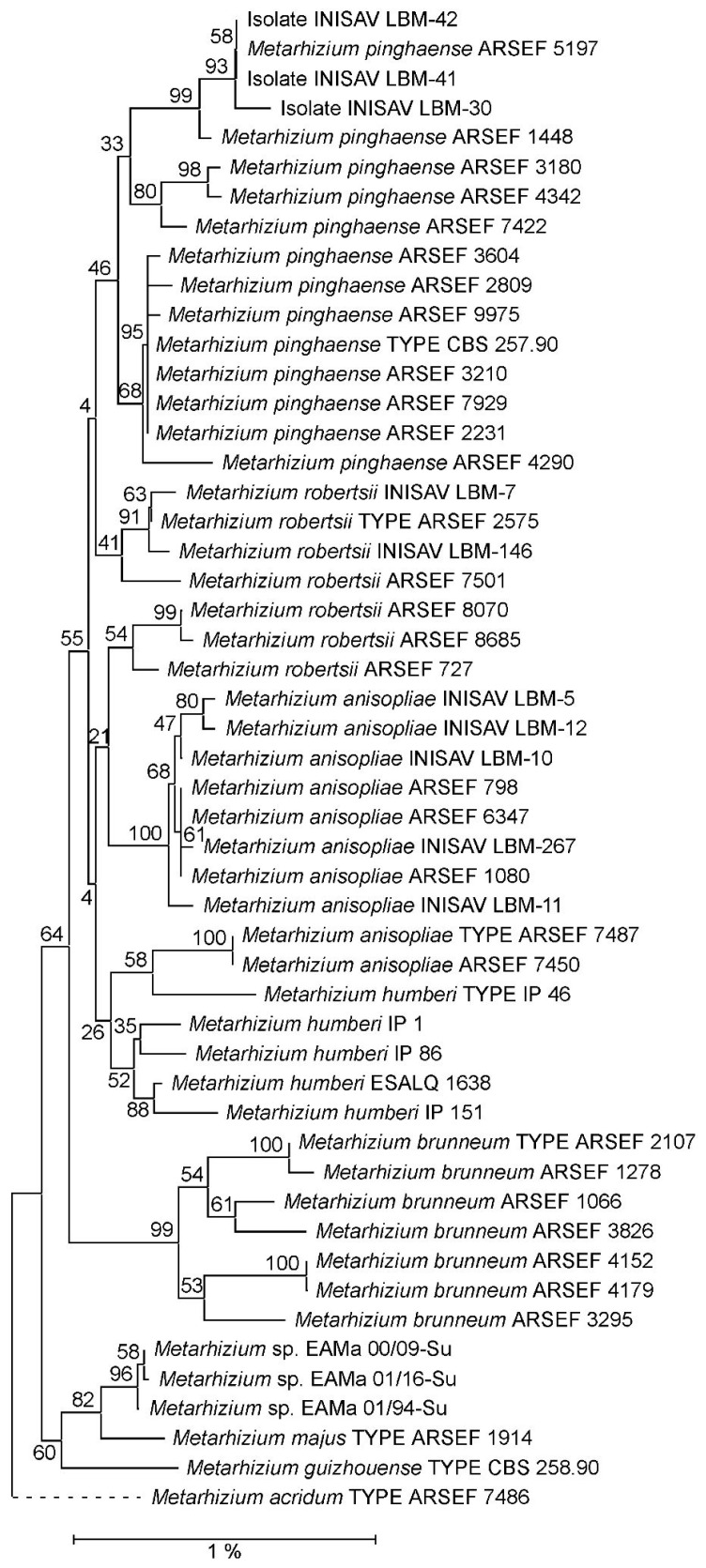
Neighbor joining (NJ) phylogeny of *Metarhizium* fungi as reconstructed from a concatenation of EF1A, RPB1 and RPB2 marker sequences. Terminal branches are labelled by genus, species and strain designations; “TYPE” denotes the nomenclatural-type strain of a species. Numbers on branches indicate bootstrap support percentages. The size bar corresponds to 1% sequence divergence; branches drawn as dashed lines are not to scale. A concatenation of the orthologous sequences from the *M. acridum* type strain has been used as outgroup.

In contrast, in the NJ phylogeny reconstructed from concatenated EF1A, RPB1 and RPB2 marker sequences (Figure 1), isolates LBM-30, LBM-41 and LBM-42, together with strains ARSEF 1448 and ARSEF 5197, but not ARSEF 3180 and ARSEF 4290, formed a 99% bootstrap-supported sub-clade within an overall ill-supported *M. pinghaense* clade. In the corresponding ML phylogeny (Supplementary Figure S2) this sub-clade appeared even in a sister-clade position with respect to the presumed *M. pinghaense* clade. However, as has been observed previously [19], PARB clade species were insufficiently resolved in phylogenies reconstructed from EF1A–RPB1–RPB2 concatenations.

On the basis of the above analyses, isolates LBM-30, LBM-41 and LBM-42 were provisionally identified as *Metarhizium pinghaense sensu lato*. As the presumably most closely related available specimens, strain ARSEF 1448, originally isolated from the burrowing bug, *Scaptocoris castanea*, in Goiás (Brazil), and strain ARSEF 5197, isolated from the sugarcane rootstalk borer weevil, *Diaprepes abbreviata* in Florida (USA), were included in the downstream analyses.

### 3.2. Molecular Taxonomic Identification Based on Intergenic Spacer (IGS) Sequences

Differences in ribosomal intergenic spacer (rIGS) sequences have previously been developed into a diagnostic PCR tool discriminating between *Metarhizium* PARB clade species [19]. When probed with the corresponding diagnostic primer pairs (Supplementary Table S3), the five strains under study gave negative results not only for *M. anisopliae*, *M. brunneum* and *M. robertsii*, but also for *M. pinghaense*, which is an outcome in conflict with the provisional species level assignment (Supplementary Figure S3). In order to conclusively identify the strains at the taxonomic species level, further genetic markers were investigated.

Amplification and sequencing of the rIGS-ID800 element that had previously been established as molecular marker for *Metarhizium* PARB clade species identification [19], gave rise to an identical marker sequence comprising a length of 785 bp from strains LBM-30, LBM-41, LBM-42 and ARSEF 5197, whereas rIGS-ID800 from ARSEF 1448 (788 bp) was determined to be 98.3% similar. In the NJ phylogeny reconstructed from rIGS-ID800 marker sequences (Supplementary Figure S4), these five strains formed a 100% bootstrap supported sub-clade developing out of the supposed *M. pinghaense* clade. It has been observed previously [19] that rIGS-ID800, when used as single marker, supported the monophyly of the further PARB species (99–100% support in Supplementary Figure S4), whereas *M. pinghaense* was represented by an ill-supported (58%), apparently loose association of several sub-clades, consistent with the taxon *M. pinghaense* being polyphyletic.

Moreover, PCR amplification of the molecular–taxonomic MzIGS3 (or DUF895) marker from the three Cuban strains under study gave rise to almost identical (i.e., >99.9% pairwise similarities) consensus sequences comprising a length of 1012 bp. When used as query for an unfiltered BlastN search across the GenBank database, the MzIGS3 marker from strain LBM-41 identified 163 entries of at least 96.0% similarity (at not less than 90% coverage) comprising the bulk of MzIGS3 database entries assigned to the species *M. pinghaense*. MzIGS3, from ARSEF 5197, was identified as unique identical best hit (displaying 100% similarity at 100% coverage), followed by the DUF895 sequence from ARSEF 1448 as single second-best hit of 99.7% (100%) and a plethora of entries at <99.0% sequence similarity. In the NJ phylogeny reconstructed from these identified MzIGS3 sequences (Supplementary Figure S5), strains LBM-30, LBM-41 and LBM-42 together with ARSEF 1448 and ARSEF 5197 formed an optimally supported sub-clade within the *M. pinghaense* clade; no further DUF895 sequences from the GenBank database were part of this clade. However, it is clear from Supplementary Figure S5 that—as has been stated previously [20]—when used as single marker, MzIGS3 does not sufficiently resolve the species *M. anisopliae*, *M. humberi* and *M. robertsii*.

**Figure 2:**
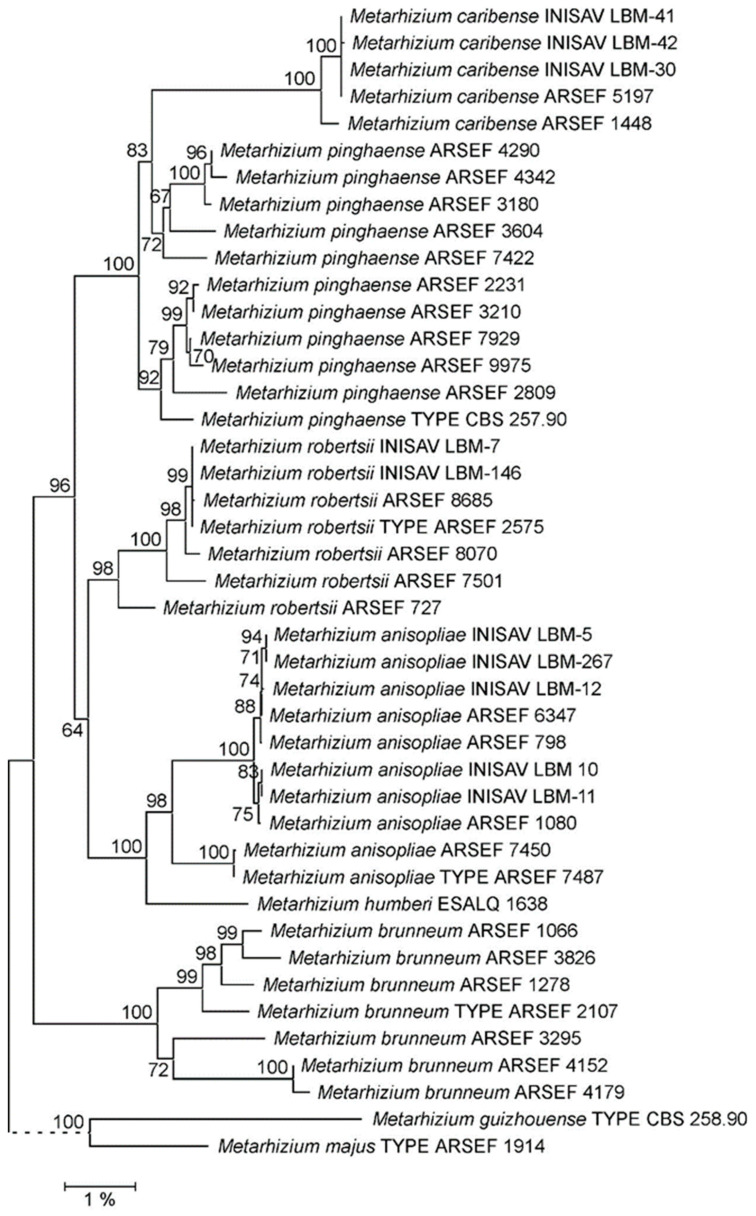
Neighbor joining (NJ) phylogeny of *Metarhizium* fungi as reconstructed from a concatenation of 5TEF, DUF895 (*MzIGS3*) and rIGS-ID800 marker sequences. Terminal branches are labelled by genus, species and strain designations; “TYPE” denotes the nomenclatural-type strain of a species. Numbers on branches indicate bootstrap support percentages. The size bar corresponds to 1% sequence divergence; branches drawn as dashed lines are not to scale. Concatenations of the orthologous sequences from the *M. majus-* and *M. guizhouense*-type strains have been used as outgroup.

In order to obtain a conclusive species assignment for the Cuban isolates, NJ and ML phylogenies were reconstructed from the concatenation of non-coding markers 5TEF, MzIGS3 and rIGS-ID800 (Figure 2, Supplementary Figure S6). In both phylogenies, PARB species clades appeared solidly supported. However, the presumed *M. pinghaense* clade, receiving optimal (100%) bootstrap support at its root in both the NJ and ML tree, comprised a long-branched and, by itself, 100% bootstrap-supported sub-clade made up of strains LBM-30, LBM-41, LBM-42, ARSEF 1448 and ARSEF 5197. However, bootstrap support for inclusion of this sub-clade into the *M. pinghaense* clade was only about 80% in both trees. Both the NJ and ML tree topologies were, therefore, prima facie consistent with both the five strains under study forming (i) a sub-species of the species *M. pinghaense* (*sensu stricto*) or (ii) an independent species within an *M. pinghaense* (*sensu lato*) species complex.

A comparison of pairwise sequence similarities was employed to evaluate this topological finding within the context of *Metarhizium* PARB species delineation. When calculating pairwise sequence similarity percentages for the 5TEF—MzIGS3—rIGS-ID800 marker concatenation from a p-distance matrix (Supplementary Table S4), sequences from strains LBM-30, LBM-41, LBM-42 and ARSEF 5197 were almost identical (99.9–100% similarity) and very similar to that of strain ARSEF 1448 (99.4–99.5%). Employed as a representative of these five tightly clustering strains, strain ARSEF 5197 displayed 96.1% to 96.5% pairwise sequence similarity to the nomenclatural-type strain and further reference strains of the species *M. pinghaense*. These values are in the same range as the inter-specific pairwise similarities between the type and reference strains of the PARB species *M. pinghaense*, *M. anisopliae* and *M. robertsii* (96.2–96.9%), but are considerably lower than intra-specific pairwise similarities between the *M. pinghaense* type and further reference strains (97.8–99.1%). Taken together, pairwise sequence similarity analysis indicates that the phylogenetic relationship of strain ARSEF 5197 and the four further strains under study to the nomenclatural-type strain of the species *M. pinghaense* are inter- rather than intra-specific.

### 3.3. rIGS-Based Species-Discriminating Diagnostic PCR

**Figure 3:**
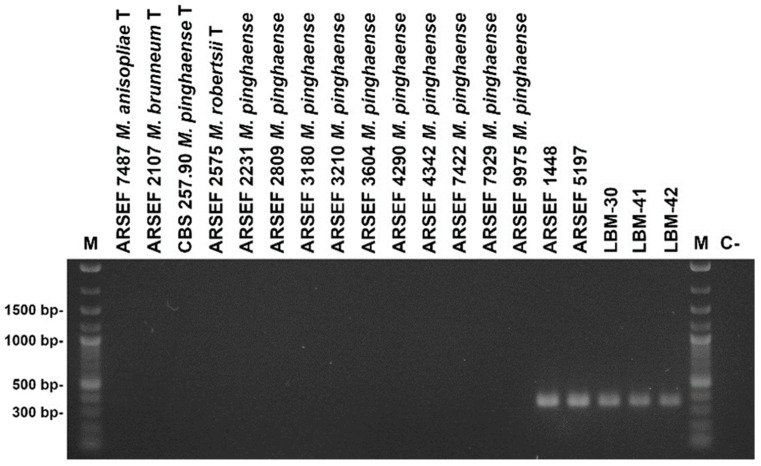
Agarose gel electrophoresis of diagnostic PCRs using primer pair mcar-IDF1/mcar-IDR1 (Supplementary Table S3) specific for the species *Metarhizium caribense*. The length of relevant signals in the size standard is indicated at the left margin. Lane labels on top of the picture designate the *Metarhizium* species, strain or isolate; “**T**” indicates nomenclatural type strains, “**M**” denotes the size standard, and “**C-**” denotes negative (no template) control.

In order to extend the previously developed rIGS-based species-discriminating diagnostic PCR tool to the presumed new *Metarhizium* species, complete rIGS sequences comprising lengths of between 1593 bp and 1602 bp were determined for strains LBM-30, LBM-41, LBM-42, ARSEF 1448 and ARSEF 5197 and were carefully compared with those of *Metarhizium* PARB clade reference strains. Species-specific sequence features, such as InDels or SNPs, were identified and pairs of oligonucleotide primers of varying length comprising these specific sequence features were designed (Supplementary Figure S7) and validated under pre-established diagnostic PCR parameters for functionality and discriminative power. Following this rationale gave rise to the definition of a pair of species-discriminating diagnostic PCR primers for the new species: mcar-IDF1 5′-GGACTTGGCATATTTGCTTGAATTG and mcar-IDR1 5′-TCTTATATACCCACCAACTACCTTG (Supplementary Table S3). When employed with the previously established diagnostic PCR protocol, the primer pair unambiguously discriminated between the five strains under study, on the one hand, and the *Metarhizium* PARB species-type strains together with further *M. pinghaense* reference strains, on the other hand, amplifying partial rIGS sequences exclusively from cognate genomic DNA templates (Figure 3).

### 3.4. 28S Ribosomal RNA Gene Group-I Intron Analysis

**Figure 4:**
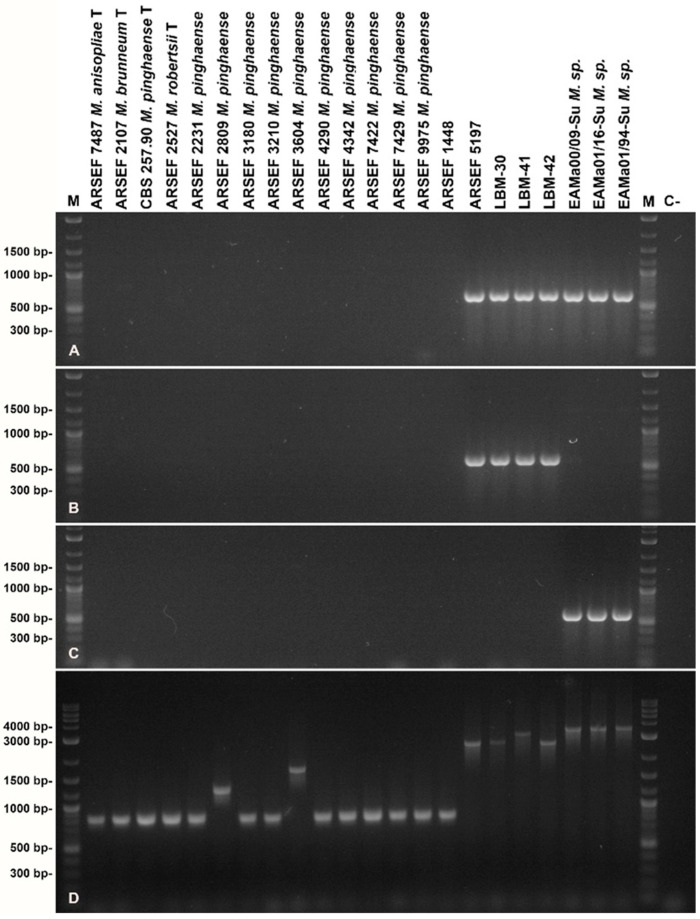
Agarose gel electrophoresis of diagnostic PCR amplification of 28Si4 intron sequences using primer pairs Meta28Si4long-F1/28Si-E24Rshort (**A**), Mcar28Si4long-F1/28Si-E24Rshort (**B**), and Mmaj28Si4long-F1/28Si-E24Rshort (**C**) (Supplementary Table S3) as well as the complete 28S rRNA gene intron insertion region (**D**). The length of relevant signals in the size standard is indicated at the left margin. Lane labels on top of the picture designate the *Metarhizium* species, strain or isolate; “**T**” indicates nomenclatural type strains, “**M**” denotes the size standard, and “**C-**” denotes negative (no template) control.

Amplification of the 28S rRNA gene intron insertion region from fungal isolates was consistent with the presence of copies of self-splicing group-I introns in strains LBM-30, LBM-41, LBM-42 and ARSEF 5197, but not in ARSEF 1448 (Figure 4D). Sequencing of the PCR products revealed the presence of an identical intron, termed 28Si4, comprising 1751 bp in insertion position 4 of the 28S rRNA gene of strains LBM-30, LBM-41, LBM-42 and ARSEF 5197, but not ARSEF 1448. In addition, LBM-41 was found to carry a second intron of 438 bp in position 1. None of the *Metarhizium* PARB species-type strains and further *M. pinghaense* reference strains analyzed carried a similar long intron in the 28S rRNA intron region. Though strains ARSEF 2809 and ARSEF 3604 carried smaller introns comprising app. 400 bp of unrelated sequence in positions 1 and 4, further reference strains did not carry 28S rRNA introns.

When used as query for unfiltered BlastN searches across the GenBank database, the 28Si4 sequence from ARSEF 5197 identified two highly similar (98.5% similarity at 100% sequence coverage) intron sequences residing in position 4 of the 28S rRNA gene of *Metarhizium* isolates from different locations in Spain that had previously been assigned to the *Metarhizium anisopliae* complex [30]. However, by means of phylogenetic reconstruction using 5TEF and concatenated EF1A-RPB1-RPB2 markers, the Spanish isolates EAMa 00/09-Su, EAMa 01/16-Su and EAMa 01/94-Su, carrying a copy of the long 28S rRNA intron, were found to be more closely related to *M. majus* and *M. guizhouense* than to the PARB clade species (Figure 1; Supplementary Figures S1 and S2). The 28Si4 introns identified in these isolates were almost identical to each other (99.9–100% similarity). No further sequence entries of high similarity to the 28Si4 intron from ARSEF 5197 were identified in the GenBank database.

Given that the length of autocatalytic group-I introns disrupting fungal rRNA genes usually ranges from app. 300 bp to 500 bp [24,25], the >1700 bp-long 28Si4 introns identified might be expected to have increased in length by acquisition of homing endonuclease genes [42,43]. A search for open reading frames (ORFs) along both directions of the 28Si4 sequence revealed a single, potentially protein-encoding ORF, comprising 1077 bp in collinear orientation with respect to the 28S rRNA gene. A similar ORF encoding a protein of 97% deduced amino acid sequence identity was carried by the homologous introns from Spanish *Metarhizium* isolates. A search for homologous GenBank entries identified, at a low similarity level, several hypothetical fungal proteins of unknown function. However, no similarities to any of the four types of homing endonucleases and their characteristic motifs [42] became obvious from this analysis.

In depth comparative analysis of 28Si4 intron sequences (Supplementary Figure S8) enabled the design of diagnostic PCR primers (i) Meta28Si4long-F1 for the identification of related long 28Si4 introns in *Metarhizium* and potentially other fungi, and (ii) Mcar28Si4long-F1 and Mmaj28Si4long-F1 for the discrimination of the two intron variants present in strains LBM-30, LBM-41, LBM-42 and ARSEF 5197, on the one hand, and the Spanish *M. majus* related strains, on the other (Supplementary Table S3). Application of these primers in respective diagnostic PCR analyses gave rise to the expected results (Figure 4A–C).

### 3.5. Morphological and Cultural Characterization of Fungal Isolates

**Figure 5:**
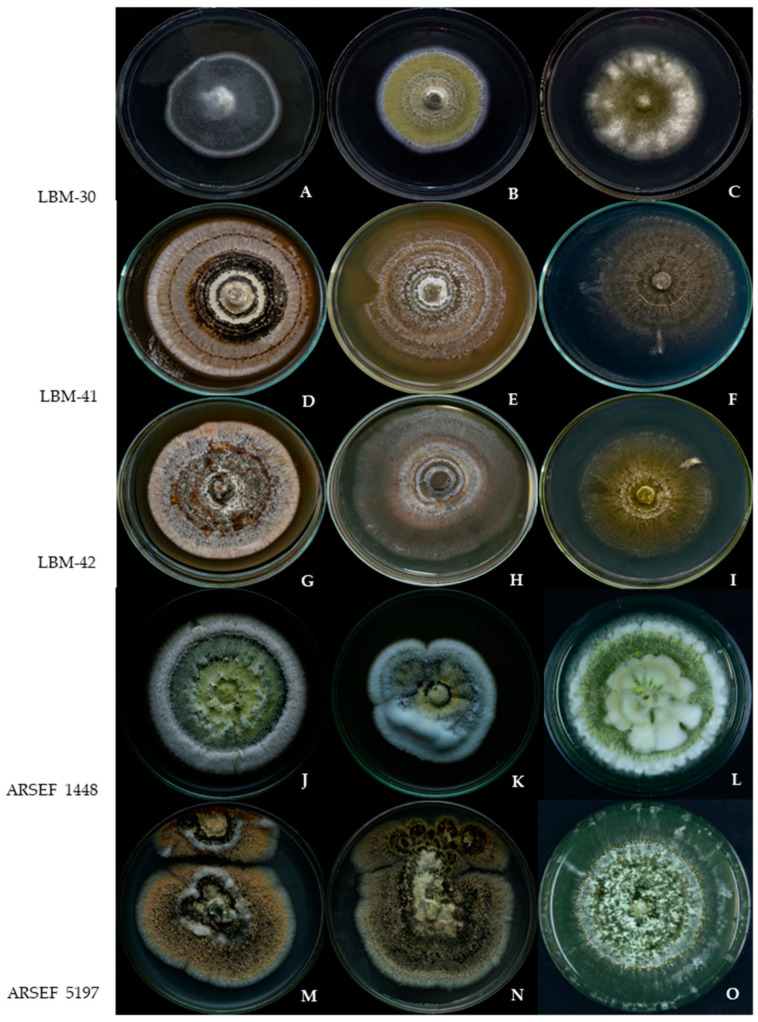
Morphological characteristics of strains LBM-30, LBM-41, LBM-42, ARSEF 1448 and ARSEF 5197 on different culture media, after 14 days of growth at 26 ± 1 °C. Sabouraud dextrose agar (**A**,**D**,**G**,**J**,**M**); complete medium (**B**,**E**,**H**,**K**,**N**); potato dextrose agar (**C**,**F**,**I**,**L**,**O**).

In general, the fungal isolates under study produced cottony colonies with white mycelium that became colored with the development of conidia varying from yellow, olive green, greenish to dark green. The conidiation was slight or formed halos or concentric rings. The colony reverse was colorless or ochreous. Phenotypic differences in colony appearance were observed in different culture media (Figure 5). Conidiophores were broadly branched (like candelabra) and phialides were organized in compact palisades. The conidial shape was cylindrical to ellipsoidal. Morphological characteristics of individual strains are described in Table 2 (Supplementary Figure S9).

## 4. Taxonomic Description

*Metarhizium caribense* Y. Baró, C. Schuster, R.F. Castañeda and A. Leclerque, sp. nov.

Index Fungorum IF 901982.

Etymology: Latin, *caribense* refers to the Caribbean, the original locality of the majority of strains isolated.

**Figure 6:**
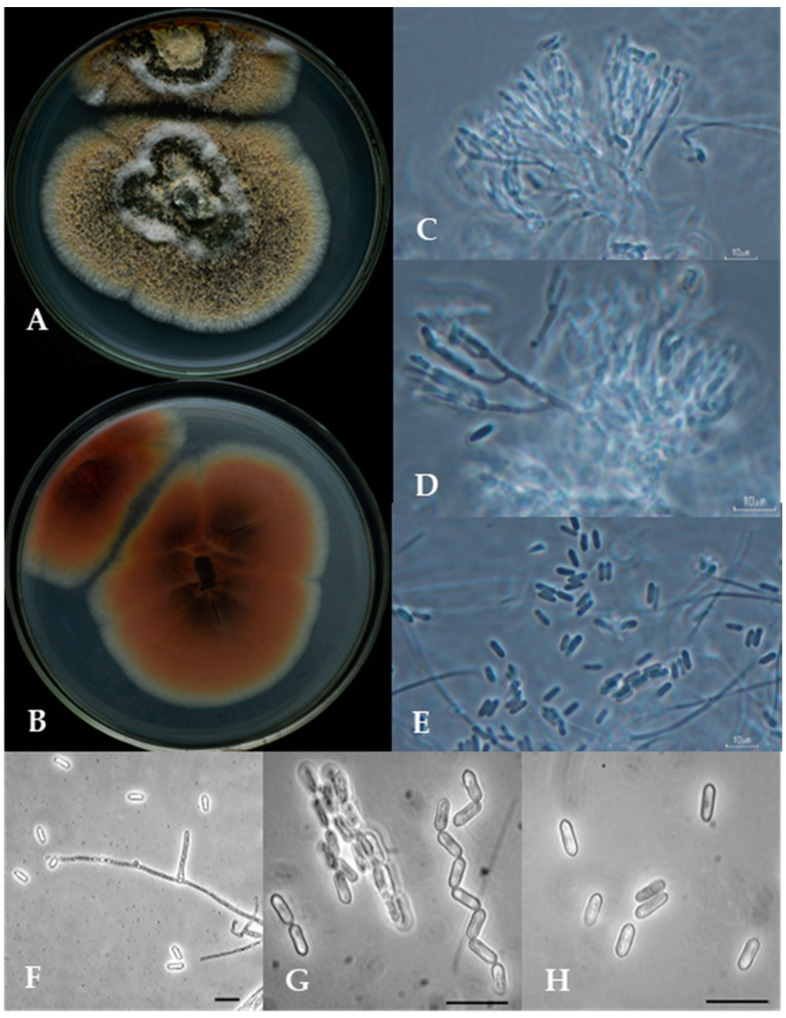
Morphological characteristics of the proposed *Metarhizium caribense* type strain ARSEF 5197. Colony on SDA. (**A**) anverse, (**B**) reverse (**C**) conidiophore, (**D**) phialides, and (**E**–**H**) conidia. All scale bars correspond to 10 µm.

Morphological characteristics: Colonies on SNA after 10 days at 25 ± 2 °C, mycelium mostly immersed, citrine [21K], reverse. Conidiomata sporodochia, columnar, chandelier-like, scattered, arising from vegetative hyphae. Mycelium immersed, composed of branched, hyaline, smooth-walled hyphae. Conidiophores macronematous, densely fasciculate, slightly penicillate branched at the apex, smooth, hyaline. Conidiogenous cells monophialidic, terminal in branches, cylindrical, discrete, smooth, forming a penicillate cluster. Conidia basocatenate, cylindrical, unicellular, smooth-walled, hyaline to olivaceous to green, dry, 5.08–7.04 × 1.22–2.39 µm (Figure 6).

Holotype: Ex-type metabolically inactive culture: deposited as ARSEF 5197 in the USDA-ARS Collection of Entomopathogenic Fungal Cultures (Ithaca, New York, NY, USA) by M. Browning, 20 May 1996; collected by C.W. McCoy.

Host type: *Diaprepes abbreviata* (Coleoptera: Curculionidae).

Type locality: Florida (USA).

Additional specimens examined as follows: strain INISAV LBM-30, La Habana province (Cuba), isolated with the *Galleria* bait method from soil sample; strains INISAV LBM-41 and LBM-42, Granma province (Cuba), isolated from *Hypothenemus hampei* (Coleoptera: Curculionidae); strain ARSEF 1448, Goiatuba (Goiás, Brazil), isolated from *Scaptores castanea* (Hemiptera: Cydnidae) (Supplementary Figure S10).

*M. caribense* cannot be distinguished from most closely related taxa based on conidial and phialidic morphologies alone.

Genetic characteristics: Species-discriminating diagnostic PCR employing the primer pair mcar-IDF1 (5′-GGACTTGGCATATTTGCTTGAATTG) and mcar-IDR1 (5′-TCTTATATACCCACCAACTACCTTG) amplifies a partial sequence of the ribosomal intergenic spacer (rIGS) giving rise to a product of app. 350 bp apparent size exclusively from genomic DNA of *M. caribense.*

## 5. Discussion

Three fungal strains, designated LBM-30, LBM-41 and LBM-42, that had previously been isolated in Cuba within the framework of a monitoring program aiming towards the identification of new microbial control agents, were investigated in this study. Using morphological taxonomic criteria together with genetic markers (EF1A, RPB1, RPB2) established for the systematics of Hypocreales fungi, the strains were demonstrated to belong to the genus *Metarhizium* and to be most closely related to the two reference strains, ARSEF 1448 and ARSEF 5197, that had previously been assigned to the PARB clade species *M. pinghaense*. However, species assignment remained inconclusive at this level of analysis, and LBM-30, LBM-41 and LBM-42 were provisionally designated as *M. pinghaense sensu lato*.

In depth analysis of the systematic position of these fungi using genetic markers (5TEF, MzIGS3, rIGS) established for the *M. anisopliae* species complex or the PARB clade, demonstrated that the three Cuban isolates, together with strains ARSEF 1448 and ARSEF 5197, formed a statistically well-supported sub-clade within the PARB clade, but apart from further well-supported *Metarhizium* PARB species. Pairwise sequence distance analysis motivated the proposal of a new *Metarhizium* species termed *M. caribense*, with reference strain ARSEF 5197 being proposed as nomenclatural type of the new taxon. Application of diagnostic PCR using species-specific primer pairs lent further support to this interpretation of the molecular taxonomic data.

*M. caribense* reference strains ARSEF 5197 and ARSEF 1448 have been studied previously under different perspectives. A broad study of Jin et al. [44] evaluated the pathogenicity against nymphs of the brown planthopper, *Nilaparvata lugens*, of 35 *Metarhizium* strains, including ARSEF 5197, with the latter showing only moderate virulence (about 43%) against this pest. Leadmon et al. [45] attributed the virulence of several *Metarhizium* strains to the secretion of ergot alkaloids in infected insects; however, the strain ARSEF 5197 included in this survey was found to be negative for ergot alkaloids. The authors highlight the importance of these metabolites in pathogenesis, indicating a positive correlation between *Metarhizium* ergot alkaloid accumulation and insect colonization. In addition to ergot alkaloids, *Metarhizium* fungi produce other molecules, such as destruxins (DTXs), that contribute to insecticidal activity. Golo et al. [46] evaluated the potential correlation of virulence and DTX production of a set of 20 *Metarhizium* strains comprising ARSEF 1448. The latter was found to be highly virulent to *Tenebrio molitor* but did not produce any of the assessed destruxin types, A, B or E.

Previous research had demonstrated the pathogenicity of the three Cuban isolates under study for adults of the sweet potato weevil [32]. Strain LBM-30 has been shown to produce and secrete hydrolytic enzymes as chitinases, lipases, proteases, amylases and caseinases that are generally supposed to contribute to the high virulence of entomopathogenic fungi [32]. Moreover, the strain displays appropriate parameters of conidiation, conidial yield and germination as well as growth in different culture media, making it a potential target for biocontrol agent development. Concomitant research has identified a second Cuban strain, *M. anisopliae* LBM-267, as a potential microbial control agent targeting the sweet potato weevil [31]. *C. formicarius* is the most destructive pest affecting the tropical and subtropical production of sweet potatoes (*Ipomoea batatas*) both in the field and during storage. The larval and adult stages of the weevil have a significant economic impact, causing yield or crop losses up to 100% [47,48]. Currently, control of *C. formicarius* relies mainly on chemical insecticides. However, due to negative side effects of agrochemicals and the pest’s soil inhabiting lifestyle, the use of insect pathogenic fungi as part of integrated pest management programs is highly solicited and increases each year. In particular, *Metarhizium* and *Beauveria* fungi have been used for biological control of this pest. 

Future research directions for bio-insecticide developments for the control of the sweet potato weevil will focus on formulation, field experiments and risk assessment with both *M. caribense* LBM-30 and *M. anisopliae* LBM-267, including evaluation of possible synergistic effects of a binary product comprising both strains together.

At the methodological level, the present study has demonstrated the potential of the ribosomal intergenic spacer (rIGS) sequence, both as single marker and in combination with 5TEF and MzIGS3 sequences, for species delineation within the *Metarhizium* PARB clade. The recently developed rIGS-based species-discriminating PCR approach covering the original four PARB clade species [19] and a further PCR tool based on the presence of self-splicing group-I introns in fungal 28S rRNA genes [27,28,29] have been extended to *M. caribense*, enabling the future sequencing-independent identification of further strains belonging to this new species.

## 6. Conclusions

Within the PARB clade of the taxonomic genus *Metarhizium*, a new entomopathogenic fungal species was introduced as consequence of an in-depth multigene phylogenetic analysis of three fungal isolates from Cuba, together with two previously described *Metarhizium* strains from Florida and Brazil. The species epithet “*caribense*”, referring to four of these isolates’ geographic origin, was proposed for the new species, and strain ARSEF 5197 was described as a nomenclatural-type strain of the new taxon. A previously established rIGS-based diagnostic PCR tool has been extended to the new species and has been shown to enable sequencing- independent species discrimination within the *Metarhizium anisopliae* complex. 

## Figures and Tables

**Table 1:** Fungal isolates from the INISAV culture collection investigated in this study.

Strain Designation	Source of Isolation	Geographic Origin
LBM-30	Soil using *Galleria mellonella* (L.) (Lepidoptera: Pyralidae) bait method	La Habana, Cuba
LBM-41	Coffee berry borer, *Hypothenemus hampei* Ferrari (Coleoptera: Curculionidae)	Granma, Cuba
LBM-42	Coffee berry borer, *Hypothenemus hampei* Ferrari (Coleoptera: Curculionidae)	Granma, Cuba

**Table 2:** Morphological characteristics of fungal strains studied when grown on SDA.

Isolate	Colony Characteristics	Conidial Size ^a^	Conidial Shape
LBM-30	Cottony texture, abundant mycelia growth and wooly in the central area, with a white mycelial margin. Abundant conidiation yellow to olive green in color and crusty appearance, with regular border. Conidiophores broadly branched (chandelier-like)	5.00–8.50 (6.60) × 2.00–3.00 (2.44)	Cylindrical
LBM-41	White colonies to greenish olivaceous, forming halos or concentric rings with conidia maturation. Abundant conidiation in central area forming small pustules, with white mycelial margin. Ochreous pigment diffusing into medium. Conidiophores broadly branched (chandelier-like).	4.88–7.32 (5.66) × 2.44–3.66 (2.71).	Cylindrical to ellipsoidal
LBM-42	White to dark olivaceous green with conidia maturation, colonies with white mycelial margin. Diffuse conidiation forming small pustules. Ochreous pigment diffusing into medium	4.88–7.32 (6.93) × 2.43–2.44 (2.44).	Cylindrical to ellipsoidal
ARSEF 1448	Colonies were white and became yellow to olive green with a white mycelial margin. Colony reverses were yellow. Conidiophores were broadly branched, like a candelabrum arrangement. Phialides were cylindrical and organized in compact palisades.	4.91–7.19 (5.99) × 1.29–2.56 (1.83).	Cylindrical
ARSEF 5197	Colonies initially appeared white and became yellow to dark olive green with white mycelial margin. Colony reverses were ochreous. Conidiophores were broadly branched, like a candelabrum arrangement. Phialides were cylindrical and organized in compact palisades	5.08–7.04 (5.78) × 1.22–2.39 (1.82)	Cylindrical

^a^ All measurements in μm, length × width. Average values are indicated in brackets.

## Data Availability

Sequence data analyzed in this study are publicly available from the GenBank database (https://www.ncbi.nlm.nih.gov; accessed 1 July 2024) under nucleotide sequence accession numbers listed in Supplementary Table S1 to this study.

## Supplementary Materials

The following supporting information can be downloaded at: https://www.mdpi.com/article/10.3390/jof10090612/s1. Supplementary Figure S1: Neighbor-Joining (NJ) phylogeny of *Metarhizium* fungi as reconstructed from 5TEF marker sequences. Supplementary Figure S2: Maximum Likelihood (ML) phylogeny of *Metarhizium* fungi as reconstructed from a concatenation of EF1A, RPB1 and RPB2 marker sequences. Supplementary Figure S3: Agarose gel electrophoresis of diagnostic PCRs using *Metarhizium* PARB species-discriminating primer pairs. Supplementary Figure S4: Neighbor-Joining (NJ) phylogeny of *Metarhizium* fungi as reconstructed from rIGS-ID800 marker sequences. Supplementary Figure S5: Neighbor-Joining (NJ) phylogeny of *Metarhizium* fungi as reconstructed from MzIGS3 (DUF895) marker sequences. Supplementary Figure S6: Maximum likelihood (ML) phylogeny of *Metarhizium* fungi as reconstructed from a concatenation of 5TEF, DUF895 (MzIGS3) and rIGS-ID800 marker sequences. Supplementary Figure S7: Alignment of ribosomal intergenic spacer (rIGS) sequences used for species-discriminating primer design. Supplementary Figure S8: Alignment of 28Si4 intron sequences used for diagnostic primer design. Supplementary Figure S9: Colony growth of *Metarhizium caribense* strain ARSEF 5197 on SNA medium. Supplementary Figure S10: Morphological characteristics of *Metarhizium caribense* strain ARSEF 1448. Supplementary Table S1: Fungal strains and GenBank accession numbers of marker sequences referred to in this study. Supplementary Table S2: Preparative PCR and sequencing primers used, and reaction-specific parameters applied in this study. Supplementary Table S3: Diagnostic PCR primers used in this study. Supplementary Table S4: Pairwise sequence similarity percentages of concatenated 5TEF–MzIGS3–rIGSID800 sequences as calculated from a p-distance matrix. Refs. [49,50,51,52,53,54,55] are cited in the Supplementary Materials.
