# Supplementary material for: Metarhizium caribense sp. nov., a Novel Species of Entomopathogenic Metarhizium Fungi Associated with Weevils Impairing Coffee, Sugar Cane and Sweet Potato Cultivation
Source: J Fungi (Basel). 2024 Aug 28;10(9):612. doi: 10.3390/jof10090612 (PMC11433170; doi:10.3390/jof10090612)
Supplement: Supplementary file 1 [file jof-10-00612-s001.zip › Suppl Figure S1.pdf]

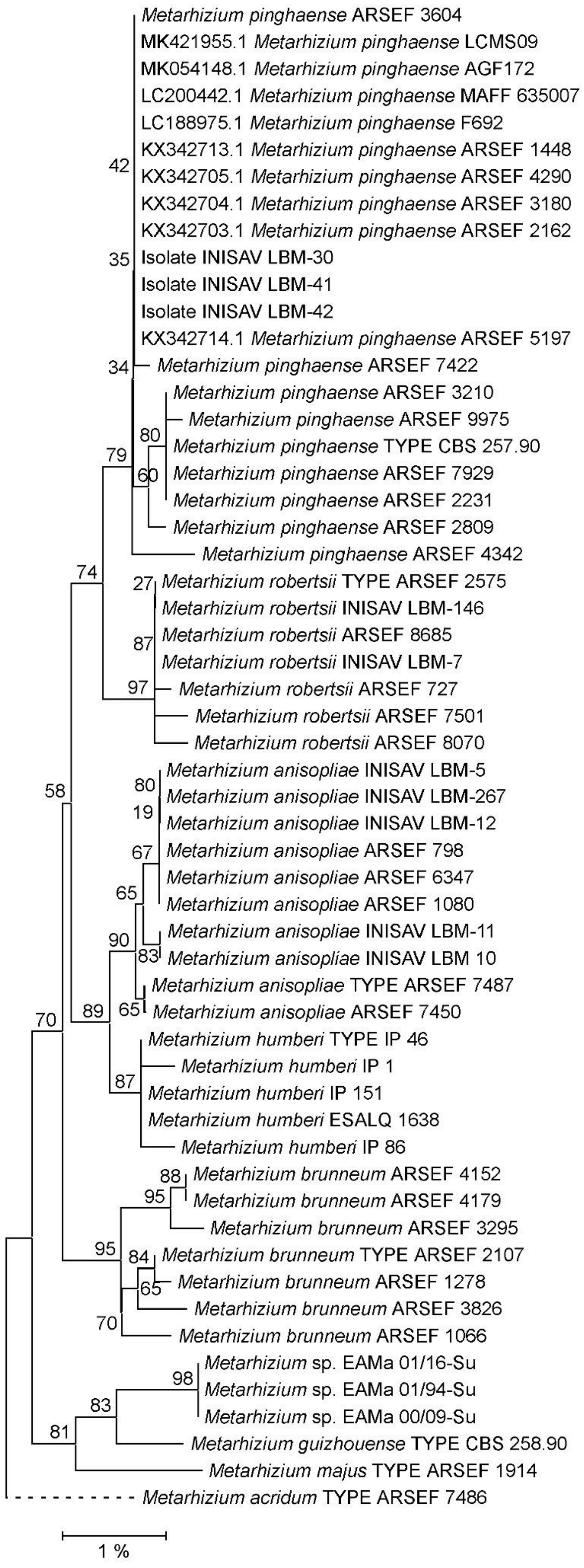

Supplementary Figure S1. Neighbor-Joining (NJ) phylogeny of *Metarhizium* fungi as reconstructed from 5TEF marker sequences. Terminal branches are labelled by genus, species and strain designations; “TYPE” denotes the nomenclatural type strain of a species. Accession numbers are indicated for reference sequences identified in similarity searches across the GenBank database. Numbers on branches indicate bootstrap support percentages. The size bar corresponds to 1 % sequence divergence; branches drawn as dashed lines are not to scale. The orthologous sequence from the *M. acridum* type strain has been used as outgroup.
