# Supplementary material for: Metarhizium caribense sp. nov., a Novel Species of Entomopathogenic Metarhizium Fungi Associated with Weevils Impairing Coffee, Sugar Cane and Sweet Potato Cultivation
Source: J Fungi (Basel). 2024 Aug 28;10(9):612. doi: 10.3390/jof10090612 (PMC11433170; doi:10.3390/jof10090612)
Supplement: Supplementary file 1 [file jof-10-00612-s001.zip › Suppl Figure S10.pdf]

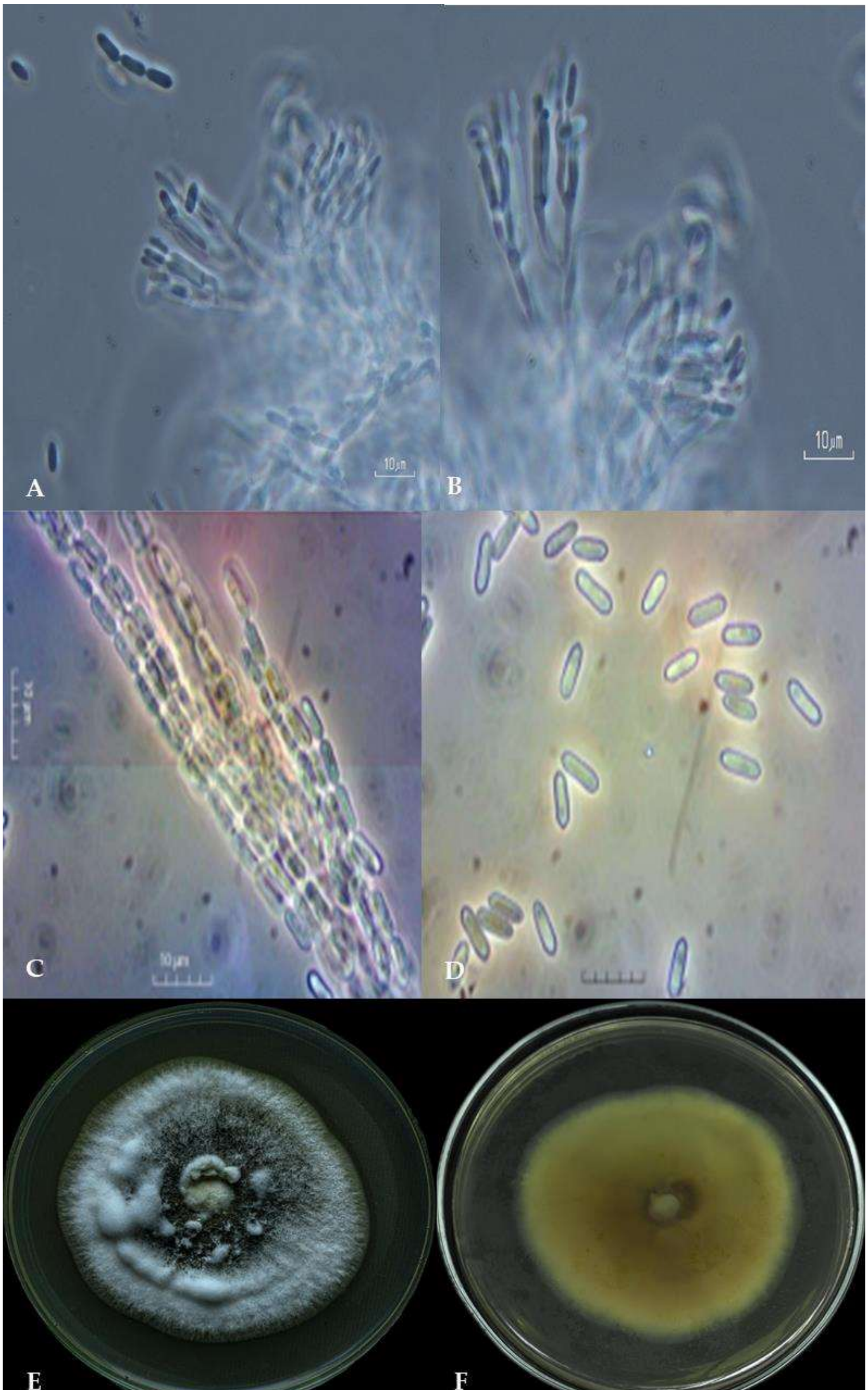

Supplementary Figure S10. Morphological characteristics of *Metarhizium caribense* strain ARSEF 1448. Colony on SDA. A, B: conidiophores; C, D: conidia; E: anverse of colony, F: reverse of colony. All scale bars correspond to 10 μm.
