# Supplementary material for: Metarhizium caribense sp. nov., a Novel Species of Entomopathogenic Metarhizium Fungi Associated with Weevils Impairing Coffee, Sugar Cane and Sweet Potato Cultivation
Source: J Fungi (Basel). 2024 Aug 28;10(9):612. doi: 10.3390/jof10090612 (PMC11433170; doi:10.3390/jof10090612)
Supplement: Supplementary file 1 [file jof-10-00612-s001.zip › Suppl Figure S3.pdf]

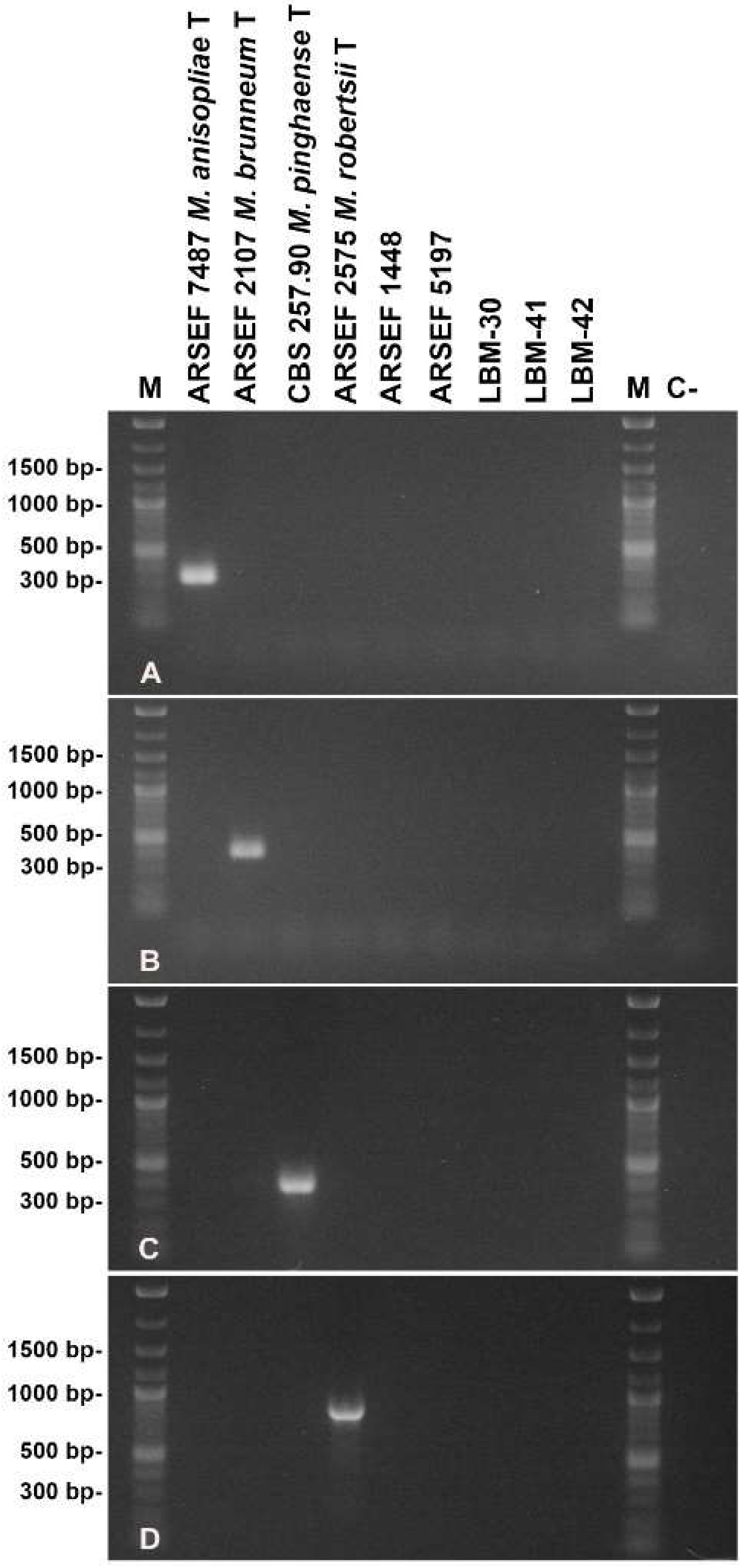

Supplementary Figure S3. Agarose gel electrophoresis of diagnostic PCRs using *Metarhizium* PARB species-discriminating primer pairs (Suppl. Table S3) specific for *M. anisopliae sensu stricto* (**A**), *M. brunneum* (**B**), *M. pinghaense* (**C**), and *M. robertsii* (**D**). The length of relevant signals in the size standard is indicated at the left margin. Lane labels on top of the picture designate the *Metarhizium* species, strain or isolate; "**T**" indicates nomenclatural type strains. "**M**" denotes the size standard, "**C-**" the negative (no template) control.
