# Supplementary material for: Metarhizium caribense sp. nov., a Novel Species of Entomopathogenic Metarhizium Fungi Associated with Weevils Impairing Coffee, Sugar Cane and Sweet Potato Cultivation
Source: J Fungi (Basel). 2024 Aug 28;10(9):612. doi: 10.3390/jof10090612 (PMC11433170; doi:10.3390/jof10090612)
Supplement: Supplementary file 1 [file jof-10-00612-s001.zip › Suppl Figure S5.pdf]

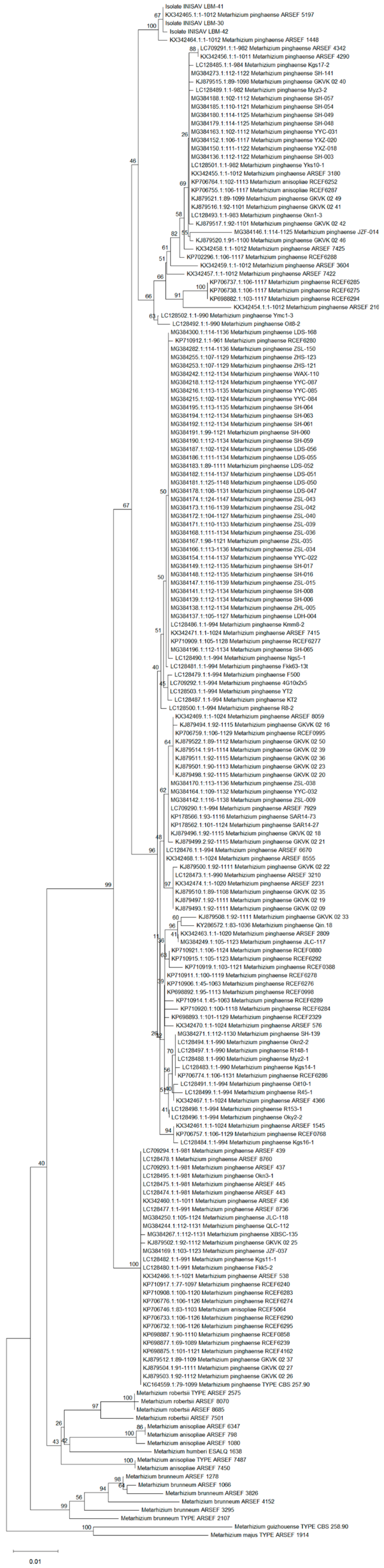
Isolate INISAV LBM-41
KX342465.1:1-1012 Metarhizium pinghaense ARSEF 5197
Isolate INISAV LBM-30
Isolate INISAV LBM-42
KX342464.1:1-1012 Metarhizium pinghaense ARSEF 1448
LC709291.1:1-982 Metarhizium pinghaense ARSEF 4342
KX342456.1:1-1011 Metarhizium pinghaense ARSEF 4290
LC128485.1:1-984 Metarhizium pinghaense Kgs17-2
MG384273.1:112-1122 Metarhizium pinghaense SH-141
KJ879515.1:89-1098 Metarhizium pinghaense GKVK 02 40
LC128489.1:1-982 Metarhizium pinghaense Myz3-2
MG384188.1:102-1112 Metarhizium pinghaense SH-057
MG384185.1:110-1121 Metarhizium pinghaense SH-054
MG384180.1:114-1125 Metarhizium pinghaense SH-049
MG384179.1:114-1125 Metarhizium pinghaense SH-048
MG384163.1:102-1112 Metarhizium pinghaense YYC-031
MG384152.1:106-1117 Metarhizium pinghaense YXZ-020
MG384150.1:111-1122 Metarhizium pinghaense YXZ-018
MG384136.1:112-1122 Metarhizium pinghaense SH-003
LC128501.1:1-982 Metarhizium pinghaense Yks10-1
KX342455.1:1-1012 Metarhizium pinghaense ARSEF 3180
KP706764.1:102-1113 Metarhizium anisopliae RCEF6252
KP706755.1:106-1117 Metarhizium anisopliae RCEF6287
KJ879521.1:89-1099 Metarhizium pinghaense GKVK 02 49
KJ879516.1:92-1101 Metarhizium pinghaense GKVK 02 41
LC128493.1:1-983 Metarhizium pinghaense Okn1-3
KJ879517.1:92-1101 Metarhizium pinghaense GKVK 02 42
MG384146.1:114-1125 Metarhizium pinghaense JZF-014
KJ879520.1:91-1100 Metarhizium pinghaense GKVK 02 46
KX342458.1:1-1012 Metarhizium pinghaense ARSEF 7425
KP702296.1:106-1117 Metarhizium pinghaense RCEF6288
KX342459.1:1-1012 Metarhizium pinghaense ARSEF 3604
KX342457.1:1-1012 Metarhizium pinghaense ARSEF 7422
KP706737.1:106-1117 Metarhizium pinghaense RCEF6285
KP706738.1:106-1117 Metarhizium pinghaense RCEF6275
KP698882.1:103-1117 Metarhizium pinghaense RCEF6294
KX342454.1:1-1012 Metarhizium pinghaense ARSEF 216
LC128502.1:1-990 Metarhizium pinghaense Ymc1-3
LC128492.1:1-990 Metarhizium pinghaense Oit8-2
MG384300.1:114-1136 Metarhizium pinghaense LDS-168
KP710912.1:1-961 Metarhizium pinghaense RCEF6280
MG384282.1:114-1136 Metarhizium pinghaense ZSL-150
MG384255.1:107-1129 Metarhizium pinghaense ZHS-123
MG384253.1:107-1129 Metarhizium pinghaense ZHS-121
MG384242.1:112-1134 Metarhizium pinghaense WAX-110
MG384218.1:112-1124 Metarhizium pinghaense YYC-087
MG384216.1:113-1135 Metarhizium pinghaense YYC-085
MG384215.1:102-1124 Metarhizium pinghaense YYC-084
MG384195.1:113-1135 Metarhizium pinghaense SH-064
MG384194.1:112-1134 Metarhizium pinghaense SH-063
MG384192.1:112-1134 Metarhizium pinghaense SH-061
MG384191.1:99-1121 Metarhizium pinghaense SH-060
MG384190.1:112-1134 Metarhizium pinghaense SH-059
MG384187.1:102-1124 Metarhizium pinghaense LDS-056
MG384186.1:111-1134 Metarhizium pinghaense LDS-055
MG384183.1:89-1111 Metarhizium pinghaense LDS-052
MG384182.1:114-1137 Metarhizium pinghaense LDS-051
MG384181.1:125-1148 Metarhizium pinghaense LDS-050
MG384178.1:108-1131 Metarhizium pinghaense LDS-047
MG384174.1:124-1147 Metarhizium pinghaense ZSL-043
MG384173.1:116-1139 Metarhizium pinghaense ZSL-042
MG384172.1:104-1127 Metarhizium pinghaense ZSL-040
MG384171.1:110-1133 Metarhizium pinghaense ZSL-039
MG384168.1:111-1134 Metarhizium pinghaense ZSL-036
MG384167.1:98-1121 Metarhizium pinghaense ZSL-035
MG384166.1:113-1136 Metarhizium pinghaense ZSL-034
MG384154.1:114-1137 Metarhizium pinghaense YYC-022
MG384149.1:112-1135 Metarhizium pinghaense SH-017
MG384148.1:112-1135 Metarhizium pinghaense SH-016
MG384147.1:116-1139 Metarhizium pinghaense ZSL-015
MG384141.1:112-1134 Metarhizium pinghaense SH-008
MG384139.1:112-1134 Metarhizium pinghaense SH-006
MG384138.1:112-1134 Metarhizium pinghaense ZHL-005
MG384137.1:105-1127 Metarhizium pinghaense LDH-004
LC128486.1:1-994 Metarhizium pinghaense Kmm8-2
KX342471.1:1-1024 Metarhizium pinghaense ARSEF 7415
KP710909.1:105-1128 Metarhizium pinghaense RCEF6277
MG384196.1:112-1134 Metarhizium pinghaense SH-065
LC128490.1:1-994 Metarhizium pinghaense Ngs5-1
LC128481.1:1-994 Metarhizium pinghaense Fkk63-13t
LC128479.1:1-994 Metarhizium pinghaense F500
LC709292.1:1-994 Metarhizium pinghaense 4G10x2x5
LC128503.1:1-994 Metarhizium pinghaense YT2
LC128487.1:1-994 Metarhizium pinghaense KT2
LC128500.1:1-994 Metarhizium pinghaense R8-2
KX342469.1:1-1024 Metarhizium pinghaense ARSEF 8059
KJ879494.1:92-1115 Metarhizium pinghaense GKVK 02 16
KP706759.1:106-1129 Metarhizium pinghaense RCEF0995
KJ879522.1:89-1112 Metarhizium pinghaense GKVK 02 50
KJ879514.1:91-1114 Metarhizium pinghaense GKVK 02 39
KJ879511.1:92-1115 Metarhizium pinghaense GKVK 02 36
KJ879501.1:90-1113 Metarhizium pinghaense GKVK 02 23
KJ879498.1:92-1115 Metarhizium pinghaense GKVK 02 20
MG384170.1:113-1136 Metarhizium pinghaense ZSL-038
MG384164.1:109-1132 Metarhizium pinghaense YYC-032
MG384142.1:116-1138 Metarhizium pinghaense ZSL-009
LC709290.1:1-994 Metarhizium pinghaense ARSEF 7929
KP178566.1:93-1116 Metarhizium pinghaense SAR14-73
KP178562.1:101-1124 Metarhizium pinghaense SAR14-27
KJ879496.1:92-1115 Metarhizium pinghaense GKVK 02 18
KJ879499.2:92-1115 Metarhizium pinghaense GKVK 02 21
LC128476.1:1-994 Metarhizium pinghaense ARSEF 6670
KX342468.1:1-1024 Metarhizium pinghaense ARSEF 8555
KJ879500.1:92-1111 Metarhizium pinghaense GKVK 02 22
LC128473.1:1-990 Metarhizium pinghaense ARSEF 3210
KX342474.1:1-1020 Metarhizium pinghaense ARSEF 2231
KJ879510.1:89-1108 Metarhizium pinghaense GKVK 02 35
KJ879497.1:92-1111 Metarhizium pinghaense GKVK 02 19
KJ879493.1:92-1111 Metarhizium pinghaense GKVK 02 09
KJ879508.1:92-1111 Metarhizium pinghaense GKVK 02 33
KY286572.1:83-1036 Metarhizium pinghaense Qin.18
KX342463.1:1-1020 Metarhizium pinghaense ARSEF 2809
MG384249.1:105-1123 Metarhizium pinghaense JLC-117
KP710921.1:106-1124 Metarhizium pinghaense RCEF0880
KP710915.1:105-1123 Metarhizium pinghaense RCEF6292
KP710919.1:103-1121 Metarhizium pinghaense RCEF0388
KP710911.1:100-1119 Metarhizium pinghaense RCEF6278
KP710906.1:45-1063 Metarhizium pinghaense RCEF6276
KP698892.1:95-1113 Metarhizium pinghaense RCEF0998
KP710914.1:45-1063 Metarhizium pinghaense RCEF6289
KP710920.1:100-1118 Metarhizium pinghaense RCEF6284
KP698893.1:101-1129 Metarhizium pinghaense RCEF2329
KX342470.1:1-1024 Metarhizium pinghaense ARSEF 576
MG384271.1:112-1130 Metarhizium pinghaense SH-139
LC128494.1:1-990 Metarhizium pinghaense Okn2-2
LC128497.1:1-990 Metarhizium pinghaense R148-1
LC128488.1:1-990 Metarhizium pinghaense Myz2-1
LC128483.1:1-990 Metarhizium pinghaense Kgs14-1
KP706774.1:106-1131 Metarhizium pinghaense RCEF6286
LC128491.1:1-994 Metarhizium pinghaense Oit10-1
LC128499.1:1-994 Metarhizium pinghaense R45-1
KX342467.1:1-1024 Metarhizium pinghaense ARSEF 4366
LC128498.1:1-994 Metarhizium pinghaense R153-1
LC128496.1:1-994 Metarhizium pinghaense Oky2-2
KX342461.1:1-1024 Metarhizium pinghaense ARSEF 1545
KP706757.1:106-1129 Metarhizium pinghaense RCEF0768
LC128484.1:1-994 Metarhizium pinghaense Kgs16-1
LC709294.1:1-981 Metarhizium pinghaense ARSEF 439
LC128478.1 Metarhizium pinghaense ARSEF 8760
LC709293.1:1-981 Metarhizium pinghaense ARSEF 437
LC128495.1:1-981 Metarhizium pinghaense Okn3-1
LC128475.1:1-981 Metarhizium pinghaense ARSEF 445
LC128474.1:1-981 Metarhizium pinghaense ARSEF 443
KX342460.1:1-1011 Metarhizium pinghaense ARSEF 436
LC128477.1:1-991 Metarhizium pinghaense ARSEF 8736
MG384250.1:105-1124 Metarhizium pinghaense JLC-118
MG384244.1:112-1131 Metarhizium pinghaense QLC-112
MG384267.1:112-1131 Metarhizium pinghaense XBSC-135
KJ879502.1:92-1112 Metarhizium pinghaense GKVK 02 25
MG384169.1:103-1123 Metarhizium pinghaense JZF-037
LC128482.1:1-991 Metarhizium pinghaense Kgs11-1
LC128480.1:1-991 Metarhizium pinghaense Fkk5-2
KX342466.1:1-1021 Metarhizium pinghaense ARSEF 538
KP710917.1:77-1097 Metarhizium pinghaense RCEF6240
KP710908.1:100-1120 Metarhizium pinghaense RCEF6283
KP706776.1:106-1126 Metarhizium pinghaense RCEF6274
KP706746.1:83-1103 Metarhizium anisopliae RCEF5064
KP706733.1:106-1126 Metarhizium pinghaense RCEF6290
KP706732.1:106-1126 Metarhizium pinghaense RCEF6295
KP698887.1:90-1110 Metarhizium pinghaense RCEF0858
KP698877.1:69-1089 Metarhizium pinghaense RCEF6239
KP698875.1:101-1121 Metarhizium pinghaense RCEF4162
KJ879512.1:89-1109 Metarhizium pinghaense GKVK 02 37
KJ879504.1:91-1111 Metarhizium pinghaense GKVK 02 27
KJ879503.1:92-1112 Metarhizium pinghaense GKVK 02 26
KC164559.1:79-1099 Metarhizium pinghaense TYPE CBS 257.90
Metarhizium robertsii TYPE ARSEF 2575
Metarhizium robertsii ARSEF 8070
Metarhizium robertsii ARSEF 8685
Metarhizium robertsii ARSEF 7501
Metarhizium anisopliae ARSEF 6347
Metarhizium anisopliae ARSEF 798
Metarhizium anisopliae ARSEF 1080
Metarhizium humberi ESALQ 1638
Metarhizium anisopliae TYPE ARSEF 7487
Metarhizium anisopliae ARSEF 7450
Metarhizium brunneum ARSEF 1278
Metarhizium brunneum ARSEF 1066
Metarhizium brunneum ARSEF 3826
Metarhizium brunneum ARSEF 4152
Metarhizium brunneum ARSEF 3295
Metarhizium brunneum TYPE ARSEF 2107
Metarhizium guizhouense TYPE CBS 258.90
Metarhizium majus TYPE ARSEF 1914
0.01

Supplementary Figure S5. Neighbor-Joining (NJ) phylogeny of *Metarhizium* fungi as reconstructed from MzIGS3 (DUF895) marker sequences. Terminal branches are labelled by genus, species and strain designations; "TYPE" denotes the nomenclatural type strain of a species. Accession numbers are indicated for reference sequences identified in similarity searches across the GenBank database. Numbers on branches indicate bootstrap support percentages. The size bar corresponds to 1 % sequence divergence. The orthologous sequences from the *M. majus* and *M. guizhouense* type strains have been used as outgroup.
