# Supplementary material for: Metarhizium caribense sp. nov., a Novel Species of Entomopathogenic Metarhizium Fungi Associated with Weevils Impairing Coffee, Sugar Cane and Sweet Potato Cultivation
Source: J Fungi (Basel). 2024 Aug 28;10(9):612. doi: 10.3390/jof10090612 (PMC11433170; doi:10.3390/jof10090612)
Supplement: Supplementary file 1 [file jof-10-00612-s001.zip › Suppl Figure S6.pdf]

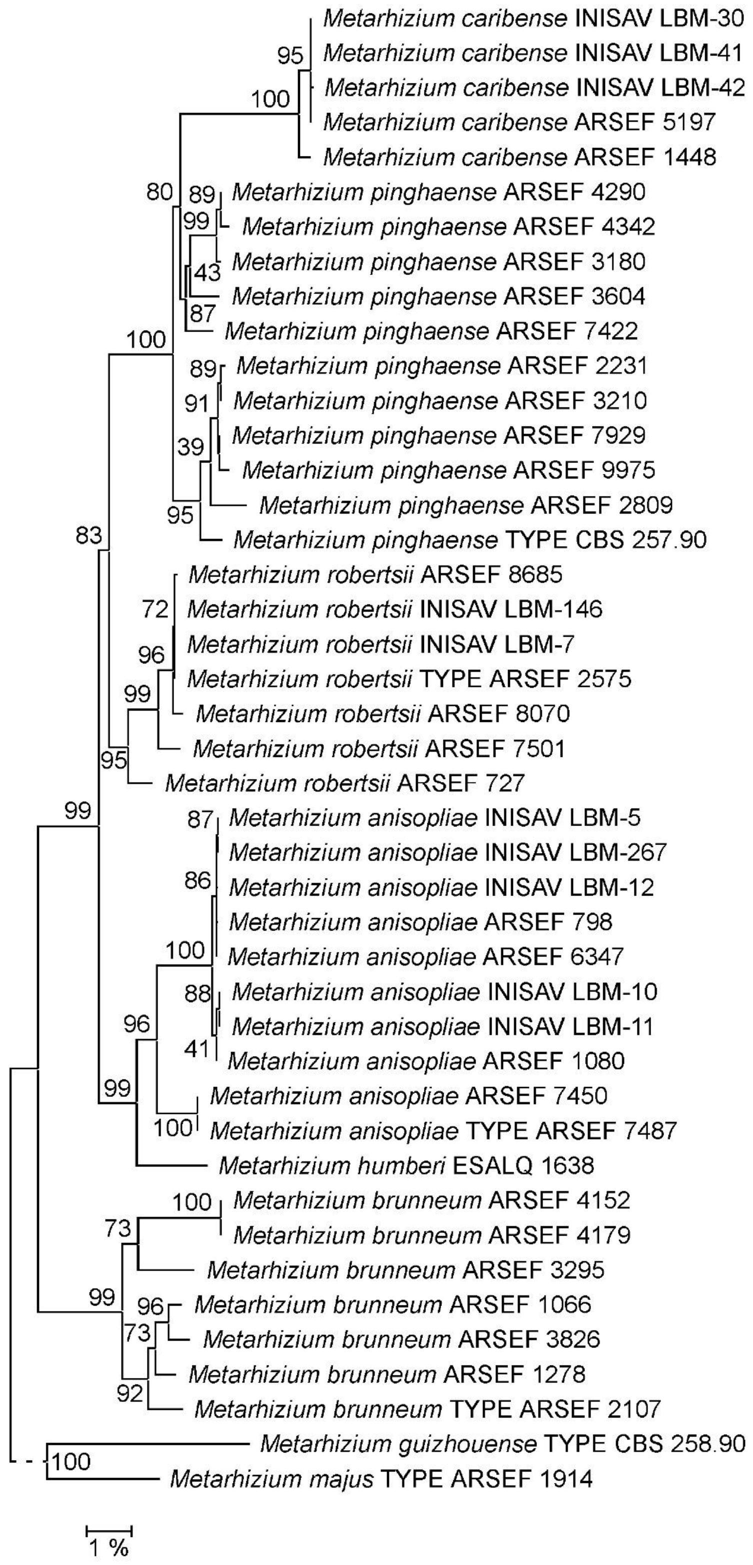

Supplementary Figure S6. Maximum Likelihood (ML) phylogeny of *Metarhizium* fungi as reconstructed from a concatenation of 5TEF, DUF895 (*MzIGS3*) and rIGS-ID800 marker sequences. Terminal branches are labelled by genus, species and strain designations; "TYPE" denotes the nomenclatural type strain of a species. Numbers on branches indicate bootstrap support percentages. The size bar corresponds to 1 % sequence divergence; branches drawn as dashed lines are not to scale. Concatenations of the orthologous sequences from the *M. majus* and *M. guizhouense* type strains have been used as outgroup.
