# Supplementary material for: Metarhizium caribense sp. nov., a Novel Species of Entomopathogenic Metarhizium Fungi Associated with Weevils Impairing Coffee, Sugar Cane and Sweet Potato Cultivation
Source: J Fungi (Basel). 2024 Aug 28;10(9):612. doi: 10.3390/jof10090612 (PMC11433170; doi:10.3390/jof10090612)
Supplement: Supplementary file 1 [file jof-10-00612-s001.zip › Suppl Figure S7.pdf]

Supplementary Figure S7. Alignment of ribosomal intergenic spacer (rIGS) sequences used for design of species-discriminating primers.

```
mcar-IDF1                              ---------- ---------- ---------- ---------- ---------- ---------- ---------- ---------- ---------- ----------   100
mcar-IDR1                              ---------- ---------- ---------- ---------- ---------- ---------- ---------- ---------- ---------- ----------   100
Metarhizium_caribense_INISAV_LBM-30    GCGGGGCGGG TGTTGGGGTT AAAGC----C ACCAAGTTA- GTGGTTGCA- ---GGCCTGA GGAGAG--CC GCCGAG---T AGT-GGTTCT AGAGGGGAAA   100
Metarhizium_caribense_INISAV_LBM-41    GCGGGGCGGG TGTTGGGGTT AAAGC----C ACCAAGTTA- GTGGTTGCA- ---GGCCTGA GGAGAG--CC GCCGAG---T AGT-GGTTCT AGAGGGGAAA   100
Metarhizium_caribense_INISAV_LBM-42    GCGGGGCGGG TGTTGGGGTT AAAGC----C ACCAAGTTA- GTGGTTGCA- ---GGCCTGA GGAGAG--CC GCCGAG---T AGT-GGTTCT AGAGGGGAAA   100
Metarhizium_caribense_ARSEF_1448       GCGGGGCGGG TGTTGGGGTT AAAGCAAGCC ACCAAGTTA- GTGGTTGCA- ---GGCTCGA GGAGAG--CC GCCGAG---T AGT-GGTTCT AGAGGGGAAA   100
Metarhizium_caribense_ARSEF_5197       GCGGGGCGGG TGTTGGGGTT AAAGC----C ACCAAGTTA- GTGGTTGCA- ---GGCCTGA GGAGAG--CC GCCGAG---T AGT-GGTTCT AGAGGGGAAA   100

Metarhizium_pingshaense_TYPE_CBS_257.90 GCGGGGCGGG TGTTGGGGTT AAAGC----C ACCAAAGTA- GTGGTTGCA- ---GGCTTGA GGAGAG--CC GCCGAGCGGT GGTTGGTTCT AGAGGGGAAA   100
Metarhizium_pingshaense_ARSEF_2231     GCGGGGCGGG TGTTGGGGTT AAAGC----C GCCAAAGTA- GTGGTTGCA- ---GGCTTGA GGAGAG--CC GCCGAGCGGT GGTTGGTTCT AGAGGGGAAA   100
Metarhizium_pingshaense_ARSEF_2809     GCGGGGCGGG TGTTGGGGTT AAAGC----C ACCGA-GTA- GTGGTTGCA- ---GGCTTGA GGAGAGAGCT GCCGAGCGGT ----GGTTCT AGAGGGGAAA   100
Metarhizium_pingshaense_ARSEF_3180     GCGGGGCGGG TGTTGGGGTT AAAGC----C ACCAA-GTA- GTGGTTGCA- ---GGCTTGA GGAGAG--CC GCCGAG---T GGT-GGTTCT AGAGGGAAAA   100
Metarhizium_pingshaense_ARSEF_3210     GCGGGGCGGG TGTTGGGGTT AAAGC----C GCCAAAGTA- GTGGTTGCA- ---GGCTTGA GGAGAG--CC GCCGAGCGGT GGTTGGTTCT AGAGGGGAAA   100
Metarhizium_pingshaense_ARSEF_3604     GCGGGGCGGG TGTTGGGGTT AAAGC----C ACCAA-GTA- GTGGCTGCA- ---GGCTTGA GGAGAG--CC GCCGAG---T GGT-GGTTCT AGAGGGGAAA   100
Metarhizium_pingshaense_ARSEF_4290     GCGGGGCGGG TGTTGGGGTT AAAGC----C ACCAA-GTA- GTGGTTGCA- ---GGCTTGA GGAGAG--CC GCCGAG---T GGT-GGTTCT AGAGGGAAAA   100
Metarhizium_pingshaense_ARSEF_4342     GCGGGGCGGG TGTTGGGGTT AAAGC----C ACCAA-GTA- GTGGTTGCA- ---GGCTTGA GGAGAG--CC GCCGAG---T GGT-GGTTCT AGAGGGAAAA   100
Metarhizium_pingshaense_ARSEF_7422     GCGGGGCGGG TGTTGGGGTT AAAGC----C ACCAA-GTAA GTGGCTGCA- ---GGCTTGA GGAGAG--CC GCCGGGCGG- ---TGGTTCT AGAGGGGAAA   100
Metarhizium_pingshaense_ARSEF_7929     GCGGGGCGGG TGTTGGGGTT AAAGC----C GCCAAAGTA- GTGGTTGCA- ---GGCTTGA GGAGAG--CC GCCGAGCGGT GGTTGGTTCT AGAGGGGAAA   100
Metarhizium_pingshaense_ARSEF_9975     GCGGGGCGGG TGTTGGGGTT AAAGC----C ACCAAAGTA- GTGGTTGCA- ---GGCTTGA GGAGAG--CC GCCGAGCGGT GGTTGGTTCT AGAGGGGAAA   100

Metarhizium_robertsii_TYPE_ARSEF_2575  GCGGGGCGGG TGTTGGGGTT AATGC----C ACCAA-GTA- GTGGTTGCA- ---GGCTTGA GGAGAG--CC GCCGAG---T GGT-GGTTCT AGAGGGAAAA   100
Metarhizium_robertsii_ARSEF_727        GCGGGGCGGG TGTTGGGGTT AATGC----C ACCAA-GTA- GTGGTTGCA- ---GGCTTGA GGAGAG--CC GCCGAG---T GGT-GGTTCT AGAGGGAAAA   100
Metarhizium_robertsii_ARSEF_7501       GCGGGGCGGG TGTTGGGGTT AATGC----C ACCAA-GTA- GTGGTTGCA- ---GGCTTGA GGAGAG--CC GCCGAG---T GGT-GGTTCT AGAGGGAAAA   100
Metarhizium_robertsii_ARSEF_8070       GCGGGGCGGG TGTTGGGGTT AATGC----C ACCAA-GTA- GTGGTTGCA- ---GGCTTGA GGAGAG--CC GCCGAG---T GGT-GGTTCT AGAGGGAAAA   100
Metarhizium_robertsii_ARSEF_8685       GCGGGGCTGG TGTTGGGGTT AATGC----C ACCAA-GTA- GTGGTTGCA- ---GGCTTGA GGAGAG--CC GCCGAG---T GGT-GGTTCT AGAGGGAAAA   100

Metarhizium_anisopliae_TYPE_ARSEF_7487 GCGGGGCGGG TGTTGGGGTT AAAGC----C ACCAA-GTA- GTGGTTACA- ---GGCTTGA GGGGAG--CT GCCGAG---T GGT-GGTTCT AGAGGGAAAA   100
Metarhizium_anisopliae_ARSEF_798       GCGGGGCGGG TGTTGGGGTT AGAGC----C ACCAA-GTA- GTGGTTACA- ---GGCTTGA GGAGAG--CT GCCGAG---T GGT-GGTTCT AGAGGGAAAA   100
Metarhizium_anisopliae_ARSEF_1080      GCGGGGCGGG TGTTGGGGTT AGAGC----C ACCAA-GTA- GTGGTTACA- ---GGCTTGA GGAGAG--CT GCCGAG---T GGT-GGTTCT AGAGGGAAAA   100
Metarhizium_anisopliae_ARSEF_6347      GCGGGGCGGG TGTTGGGGTT AGAGC----C ACCAA-GTA- GTGGTTACA- ---GGCTTGA GGAGAG--CT GCCGAG---T GGT-GGTTCT AGAGGGAAAA   100
Metarhizium_anisopliae_ARSEF_7450      GCGGGGCGGG TGTTGGGGTT AAAGC----C ACCAA-GTA- GTGGTTACA- ---GGCTTGA GGGGAG--CT GCCGAG---T GGT-GGTTCT AGAGGGAAAA   100

Metarhizium_humberi_ESALQ_1638         GCGGGGCGGG TGTTGGGGTT AAAGC----C ACCAA-GTA- GTGGTTACAT ACAGGCTTGA GGAGAG--CT GCCGAG---T GGT-GGTTCT AGAGGGAAAA   100

Metarhizium_brunneum_TYPE_ARSEF_2107   GCGGGGCGGG TGTTGGGGTT AGAGC----C ACCGA-GTA- GTGGCCGCA- ---GGCTGGA GGAGAG--CC GCCGAG---T GGT-GGTTC- AGGAGGGAAA   100
Metarhizium_brunneum_ARSEF_1066        GCGGGGCGGG TGTTGGGGTT AGAGC----C ACCGA-GTA- GTGGCCGCA- ---GGCTTGA GGAGAG--CC GCCGAG---T GGT-GGTTC- AGGAGGGAAA   100
Metarhizium_brunneum_ARSEF_1278        GCGGGGCGGG TGTTGGGGTT AGAGC----C ACCGA-GTA- GTGGCCGCA- ---GGCTTGA GGAGAG--CC GCCGAG---T GGT-GGTTC- AGGAGGGAAA   100
Metarhizium_brunneum_ARSEF_3295        GCGGGGCGGG TGTTGGGGTT AGAGC----C ACCGA-GTA- GTGGCCGCA- ---GGCTTGA GGAGAG--CC GCCGAG---T GGT-GGTTC- GGGAGGGAAA   100
Metarhizium_brunneum_ARSEF_3826        GCGGGGCGGG TGTTGGGGTT AGAGC----C ACCGA-GCA- GTGGCCGCA- ---GGCTTGA GGAGAG--CC GCCGAG---T GGT-GGTTC- AGGAGGGAAA   100
Metarhizium_brunneum_ARSEF_4152        GCGGGGCGGG TGTTGGGGTT AGAGC----C ACCGA-GTA- GTGGCCGCA- ---GGCTTGA GGAGAG--CC GCCGAG---T GGT-GGTTC- GGGAGGGAAA   100
Metarhizium_brunneum_ARSEF_4179        GCGGGGCGGG TGTTGGGGTT AGAGC----C ACCGA-GTA- GTGGCCGCA- ---GGCTTGA GGAGAG--CC GCCGAG---T GGT-GGTTC- GGGAGGGAAA   100
```

mcar-IDF1	----------	----------	----------	----------	----------	----------	----------	----------	----------	----------	200
mcar-IDR1	----------	----------	----------	----------	----------	----------	----------	----------	----------	----------	200
*Metarhizium_caribense*_INISAV_LBM-30	ATCCGCCAAG	TTC--AAAGG	TC-AGGCAAG	CGAAAGG-TT	TACCGAGTCG	GAAGGTTGGG	CGAGTAAAAA	ATT-TACCAA	GTCCAAACTA	CTGCCGGCAA	200
*Metarhizium_caribense*_INISAV_LBM-41	ATCCGCCAAG	TTC--AAAGG	TC-AGGCAAG	CGAAAGG-TT	TACCGAGTCG	GAAGGTTGGG	CGAGTAAAAA	ATT-TACCAA	GTCCAAACTA	CTGCCGGCAA	200
*Metarhizium_caribense*_INISAV_LBM-42	ATCCGCCAAG	TTC--AAAGG	TC-AGGCAAG	CGAAAGG-TT	TACCGAGTCG	GAAGGTTGGG	CGAGTAAAAA	ATT-TACCAA	GTCCAAACTA	CTGCCGGCAA	200
*Metarhizium_caribense*_ARSEF_1448	ATCCGCCAAG	TTC--AAAGG	TC-AGGCAAG	CGAAAGG-TT	TACCGAGTCG	GAAGGTTGGG	CGAGTAAAAA	ATT-TACCAA	GTCCAAAATA	CTGCCGGCAA	200
*Metarhizium_caribense*_ARSEF_5197	ATCCGCCAAG	TTC--AAAGG	TC-AGGCAAG	CGAAAGG-TT	TACCGAGTCG	GAAGGTTGGG	CGAGTAAAAA	ATT-TACCAA	GTCCAAACTA	CTGCCGGCAA	200
*Metarhizium_pingshaense*_TYPE_CBS_257.90	ATCTGCCAAG	TTC--AAAGG	TC-AGGCAAG	CGAAAGG-TT	TACCGAGTCG	GAAGGTTGGG	CGAGTAAAAA	ATT-TACCAA	GTCCAAAATA	CTGCCGGCAA	200
*Metarhizium_pingshaense*_ARSEF_2231	ATCTGCCAAG	TTC--AAAGG	TC-AGGCAAG	CGAAAGG-TT	TACCGAGTCG	GAAGGTTGGG	CGAGTAAAAA	ATT-TACCAA	GTCCAAAATA	CTGCCGGCAA	200
*Metarhizium_pingshaense*_ARSEF_2809	ATCTGCCAAG	TTC--AAAGG	TC-AGGCAAG	CGAAAGGTTG	TACCGAGTCG	GAAGGTTGGG	CGAGTAAAGA	ATT-TACCAA	GTCCAAAATA	CTGCCGGCAA	200
*Metarhizium_pingshaense*_ARSEF_3180	ATCTGCCAAG	TCC--AAAGG	TC-AGGCAAG	CGAAAGG-TT	CACCGAGTCG	GAAAGTCGGG	CGAGTAAAAA	ATT-TACCAA	GTCCAAAATA	CTGCCGGCAG	200
*Metarhizium_pingshaense*_ARSEF_3210	ATCTGCCAAG	TTC--AAAGG	TC-AGGCAAG	CGAAAGG-TT	TACCGAGTCG	GAAGGTTGGG	CGAGTAAAAA	ATT-TACCAA	GTCCAAAATA	CTGCCGGCAA	200
*Metarhizium_pingshaense*_ARSEF_3604	ATCTGCCAAG	TTC--AAAGG	TC-AGGCAAG	CGAAAGG-TT	TACCGGGTCG	GAAGGTTGGG	CGAGTAAAAA	ATT-TACCAA	GTCCAAAATA	CTGCCGGCAA	200
*Metarhizium_pingshaense*_ARSEF_4290	ATCTGCCAAG	TCC--AAAGG	TC-AGGCAAG	CGAAAGG-TT	CACCGAGTCG	GAAAGTCGGG	CGAGTAAAAA	ATT-TACCAA	GTCCAAAATA	CTGCCGGCAA	200
*Metarhizium_pingshaense*_ARSEF_4342	ATCTGCCAAG	TCC--AAAGG	TC-AGGCAAG	CGAAAGG-TT	CACCGAGTCG	GAAAGTCGGG	CGAGTAAAAA	ATT-TACCAA	GTCCAAAATA	CTGCCGGCAA	200
*Metarhizium_pingshaense*_ARSEF_7422	ATCTGCCAAG	TTC--AAAGG	TC-AGGCAAG	CGAAAGG-TT	TACCGAGTCG	GAAGGTTGGG	CGAGTAAAAA	ATT-TACCAA	GTCCAAAATA	CTGCCGGCAA	200
*Metarhizium_pingshaense*_ARSEF_7929	ATCTGCCAAG	TTC--AAAGG	TC-AGGCAAG	CGAAAGG-TT	TACCGAGTCG	GAAGGTTGGG	CGAGTAAAAA	ATT-TACCAA	GTCCAAAATA	CTGCCGGCAA	200
*Metarhizium_pingshaense*_ARSEF_9975	ATCTGCCAAG	TTC--AAAGG	TC-AGGCAAG	CGAAAGG-TT	TACCGAGTCG	GAAGGTTGGG	CGAGTAAAAA	ATT-TACCAA	GTCCAAAATA	CTGCCGGCAA	200
*Metarhizium_robertsii*_TYPE_ARSEF_2575	ATCTGCCAAG	TTC--AAAGG	TC-AGGCAAG	CGAAAGG-TT	TACCGAGTCG	GAAGGTTGGG	CGAGTAAAAA	AAT-TACCAA	GTCCAAAATA	CTGGCGGTAA	200
*Metarhizium_robertsii*_ARSEF_727	ATCTGCCAAG	TTC--AAAGG	TC-AGGCAAG	CGAAAGG-TT	TACCGAGTCG	GAAGGTTGGG	CGAGTAAAAA	AAT-TACCAA	GTCCAAAATA	CTGGCGGTAA	200
*Metarhizium_robertsii*_ARSEF_7501	ATCTGCCAAG	TTC--AAAGG	TC-AGGCAAG	CGAAAGG-TT	TACCGAGTCG	GAAGGTTGGG	CGAGTAAAAA	AAT-TACCAA	GTCCAAAATA	CTGGCGGTAA	200
*Metarhizium_robertsii*_ARSEF_8070	ATCTGCCAAG	TTC--AAAGG	TC-AGGCAAG	CGAAAGG-TT	TACCGAGTCG	GAAGGTTGGG	CGAGTAAAAA	AAT-TACCAA	GTCCAAAATA	CTGGCGGTAA	200
*Metarhizium_robertsii*_ARSEF_8685	ATCTGCCAAG	TTC--AAAGG	TC-AGGCAAG	CGAAAGG-TT	TACCGAGTCG	GAAGGTTGGG	CGAGTAAAAA	AAT-TACCAA	GTCCAAAATA	CTGGCGGTAA	200
*Metarhizium_anisopliae*_TYPE_ARSEF_7487	GTCTGCCAAG	TTC--AAAGG	TCCAGGCAAG	CGAAAAG-TT	TACCAAGTCG	GAAGGTTGGG	CGAGTAAAAA	AAT-TACCAA	GTCCAAAATA	TTGGCGGTAA	200
*Metarhizium_anisopliae*_ARSEF_798	GTCTGCCAGG	TTC--AAAGG	TCCAGGCAAG	CGAAAAG-TT	TACCAAGTCG	GAAGGTTGGG	CGAGTAAAAA	AAT-TACCAA	GTCCAAAATA	TTGGCGGTAA	200
*Metarhizium_anisopliae*_ARSEF_1080	GTCTGCCAGG	TTC--AAAGG	TCCAGGCAAG	CGAAAAG-TT	TACCAAGTCG	GAAGGTTGGG	CGAGTAAAAA	AAT-TACCAA	GTCCAAAATA	TTGGCGGTAA	200
*Metarhizium_anisopliae*_ARSEF_6347	GTCTGCCAGG	TTC--AAAGG	TCCAGGCAAG	CGAAAAG-TT	TACCAAGTCG	GAAGGTTGGG	CGAGTAAAAA	AAT-TACCAA	GTCCAAAATA	TTGGCGGTAA	200
*Metarhizium_anisopliae*_ARSEF_7450	GTCTGCCAAG	TTC--AAAGG	TCCAGGCAAG	CGAAAAG-TT	TACCAAGTCG	GAAGGTTGGG	CGAGTAAAAA	AAT-TACCAA	GTCCAAAATA	TTGGCGGTAA	200
*Metarhizium_humberi*_ESALQ_1638	ATCTGCCAAG	TTC--AAAGG	TCCAGGCAAG	CGAAAAG-TT	TACCAAGTCG	GAAGGCTGGG	CGAGTAAAAA	AAT-TACCGA	GTCCAAAATA	TTGGCGGTAA	200
*Metarhizium_brunneum*_TYPE_ARSEF_2107	ATCTGCCAAG	CTC--AAAGG	TC-AGGCAAG	CGAGAGG-TT	TACCGAGTCG	GAAAGTCGGG	CGAGTAAAAA	GATATACCAA	GTCCAAAATA	CTGCCGGCAA	200
*Metarhizium_brunneum*_ARSEF_1066	ATCTGCCAAG	CTCTCAAAGG	TC-AGGCAAG	CGAGAGG-TT	TACCGAGTCG	GAAAGTCGGG	CGAGTAAAAA	GATATACCAA	GTCCAAAATA	CTGCCGGCAA	200
*Metarhizium_brunneum*_ARSEF_1278	ATCTGCCAAG	CTC--AAAGG	TC-AGGCAAG	CGAGAGGTTT	TACCGAGTCG	GAAAGTCGGG	CGAGTAAAAA	GATATACCAA	GTCCAAAATA	CTGCCGGCAA	200
*Metarhizium_brunneum*_ARSEF_3295	ATCTGCCAAG	CTC--AAAGG	TC-AGGCAAG	CGAGAGG-TT	TACCGGGTCG	GAAAGTCGGG	CGAGTAAAAA	GATATACCAA	GTCCAAAACA	CTGCCGGCAA	200
*Metarhizium_brunneum*_ARSEF_3826	ATCTGCCAAG	CTC--AAAGG	TC-AGGCAAG	CGAGAGG-TT	TACCGAGTCG	GAAAGTCGGG	CGAGTAAAAA	GATATACCAA	GTCCAAAATA	CTGCCGGCAA	200
*Metarhizium_brunneum*_ARSEF_4152	ATCTGCCAAG	CTC--AAAGG	TC-AGGCAAG	CGAGAGG-TT	TACCGGGTCG	GAAAGTCGGG	CGAGTAAAAA	GATATACCAA	GTCCAAAATA	CTGCCGGCAA	200
*Metarhizium_brunneum*_ARSEF_4179	ATCTGCCAAG	CTC--AAAGG	TC-AGGCAAG	CGAGAGG-TT	TACCGGGTCG	GAAAGTCGGG	CGAGTAAAAA	GATATACCAA	GTCCAAAATA	CTGCCGGCAA	200

```
mcar-IDF1                                  ---------- ---------- ---------- ---------- ---------- ---------- ---------- ---------- ---------- ----------   300
mcar-IDR1                                  ---------- ---------- ---------- ---------- ---------- ---------- ---------- ---------- ---------- ----------   300
Metarhizium_caribense_INISAV_LBM-30        CATACCCGGG ACTCCGAGTA AGTCGGGGAA GAAGTTGGCG GATTTTTTCC CAACCAACTC AACTTTTGAC CAATTT-ACC TGCCCGAGCT CACGGGTAGG   300
Metarhizium_caribense_INISAV_LBM-41        CATACCCGGG ACTCCGAGTA AGTCGGGGAA GAAGTTGGCG GATTTTTTCC CAACCAACTC AACTTTTGAC CAATTT-ACC TGCCCGAGCT CACGGGTAGG   300
Metarhizium_caribense_INISAV_LBM-42        CATACCCGGG ACTCCGAGTA AGTCGGGGAA GAAGTTGGCG GATTTTTTCC CAACCAACTC AACTTTTGAC CAATTT-ACC TGCCCGAGCT CACGGGTAGG   300
Metarhizium_caribense_ARSEF_1448           CATACCCGGG ACTCCGAGTA AGTCGGGGAA GAAGTTGGCG GATTTTTCCC CAACCAACTC AACTTTTGAC CAATTT-ACC TGCCCGAGCT CACGGGTAGG   300
Metarhizium_caribense_ARSEF_5197           CATACCCGGG ACTCCGAGTA AGTCGGGGAA GAAGTTGGCG GATTTTTTCC CAACCAACTC AACTTTTGAC CAATTT-ACC TGCCCGAGCT CACGGGTAGG   300

Metarhizium_pingshaense_TYPE_CBS_257.90    CATACCCGGG ACTCCGAGTA AGTCGGGGAA GAAGTTGGCG GATTTTTTCC CAACCAACTC AACTTTTGAC CAATTT-ACC TGCCCGAGCT CACGGGTAGG   300
Metarhizium_pingshaense_ARSEF_2231         CATACCCGGG ACTCCGAGTA AGTCGGGGAA GAAGTTGGCG GATTTTTTCC CAACCAACTC AACTTTTGAC CAATTT-ACC TGCCCGAGCT CACGGGTAGG   300
Metarhizium_pingshaense_ARSEF_2809         CATACCCGGG ACTCCGAGTA AGTCGGGGAA GAAGTTGGCG GATTTTTTCC CAACCAACTC AACTTTTGAC CAATTT-ACC TGCCCGAGCT CACGGGTAGG   300
Metarhizium_pingshaense_ARSEF_3180         CATACCCGGG ACTCCGAGTA AGCCGGGGAA GAAGTTGGCG GATTTTTTCC CAACCAACTC AACTT--GAC CAATTT-ACC TGCCCGAGCT CACGGGTAGG   300
Metarhizium_pingshaense_ARSEF_3210         CATACCCGGG ACTCCGAGTA AGTCGGGGAA GAAGTTGGCG GATTTTTTCC CAACCAACTC AACTTTTGAC CAATTT-ACC TGCCCGAGCT CACGGGTAGG   300
Metarhizium_pingshaense_ARSEF_3604         CATACCCGGG ACTCCGAGTA AGTCGGGGAA GAAGTTGGCG GATTTTTCCC CAACCAACTC AACTTTTGAC CAATTT-ACC TGCCCGAGCT CACGGGTAGG   300
Metarhizium_pingshaense_ARSEF_4290         CATACCCGGG ACTCCGAGTA AGTCGGGGAA GAAGTTGGCG GATTTTTTCC CAACCAACTC AACTT--GAC CAATTT-ACC TGCCCGAGCT CACGGGTAGG   300
Metarhizium_pingshaense_ARSEF_4342         CATACCCGGG ACTCCGAGTA AGTCGGGGAA GAAGTTGGCG GATTTTTTCC CAACCAACTC AACTT--GAC CAATTT-ACC TGCCCGAGCT CACGGGTAGG   300
Metarhizium_pingshaense_ARSEF_7422         CATACCCGGG ACTCCGAGTA AGTCGGGGAA GAAGTTGGCG GATTTTTTCC CAACCAACTC AACTTTTGAC CAATTT-ACC TGCCCGAGCT CACGGGTAGG   300
Metarhizium_pingshaense_ARSEF_7929         CATACCCGGG ACTCCGAGTA AGTCGGGGAA GAAGTTGGCG GATTTTTTCC CAACCAACTC AACTTTTGAC CAATTT-ACC TGCCCGAGCT CACGGGTAGG   300
Metarhizium_pingshaense_ARSEF_9975         CATACCCGGG ACTCCGAGTA AGTCGGGGAA GAAGTTGGCG GATTTTTTCC CAACCAACTC AACTTTTGAC CAATTT-ACC TGCCCGAGCT CACGGGTAGG   300

Metarhizium_robertsii_TYPE_ARSEF_2575      CCTACCCGGG ACTCCGAGTA AGTCGGGGAA GAAGTTGGCG GATTTTTTCC CAACCAACTC AACTTTTGAC CAATTT-ACC TGCCCGAGCT CACGGGTAGG   300
Metarhizium_robertsii_ARSEF_727            CCTACCCGGG ACTCCGAGTA AGTCGGGGAA GAAGTTGGCG GATTTTTTCC CAACCAACTC AACTTTTGAC CAATTT-ACC TGCCCGAGCT CACGGGTAGG   300
Metarhizium_robertsii_ARSEF_7501           CCTACCCGGG ACTCCGAGTA AGTCGGGGAA GAAGTTGGCG GATTTTTCCC CAACCAACTC AACTTTTGAC CAATTT-ACC TGCCCGAGCT CACGGGTAGG   300
Metarhizium_robertsii_ARSEF_8070           CCTACCCGGG ACTCCGAGTA AGTCGGGGAA GAAGTTGGCG GATTTTTTCC CAACCAACTC AACTTTTGAC CAATTT-ACC TGCCCGAGCT CACGGGTAGG   300
Metarhizium_robertsii_ARSEF_8685           CCTACCCGGG ACTCCGAGTA AGTCGGGGAA GAAGTTGGCG GATTTTTTCC CAACCAACTC AACTTTTGAC CAATTT-ACC TGCCCGAGCT CACGGGTAGG   300

Metarhizium_anisopliae_TYPE_ARSEF_7487     CCTACCCGGG ACTCCGAGTA AGTCGGGGAA AAAGTTGGCG GATTTTTTCC CAACCAACTC AACTTTTGAC CAATTT-ACC TGCCCGAGCT CACGGGTAGG   300
Metarhizium_anisopliae_ARSEF_798           CCTACCCGGG ACTCCGAGTA AGTCGGGGAA AAAGTTGGCG GATTTTTCCC CAACCAACTC AACTTTTGAC CAATTT-ACC TGCCCGAGCT CACGGGTAGG   300
Metarhizium_anisopliae_ARSEF_1080          CCTACCCGGG ACTCCGAGTA AGTCGGGGAA AAAGTTGGCG GATTTTTCCC CAACCAACTC AACTTTTGAC CAATTT-ACC TGCCCGAGCT CACGGGTAGG   300
Metarhizium_anisopliae_ARSEF_6347          CCTACCCGGG ACTCCGAGTA AGTCGGGGAA AAAGTTGGCG GATTTTTCCC CAACCAACTC AACTTTTGAC CAATTT-ACC TGCCCGAGCT CACGGGTAGG   300
Metarhizium_anisopliae_ARSEF_7450          CCTACCCGGG ACTCCGAGTA AGTCGGGGAA AAAGTTGGCG GATTTTTTCC CAACCAACTC AACTTTTGAC CAATTT-ACC TGCCCGAGCT CACGGGTAGG   300

Metarhizium_humberi_ESALQ_1638             CCTACCCGGG ACTCCGAGTA AGTCGGGGAA GAAGTTGGCG GATTTTTTCC CAACCAACTC AACTTTTGAC CAATTT-ACC TGCCCGAGCT CACGGGTAGG   300

Metarhizium_brunneum_TYPE_ARSEF_2107       CATACCCGGG ACCCCGAGTA AGTCGGGGAA GAAGTTGGCG GATGTTTTTC CAGCCAACTC AACTTT-GAC CAATTT-ACC TGCCCGAGCT CACGGGTAGG   300
Metarhizium_brunneum_ARSEF_1066            CATACCCGGG ACCCCGAGTA AGTCGGGGAA GAAGTTGGCG GGTGTTTTTC CAGCCAACTC AACTTT-GAC CAATTTTACC TGCCCGAGCT CACGGGTAGG   300
Metarhizium_brunneum_ARSEF_1278            CATACCCGGG ACCCCGAGTA AGTCGGGGAA GAAGTTGGCG GGTGTTTTCC CAGCCAACTC AACTCT-GAC CAATTT-ACC TGCCCGAGCT CACGGGTAGG   300
Metarhizium_brunneum_ARSEF_3295            CATACCCGGG ACCCCGAGTA AGTCGGGGAA GAAGTTGGCG GATGTTTTTC CAGCCAACTC AACTTT-GAC CAATTT-ACC TGCCCGAGCT CACGGGTAGG   300
Metarhizium_brunneum_ARSEF_3826            CATACCCGGG ACCCCGAGTA AGTCGGGGAA GAAGTTGGCG GATGTTTTTC CAGCCAACTC AACTTT-GAC CAATTT-ACC TGCCCGAGCT CACGGGTAGG   300
Metarhizium_brunneum_ARSEF_4152            CATACCCGGG ACCCCGAGTA AGTCGGGGAA GAAGTTGGCG GATGTTTTCC CAGCCAACTC AACTTT-GAC CAATTT-ACC TGCCCGAGCT CACGGGTAGG   300
Metarhizium_brunneum_ARSEF_4179            CATACCCGGG ACCCCGAGTA AGTCGGGGAA GAAGTTGGCG GATGTTTTCC CAGCCAACTC AACTTT-GAC CAATTT-ACC TGCCCGAGCT CACGGGTAGG   300
```

mcar-IDF1	---------- ---------- ---------- ---------- ---------- ---------- ---------- ---------- ---------- ----------	400
mcar-IDR1	---------- ---------- ---------- ---------- ---------- ---------- ---------- ---------- ---------- ----------	400
*Metarhizium_caribense*_INISAV_LBM-30	CTGTGGTTTA TTTTTGGACT TT-GTAGATT TACTTGAACC AATTTACAGG AAGTTGCCTG CCGAATTACC TGGGCTCCCG GGTAGGCTAT AGTTAACTTT	400
*Metarhizium_caribense*_INISAV_LBM-41	CTGTGGTTTA TTTTTGGACT TT-GTAGATT TACTTGAACC AATTTACAGG AAGTTGCCTG CCGAATTACC TGGGCTCCCG GGTAGGCTAT AGTTAACTTT	400
*Metarhizium_caribense*_INISAV_LBM-42	CTGTGGTTTA TTTTTGGACT TT-GTAGATT TACTTGAACC AATTTACAGG AAGTTGCCTG CCGAATTACC TGGGCTCCCG GGTAGGCTAT AGTTAACTTT	400
*Metarhizium_caribense*_ARSEF_1448	CTGTGGTTTA TTTTTGGACT TT-GTAGATT TACTTAAATC AATTTACAGG AAGTTGCCTG CCGAATTACC TGGGCTCCCG GGTAGGCTAT AGTTAACTTT	400
*Metarhizium_caribense*_ARSEF_5197	CTGTGGTTTA TTTTTGGACT TT-GTAGATT TACTTGAACC AATTTACAGG AAGTTGCCTG CCGAATTACC TGGGCTCCCG GGTAGGCTAT AGTTAACTTT	400
*Metarhizium_pingshaense*_TYPE_CBS_257.90	CTGCGGTTTA TTTTTGGACT TT-GTAGATT TGCTTGAATC AATTTACAGG AAGTTGCCTG CCGAATTACC TGGGCTCCCG GGTAGGCTAT AGTTAACTTT	400
*Metarhizium_pingshaense*_ARSEF_2231	CTGCGGTTTA TTTTTGGACT TT-GTAGATT TGCTTGAATC AATTTACAGG AAGTTGCCTG CCGAATTACC TGGGCTCCCG GGTAGGCTAT AGTTAACTTT	400
*Metarhizium_pingshaense*_ARSEF_2809	CTGCGGTTTA TTTTTGGACT TT-GTAGATT TGCTTGAATC AATTTACAGG AAGTTGCCTG CCGAATTACC TTGGCTCCCG GGTAGGCTAT AGTTAACTTT	400
*Metarhizium_pingshaense*_ARSEF_3180	CTGCGGTCAA TTTTTGGACT TT-GTAGATT TACTTGAATC AATTCACAAG AAGTTACCTG CCGAATTACC TGGGCTCCCG GGTAGGCTAT AGTCAACTTT	400
*Metarhizium_pingshaense*_ARSEF_3210	CTGCGGTTTA TTTTTGGACT TT-GTAGATT TGCTTGAATC AATTTACAGG AAGTTGCCTG CCGAATTACC TGGGCTCCCG GGTAGGCTAT AGTTAACTTT	400
*Metarhizium_pingshaense*_ARSEF_3604	CTGCGGTTTA TTTTCGGACT TT-GTAGATT TACTTGAATC AATTCACAGG AAGTTATCTG CCGAATTACC TGGGCTCCCG GGTAGG---- ----------	400
*Metarhizium_pingshaense*_ARSEF_4290	CTGCGGTCAA TTTTTGGACT TT-GTAGATT TACTTGAATC AATTCACAAG AAGTTACCTG CCGAATTACC TGGGCTCCCG GGTAGGCTAT AGTCAACTTT	400
*Metarhizium_pingshaense*_ARSEF_4342	CTGCGGTCAA TTTTTGGACT TT-GTAGATT TACTTGAATC AATTCACAAG AAGTTACCTG CCGAATTACC TGGGCTCCCG GGTAGGCTAT AGTCAACTTT	400
*Metarhizium_pingshaense*_ARSEF_7422	CTGCGGTTTA TTTTTGGACT TT-GTAGATT TACTTAAATC AATTTACAGC AAGTTGCCTG CCGAATTACC TGGGCTCCCG GGTAGGCTAT AGT-AACTTT	400
*Metarhizium_pingshaense*_ARSEF_7929	CTGCGGTTTA TTTTTGGACT TT-GTAGATT TGCTTGAATC AATTTACAGG AAGTTGCCTG CCGAATTACC TGGGCTCCCG GGTAGGCTAT AGTTAACTTT	400
*Metarhizium_pingshaense*_ARSEF_9975	CTGCGGTTTA TTTTTGGACT TT-GTAGATT TGCTTGAATC AATTTACAGG AAGTTGCCTG CCGAATTACC TGGGCTCCCG GGTAGGCTAT AGTTAACTTT	400
*Metarhizium_robertsii*_TYPE_ARSEF_2575	CTGCGGTTTA TTTTTGGACT TT-GTAGATT TACTTAAATC AATTCACAGG AAGTTACCTG CCGAATTACC TGGGCTCCCG GGTAGGCTAT AGTTAACTTT	400
*Metarhizium_robertsii*_ARSEF_727	CTGCGGTTTA TTTTTGGACT TT-GTAGATT TACTTAAATC AATTCACAGG AAGTTACCTG CCGAATTACC TGGGCTCCCG GGTAGGCTAT AGTTAACTTT	400
*Metarhizium_robertsii*_ARSEF_7501	CTGCGGTTTA TTTTTGGACT TT-GTAGATT TACTTAAATC AATTCACAGG AAGTTACCTG CCGAATTACC TGGGCTCCCG GGTAGGCTAT AGTTAACTTT	400
*Metarhizium_robertsii*_ARSEF_8070	CTGCGGTTTA TTTTTGGACT TT-GTAGATT TACTTAAATC AATTCACAGG AAGTTACCTG CCGAATTACC TGGGCTCCCG GGTAGGCTAT AGTTAACTTT	400
*Metarhizium_robertsii*_ARSEF_8685	CTGCGGTTTA TTTTTGGACT TT-GTAGATT TACTTAAATC AATTCACAGG AAGTTACCTG CCGAATTACC TGGGCTCCCG GGTAGGCTAT AGTTAACTTT	400
*Metarhizium_anisopliae*_TYPE_ARSEF_7487	CTGCGGTTTA TTTTTGGACT TT-GTAGATT TACTTGAATC AATTCACGGG AAGTTACCTG CCGAATTACC TGGGCTCCCG GGTAGGCTAT AGTTAACTTT	400
*Metarhizium_anisopliae*_ARSEF_798	CTGCGGTTTA TTTTTGGACT TT-GTAGATT TACTTGAATC AATTCACGGG AAGTTACCTG CCGAATTACC TGGGCTCCCG GGTAGGCTAT AGTCAACTTT	400
*Metarhizium_anisopliae*_ARSEF_1080	CTGCGGTTTA TTTTTGGACT TT-GTAGATT TACTTGAATC AATTCACGGG AAGTTACCTG CCGAATTACC TGGGCTCCCG GGTAGGCTAT AGTCAACTTT	400
*Metarhizium_anisopliae*_ARSEF_6347	CTGCGGTTTA TTTTTGGACT TT-GTAGATT TACTTGAATC AATTCACGGG AAGTTACCTG CCGAATTACC TGGGCTCCCG GGTAGGCTAT AGTCAACTTT	400
*Metarhizium_anisopliae*_ARSEF_7450	CTGCGGTTTA TTTTTGGACT TT-GTAGATT TACTTGAATC AATTCACGGG AAGTTACCTG CCGAATTACC TGGGCTCCCG GGTAGGCTAT AGTTAACTTT	400
*Metarhizium_humberi*_ESALQ_1638	CTGC------ ---------- -------ATT TACTTGAATC AATTCACAGG AAGTTACCTG CCGAATTACC TGGGCTCCCG GGTAGGCTGT AGTTCACTTT	400
*Metarhizium_brunneum*_TYPE_ARSEF_2107	CTGCGGTTAA TTTTTGGACT TTTGCGGATT TACTTGAATC AATTCACAGG AAGTTACCTG CCAAATTGCC TGGGCTCCCG GGTAGGCTAT AGTTGACTTT	400
*Metarhizium_brunneum*_ARSEF_1066	CTGCGGTTAA TTTTTGGACT TT-GCGGATT TACTTGAATC AATTCACAGG AAGTTACCTG CCAAATTGCC TGGGCTCCCG GGTAGGCTAT AGTTGACTTT	400
*Metarhizium_brunneum*_ARSEF_1278	CTGCGGTTAA TTTTTGGACT TT-GCGGATT TACTTGAATC AATTCACAGG AGGTTACCTG CCAAATTGCC TGGGCTCCCG GGTAGGCTAT AGTTGACTTT	400
*Metarhizium_brunneum*_ARSEF_3295	CTGCGGTTAA TTTTTGGACT TT-GCGGGTT CACTCGAATC AATTCACAGG AAGTTACCTG CCAAATTGCC TGGGCTCCCG GGTAGGCTAT AGTTGACTTT	400
*Metarhizium_brunneum*_ARSEF_3826	CTGCGGTTAA TTTTTGGACT TT-GCGGATT TACTTGAATC AATTCACAGG AAGTTACCTG CCAAATTGCC TGGGCTCCCG GGTAGGCTAT AGTTGACTTT	400
*Metarhizium_brunneum*_ARSEF_4152	CTGCGGTTAA TTTTTGGACT TG-GCGGATT TACTTGAATC AATCCACAGG AAGTTACCTG CCGAATTACC TGGGCTCCCG GGTAGGCCAT AGTTGACTTG	400
*Metarhizium_brunneum*_ARSEF_4179	CTGCGGTTAA TTTTTGGACT TG-GCGGATT TACTTGAATC AATCCACAGG AAGTTACCTG CCGAATTACC TGGGCTCCCG GGTAGGCCAT AGTTGACTTG	400

mcar-IDF1	---------- ---------- ---------- ---------- ---------- ---------- ---------- ---------- ---------- ----**GGACTT**	500
mcar-IDR1	---------- ---------- ---------- ---------- ---------- ---------- ---------- ---------- ---------- ----------	500
*Metarhizium_caribense*_INISAV_LBM-30	TGGACTT--- ---------- ---------- --CCGACTAT AACCTGGGAT TTTCCCAACT ATA--GCTCA CGGGTAGG-T TAGTTGTGAT TTTTGGACTT	500
*Metarhizium_caribense*_INISAV_LBM-41	TGGACTT--- ---------- ---------- --CCGACTAT AACCTGGGAT TTTCCCAACT ATA--GCTCA CGGGTAGG-T TAGTTGTGAT TTTTGGACTT	500
*Metarhizium_caribense*_INISAV_LBM-42	TGGACTT--- ---------- ---------- --CCGACTAT AACCTGGGAT TTTCCCAACT ATA--GCTCA CGGGTAGG-T TAGTTGTGAT TTTTGGACTT	500
*Metarhizium_caribense*_ARSEF_1448	TGGACTT--- ---------- ---------- --CCGACCAT AACCTGGGAT TTTCCCAACT ATA--GCTCA CGGGTAGGGT TA-TTGTGAT TTTTGGACTT	500
*Metarhizium_caribense*_ARSEF_5197	TGGACTT--- ---------- ---------- --CCGACTAT AACCTGGGAT TTTCCCAACT ATA--GCTCA CGGGTAGG-T TAGTTGTGAT TTTTGGACTT	500
*Metarhizium_pingshaense*_TYPE_CBS_257.90	TGGACTT--- ---------- ---------- --TCGACTAT AACCTGGGAT TTTCCCAACT ATA--GCTCA CGGGTAGG-T TAGTTGTGAT TTTTGGAC--	500
*Metarhizium_pingshaense*_ARSEF_2231	TGGACTT--- ---------- ---------- --TCGACTAT AACCTGGGAT TTTCCCAACT ATATAGCTCA CGGGTAGG-T TAGTTGTGAT TTTTGGACTT	500
*Metarhizium_pingshaense*_ARSEF_2809	TGGACTT--- ---------- ---------- --CCGACTAT AACCTGGGAT TTTCCCAACT ATA--GCTCA CGGGTAGG-T TAGTTGTGGT TTTTGGACTT	500
*Metarhizium_pingshaense*_ARSEF_3180	TGGACTT--- ---------- ---------- --CCGACTAT AACCTGGGAT TTTCCCAACT ATA--GCTCA CGGGTAGG-T TAGTTGTGAT TTTTGGACTT	500
*Metarhizium_pingshaense*_ARSEF_3210	TGGACTT--- ---------- ---------- --TCGACTAT AACCTGGGAT TTTCCCAACT ATATAGCTCA CGGGTAGG-T TAGTTGTGAT TTTTGGACTT	500
*Metarhizium_pingshaense*_ARSEF_3604	---------- ---------- ---------- ---------- ---------- ---------- ---------- ---------T TAGTTGTGAT TTTTGGACTT	500
*Metarhizium_pingshaense*_ARSEF_4290	TGGACTT--- ---------- ---------- --CCGACTAT AACCTGGGAT TTTCCCAACT ATA--GCTCA CGGGTAGG-T TAGTTGTGAT TTTTGGACTT	500
*Metarhizium_pingshaense*_ARSEF_4342	TGGACTT--- ---------- ---------- --CCGACTAT AACCTGGGAT TTTCCCAACT ATA--GCTCA CGGGTAGG-T TAGTTGTGAT TTTTGGACTT	500
*Metarhizium_pingshaense*_ARSEF_7422	TGGACTT--- ---------- ---------- --CCGACTAT AACCTGGGAT TTTCCCAACT ATA--GCTCA CGGGTAGG-T TAGTTGTGAT TTTTGGAGAC	500
*Metarhizium_pingshaense*_ARSEF_7929	TGGACTT--- ---------- ---------- --TCGACTAT AACCTGGGAT TTTCCCAACT ATATAGCTCA CGGGTAGG-T TAGTTGTGAT TTTTGGACTT	500
*Metarhizium_pingshaense*_ARSEF_9975	TGGACTT--- ---------- ---------- --TCGACTAT AACCTGGGAT TTTCCCAACT ATATAGCTCA CGGGTAGG-T TAGTTGTGAT TTTTGGACTT	500
*Metarhizium_robertsii*_TYPE_ARSEF_2575	TGGACTT--- ---------- ---------- --CCGACTAT AACCTGGGAT TTTCCCAACC ATA--GCTCA CGGGTAGG-T TAGTTGTGAT TTTTGGACTC	500
*Metarhizium_robertsii*_ARSEF_727	TGGACTT--- ---------- ---------- --CCGACTAT AACCTGGGAT TTTCCCAACC ATA--GCTCA CGGGTAGG-T TAGTTGTGAT TTTTGGACTC	500
*Metarhizium_robertsii*_ARSEF_7501	TGGACTT--- ---------- ---------- --CCGACTAT AACCTGGGAT TTTCCCAACC ATA--GCTCA CGGGTAGG-T TAGTTGTGAT TTTTGGACTC	500
*Metarhizium_robertsii*_ARSEF_8070	TGGACTT--- ---------- ---------- --CCGACTAT AACCTGGGAT TTTCCCAACC ATA--GCTCA CGGGTAGG-T TAGTTGTGAT TTTTGGACTC	500
*Metarhizium_robertsii*_ARSEF_8685	TGGACTT--- ---------- ---------- --CCGACTAT AACCTGGGAT TTTCCCAACC ATA--GCTCA CGGGTAGG-T TAGTTGTGAT TTTTGGACTC	500
*Metarhizium_anisopliae*_TYPE_ARSEF_7487	TGGACTT--- ---------- ---------- --GCGACTAT AACCTGGGAT TTTCCCAACC ATA--GCTCA CGGGTAGG-C TAGTTGTGAT TTTTGGACTT	500
*Metarhizium_anisopliae*_ARSEF_798	TGGACTT--- ---------- ---------- --GCGACTAT AACCTGGGAT TTTCCCAACC AGA--GCTCA CGGGTAGG-C TAGTTGTGAT TTTTGGACTT	500
*Metarhizium_anisopliae*_ARSEF_1080	TGGACTT--- ---------- ---------- --GCGACTAT AACCTGGGAT TTTCCCAACC AGA--GCTCA CGGGTAGG-C TAGTTGTGAT TTTTGGACTT	500
*Metarhizium_anisopliae*_ARSEF_6347	TGGACTT--- ---------- ---------- --GCGACTAT AACCTGGGAT TTTCCCAACC AGA--GCTCA CGGGTAGG-C TAGTTGTGAT TTTTGGACTT	500
*Metarhizium_anisopliae*_ARSEF_7450	TGGACTT--- ---------- ---------- --GCGACTAT AACCTGGGAT TTTCCCAACC ATA--GCTCA CGGGTAGG-C TAGTTGTGAT TTTTGGACTT	500
*Metarhizium_humberi*_ESALQ_1638	TAGACTT--- ---------- ---------- --GCGACTAT AACCTGGGAT TTTCCCAACC ATA--GCTCA CGGGTAGG-T TAGTTGAGAT TTTTGGACTT	500
*Metarhizium_brunneum*_TYPE_ARSEF_2107	TGGACTTGGC GGATTTTTTT AAACTTACTT CCCCGACTAT AACCTGGGAT TTTCCCGACC ATA--GCTCA CGGGTAGG-T AAGTTGTGAT TTTTGGACTT	500
*Metarhizium_brunneum*_ARSEF_1066	TGGACTTGGC GGATTTTTTT AAACTTACTT CCCCGACTAT AACCTGGGAT TTTCCCGACC ATG--GCTCA CGGGTAGG-T TAGTTGTGAT TTTTGGACTT	500
*Metarhizium_brunneum*_ARSEF_1278	TGGACACGGC GGATTTTTTT AAACTTACTT CCCCGACTAT AACCTGGGAT TTTCCCGACC ATA--GCTCA CGGGTAGG-T TAGTTGTGAT TTTTGGAC--	500
*Metarhizium_brunneum*_ARSEF_3295	TGGACTTGGC GGATTTTTTT AAACTTACTT CCCCCACTAT GACCTGGGAT TTTCCCGACC ATA--GCTCA CGGGTAGG-T TAGTTGTGAT ----------	500
*Metarhizium_brunneum*_ARSEF_3826	TGGACTTGGC GGATTTTTTT AAACTTACTT CCCCGACTAT AACCTGGGAT TTTCCCGACC ATG--GCTCA CGGGTAGG-T TAGTTGTGAT TTTTGGACTT	500
*Metarhizium_brunneum*_ARSEF_4152	TGGACTTGGC GGATTTTTTC CAACCTACTT CCCCGACTAT AACCTGGGAT TTTCCCGACC ATA--GCTCA CGGGTAGG-T TAGTTGTGAT TTTTGGACTT	500
*Metarhizium_brunneum*_ARSEF_4179	TGGACTTGGC GGATTTTTTC CAACCTACTT CCCCGACTAT AACCTGGGAT TTTCCCGACC ATA--GCTCA CGGGTAGG-T TAGTTGTGAT TTTTGGACTT	

mcar-IDF1	**GGCATATTTG CTTGAATTG**- ---------- ---------- ---------- ---------- ---------- ---------- ---------- ----------	600
mcar-IDR1	---------- ---------- ---------- ---------- ---------- ---------- ---------- ---------- ---------- ----------	600
*Metarhizium_caribense*_INISAV_LBM-30	GGCATATTTG CTTGAATTGA TTTGCAATTC GTTTCCTGCC GAGTCGGCGG AATTTACCTG CCCGACTTGC CTGGGCTCCC GGGTAGGCTA GACTTGGCAG	600
*Metarhizium_caribense*_INISAV_LBM-41	GGCATATTTG CTTGAATTGA TTTGCAATTC GTTTCCTGCC GAGTCGGCGG AATTTACCTG CCCGACTTGC CTGGGCTCCC GGGTAGGCTA GACTTGGCAG	600
*Metarhizium_caribense*_INISAV_LBM-42	GGCATATTTG CTTGAATTGA TTTGCAATTC GTTTCCTGCC GAGTCGGCGG AATTTACCTG CCCGACTTGC CTGGGCTCCC GGGTAGGCTA GACTTGGCAG	600
*Metarhizium_caribense*_ARSEF_1448	GGCATATTTG CTTGAATTGA TTTGCAATTA GTTTCCTGCC GAGTCGGCGG AATTTACCTG CCCGACTTGC CTGGGCTCCC GGGTAGGCTA GACTTGGCAG	600
*Metarhizium_caribense*_ARSEF_5197	GGCATATTTG CTTGAATTGA TTTGCAATTC GTTTCCTGCC GAGTCGGCGG AATTTACCTG CCCGACTTGC CTGGGCTCCC GGGTAGGCTA GACTTGGCAG	600
*Metarhizium_pingshaense*_TYPE_CBS_257.90	GGTGAAATTT CTAC------ ---------- -TTTCCTGCC AAGTCTAGCC TACCCGGGAG CCCAGGCAAG TCGGGCAGGT AAATTCCGCC AACTCGGCAG	600
*Metarhizium_pingshaense*_ARSEF_2231	GGTGAAATTT CTAC------ ---------- -TTTCCTGCC AAGTCTAGCC TACCCGGGAG CCCAGGCAAG TCGGGCAGGT AAATTCCGCC AACTCGGCAG	600
*Metarhizium_pingshaense*_ARSEF_2809	GGTGAAATTT CTGC------ ---------- -TTTCCTGCC AAGTCTAGCC TACCCGGGAG CCCAGGCAAG TCGGGGAGGT AAATTCCGCC AACTCGGCAG	600
*Metarhizium_pingshaense*_ARSEF_3180	GGTGAAATTT CTAC------ ---------- -TTTCCTGCC AAGTCTAGCC TACCCGGGAG CCCAGGCAAG TCGGGCAGGT AAATTCCGCC AACTCGGCAG	600
*Metarhizium_pingshaense*_ARSEF_3210	GGTGAAATTT CTAC------ ---------- -TTTCCTGCC AAGTCTAGCC TACCCGGGAG CCCAGGCAAG TCGGGCAGGT AAATTCCGCC AACTCGGCAG	600
*Metarhizium_pingshaense*_ARSEF_3604	GGTGAAATTT CTAC------ ---------- -TTTCCTGCC AAGTCTAGCC TACCCGGGAG CCCAGGCAAG TCGGGCAGGT AAATTCCGCC AACTCGGCAG	600
*Metarhizium_pingshaense*_ARSEF_4290	GGTGAAATTT CTAC------ ---------- -TTTCCTGCC AAGTCTAGCC TACCCGGGAG CCCAGGCAAG TCGGGCAGGT AAATTCCGCC AACTCGGCAG	600
*Metarhizium_pingshaense*_ARSEF_4342	GGTGAAATTT CTAC------ ---------- -TTTCCTGCC AAGTCTAGCC TACCCGGGAG CCCAGGCAAG TCGGGCAGGT AAATTCCGCC AACTCGGCAG	600
*Metarhizium_pingshaense*_ARSEF_7422	GGTGAAACTT CTAC------ ---------- -TTTCCTGCC AAGTCTAGCC TACCCGGGAG CCCAGGCAAG TCGGGCAGGT AAATTCCGCC AACTCGGCAG	600
*Metarhizium_pingshaense*_ARSEF_7929	GGTGAAATTT CTAC------ ---------- -TTTCCTGCC AAGTCTAGCC TACCCGGGAG CCCAGGCAAG TCGGGCAGGT AAATTCCGCC AACTCGGCAG	600
*Metarhizium_pingshaense*_ARSEF_9975	GGTGAAATTT CTAC------ ---------- -TTTCCTGCC AAGTCTAGCC TACCCGGGAG CCCAGGCAAG TCGGGCAGGT AAATTCCGCC AACTCGGCAG	600
*Metarhizium_robertsii*_TYPE_ARSEF_2575	GGTGAAATTT CTAC------ ---------- -TTTCCTGCC AAGTCTAGTC TACCCGGGAG CCCAGGCAAG TCGGGCAGGT AAATTCCGCC AACTCGGCAG	600
*Metarhizium_robertsii*_ARSEF_727	GGTGAAATTT CTAC------ ---------- -TTTCCTGCC AAGTCTAGTC TACCCGGGAG CCCAGGCAAG TCGGGCAGGT AAATTCCGCC AACTCGGCAG	600
*Metarhizium_robertsii*_ARSEF_7501	GGTGAAATTT CTAC------ ---------- -TTTCCTGCC AAGTCTAGTC TACCCGGGAG CCCAGGCAAG TCGGGCAGGT AAATTCCGCC AACTCGGCAG	600
*Metarhizium_robertsii*_ARSEF_8070	GGTGAAATTT CTAC------ ---------- -TTTCCTGCC AAGTCTAGTC TACCCGGGAG CCCAGGCAAG TCGGGCAGGT AAATTCCGCC AACTCGGCAG	600
*Metarhizium_robertsii*_ARSEF_8685	GGTGAAATTT CTAC------ ---------- -TTTCCTGCC AAGTCTAGTC TACCCGGGAG CCCAGGCAAG TCGGGCAGGT AAATTCCGCC AACTCGGCAG	600
*Metarhizium_anisopliae*_TYPE_ARSEF_7487	GGTGAAATTT CTAC------ ---------- -TTTCCTGCC AAGTCTAGCC TACCCGGGAG CCCAGGCAAG TCGGGCAGGT AAATTCCGCC AACTCGGCAG	600
*Metarhizium_anisopliae*_ARSEF_798	GGTGAAATTT CTAC------ ---------- -TTTCCTGCC AAGTCTAGCC TACCCGGGAG CCCAGGCAAG TCGGGCAGGT AAATTCCGCC AACTCGGCAG	600
*Metarhizium_anisopliae*_ARSEF_1080	GGTGAAATTT CTAC------ ---------- -TTTCCTGCC AAGTCTAGCC TACCCGGGAG CCCAGGCAAG TCGGGCAGGT AAATTCCGCC AACTCGGCAG	600
*Metarhizium_anisopliae*_ARSEF_6347	GGTGAAATTT CTAC------ ---------- -TTTCCTGCC AAGTCTAGCC TACCCGGGAG CCCAGGCAAG TCGGGCAGGT AAATTCCGCC AACTCGGCAG	600
*Metarhizium_anisopliae*_ARSEF_7450	GGTGAAATTT CTAC------ ---------- -TTTCCTGCC AAGTCTAGCC TACCCGGGAG CCCAGGCAAG TCGGGCAGGT AAATTCCGCC AACTCGGCAG	600
*Metarhizium_humberi*_ESALQ_1638	GGTGAAATTT CTAC------ ---------- -TTTCCTGCC AAGTCTAGCC TACCCGGGAG CCCAGGCAAG TCGGGCAGGT AAATTCCGCC AACTCGGCAG	600
*Metarhizium_brunneum*_TYPE_ARSEF_2107	GGTGAAATTT CTAC------ ---------- -TTTCCTGCC AAGTCTAGCC TACCTGGGAG CCCAGGCAAG TCGGGCAGGT AAATTCCGCC AACTCGGCAG	600
*Metarhizium_brunneum*_ARSEF_1066	GGTGAAATTT CTAC------ ---------- -TTTCCTGCC AAGTCTAGCC TACCTGGGAG CCCAGGCAAG TCGGGCAGGT AAATTCCGCC AACTCGGCAG	600
*Metarhizium_brunneum*_ARSEF_1278	GGTGAAATTT CTAC------ ---------- -TTTCCTGCC AAGTCTAGCC TACCTGGGAG CCCAGGCAAG TCGGGCAGGT GAATTCCGCC AACTCGGCAG	600
*Metarhizium_brunneum*_ARSEF_3295	---------- ---------- ---------- ---------- ---------- ---------- ---------- ---------- ---------- ----------	600
*Metarhizium_brunneum*_ARSEF_3826	GGTGAAATTT CTAC------ ---------- -TTTCCTGCC AAGTCTAGCC TACCTGGGAG CCCAGGCAAG TCGGGCAGGT AAATTCCGCC AACTCGGCAG	600
*Metarhizium_brunneum*_ARSEF_4152	GGTGAAATTT CTAC------ ---------- -TTTCCTGCC AAGTCTAGCC TACCCGGGAG CCCAGGCAAG TCGGGCAGGT AAATTCCGCC AACCCGGCAG	600
*Metarhizium_brunneum*_ARSEF_4179	GGTGAAATTT CTAC------ ---------- -TTTCCTGCC AAGTCTAGCC TACCCGGGAG CCCAGGCAAG TCGGGCAGGT AAATTCCGCC AACCCGGCAG	600

mcar-IDF1	----------	----------	----------	----------	----------	----------	----------	----------	----------	----------	700
mcar-IDR1	----------	----------	----------	----------	----------	----------	----------	----------	----------	----------	700
*Metarhizium_caribense*_INISAV_LBM-30	GAAAGT-AGA	AATTTC----	----------	------ACCA	AAGTCCACAA	ATTAATCGCA	GCCTACCCGG	TA----AGCC	TGTGTAATTT	CTGTGTAATT	700
*Metarhizium_caribense*_INISAV_LBM-41	GAAAGT-AGA	AATTTC----	----------	------ACCA	AAGTCCACAA	ATTAATCGCA	GCCTACCCGG	TA----AGCC	TGTGTAATTT	CTGTGTAATT	700
*Metarhizium_caribense*_INISAV_LBM-42	GAAAGT-AGA	AATTTC----	----------	------ACCA	AAGTCCACAA	ATTAATCGCA	GCCTACCCGG	TA----AGCC	TGTGTAATTT	CTGTGTAATT	700
*Metarhizium_caribense*_ARSEF_1448	GAAAGT-AGA	AATTTC----	----------	------GCCA	A-GTCCACAA	ATTAATCGCA	GCCTACCCGG	TA----AGCC	TGTGTAATTT	CTGTGTAATT	700
*Metarhizium_caribense*_ARSEF_5197	GAAAGT-AGA	AATTTC----	----------	------ACCA	AAGTCCACAA	ATTAATCGCA	GCCTACCCGG	TA----AGCC	TGTGTAATTT	CTGTGTAATT	700
*Metarhizium_pingshaense*_TYPE_CBS_257.90	GAAACT----	AATTGCAAAT	CAATTCAAGC	AAATATGCCA	A-GTCCACAA	ATCAATCGCA	GCCTACCCGG	TA--------	----------	----------	700
*Metarhizium_pingshaense*_ARSEF_2231	GAAACT----	AATTGCAAAT	CAATTCAAGC	AAATATGCCA	A-GTCCACAA	ATCAATCGCA	GCCTACCCGG	TA--------	----------	----------	700
*Metarhizium_pingshaense*_ARSEF_2809	GAAACT----	AATTGCAAAT	CAATTCAAGC	AAATATGCCA	A-GTCCACAA	ATCAACCGCA	GCCTACCCGG	TA--------	----------	----------	700
*Metarhizium_pingshaense*_ARSEF_3180	GAAACT----	AATTGCAAAT	CAATTCAAGC	AAATATGCCA	A-GTCCACAA	ATTAATCGCA	GCCTACCCGG	TA----AGCC	TGTGTAATTT	----------	700
*Metarhizium_pingshaense*_ARSEF_3210	GAAACT----	AATTGCAAAT	CAATTCAAGC	AAATATGCCA	A-GTCCACAA	ATCAATCGCA	GCCTACCCGG	TA--------	----------	----------	700
*Metarhizium_pingshaense*_ARSEF_3604	GAAACT----	AATTGCAAAT	CAATTCAAGC	AAATGTGCCA	A-GTCCACAA	ATTAATCGCA	GCCTACCCGG	TA----AGCC	TGTGTAATTT	CTGTGTAATT	700
*Metarhizium_pingshaense*_ARSEF_4290	GAAACT----	AATTGCAAAT	CAATTCAAGC	AAATATGCCA	A-GTCCACAA	ATTAATCGCA	GCCTACCCGG	TA----AGCC	TGTGTAATTT	----------	700
*Metarhizium_pingshaense*_ARSEF_4342	GAAACT----	AATTGCAAAT	CAATTCAAGC	AAATATGCCA	A-GTCCACAA	ATTAATCGCA	GCCTACCCGG	TA----AGCC	TGTGTAATTT	----------	700
*Metarhizium_pingshaense*_ARSEF_7422	GAAACT----	AATTGCAAAT	CAATTCAAGC	AAATATGCCA	A-GTCCACAA	ATTAATCGCA	GCCTACCCGG	TA----AGCC	TGTGTAATTT	CTGTGTAATT	700
*Metarhizium_pingshaense*_ARSEF_7929	GAAACT----	AATTGCAAAT	CAATTCAAGC	AAATATGCCA	A-GTCCACAA	ATCAATCGCA	GCCTACCCGG	TA--------	----------	----------	700
*Metarhizium_pingshaense*_ARSEF_9975	GAAACT----	AATTGCAAAT	CAATTCAAGC	AAATATGCCA	A-GTCCACAA	ATCAATCGCA	GCCTACCCGG	TA--------	----------	----------	700
*Metarhizium_robertsii*_TYPE_ARSEF_2575	GGAACT----	AATTGCAAAT	CAATTCAAGC	AAATGTGCCA	A-GTCCACAA	ATCAATCGCA	GCCTACCCGG	TAGGTAAGCC	TGTGTAGTTT	CCGAGTAATT	700
*Metarhizium_robertsii*_ARSEF_727	GGAACT----	AATTGCAAAT	CAATTCAAGC	AAATGTGCCA	A-GTCCACAA	ATCAATCGCA	GCCTACCCGG	TAGGTAAGCC	TGTGTGATTT	CCGTGTAATT	700
*Metarhizium_robertsii*_ARSEF_7501	GGAACT----	AATTGCAAAT	CAATTCAAGC	AAATGTGCCA	A-GTCCACAA	ATCAATCGCA	GCCTACCTGG	TAGGTAAGCC	TGTGTAGTTT	CCGTGTAATT	700
*Metarhizium_robertsii*_ARSEF_8070	GGAACT----	AATTGCAAAT	CAATTCAAGC	AAATGTGCCA	A-GTCCACAA	ATCAATCGCA	GCCTACCCGG	TAGGTAAGCC	TGTGTGATTT	CCGTGTAATT	700
*Metarhizium_robertsii*_ARSEF_8685	GGAACT----	AATTGCAAAT	CAATTCAAGC	AAATGTGCCA	A-GTCCACAA	ATCAATCGCA	GCCTACCCGG	TAGGTAAGCC	TGTGTAGTTT	CCGAGTAATT	700
*Metarhizium_anisopliae*_TYPE_ARSEF_7487	GGAACT----	AATTGCAAAT	CAATTCGAGC	AAATATGCCA	A-GTCCACAA	ATGAATCGCA	GCCTACCCGG	TAGGTAAGCC	TGTGTAGTTT	CCGTGTAATT	700
*Metarhizium_anisopliae*_ARSEF_798	GGAACT----	AATTGCAAAT	CAATTCTAGC	AAATGTGCCA	A-GTCCACAA	ATTAATCGTA	GCCTACCCGG	TAGGTAAGCC	TGTGTAGTTT	CCGTGTAATT	700
*Metarhizium_anisopliae*_ARSEF_1080	GGAACT----	AATTGCAAAT	CAATTCGAGC	AAATGTGCCA	A-GTCCACAA	ATTAATCGTA	GCCTACCCGG	TAGGTAAGCC	TGTGTAGTTT	CCGTGTAATT	700
*Metarhizium_anisopliae*_ARSEF_6347	GGAACT----	AATTGCAAAT	CAATTCGAGC	AAATGTGCCA	A-GTCCACAA	ATTAATCGTA	GCCTACCCGG	TAGGTAAGCC	TGTGTAGTTT	CCGTGTAATT	700
*Metarhizium_anisopliae*_ARSEF_7450	GGAACT----	AATTGCAAAT	CAATTCGAGC	AAATATGCCA	A-GTCCACAA	ATGAATCGCA	GCCTACCCGG	TAGGTAAGCC	TGTGTAGTTT	CCGTGTAATT	700
*Metarhizium_humberi*_ESALQ_1638	GGAACT----	AATTGCAAAT	CAATTCAAGC	AAATGTGCCA	A-GTCCACAA	ATCAATCGCA	GCCTACCCGG	TAGGTAAGCC	TGTGTAGTTT	TCGTGTAATT	700
*Metarhizium_brunneum*_TYPE_ARSEF_2107	GGAACT----	GATTGCAAAT	TAATTTAACC	AAATCTGCCA	A-GTCCACAA	GTTAACTATA	GCCTACCCGG	GA----GGCC	TGGGTGTTTC	CCGCGTGATT	700
*Metarhizium_brunneum*_ARSEF_1066	GGAACT----	GATTGCAAAT	TAATCTAACC	AAATCTGCCA	A-GTCCACAA	GTTAACTATA	GCCTACCCGG	TA----GACC	TGGGTGTTTC	CCGCGTGATT	700
*Metarhizium_brunneum*_ARSEF_1278	GGAACT----	GATTGCAAAT	TAATTCAACC	AAATCTGCCA	A-GTCCACAA	GTTAACTATA	GCTTACCCGG	TA----GGCC	TGGGTGCTTC	CCGCGTGGTT	700
*Metarhizium_brunneum*_ARSEF_3295	----------	----------	----------	---------A	A-GTCCACAA	ATAAATTACA	GCCTACCCGG	TA----GGCC	TGTATATTTC	CCGCGCGATT	700
*Metarhizium_brunneum*_ARSEF_3826	GGAACT----	GATTGCAAAT	TAATCTAACC	AAATCTGCCA	A-GTCCACAA	GTTAACTATA	GCCTACCCGG	TA----GACC	TGGGTGTTTC	CCGCGTGATT	700
*Metarhizium_brunneum*_ARSEF_4152	GGAACT----	GATTGCAAAT	CAATTCAACC	AAATCTGCCA	A-GTCCACAG	ATAAACTATA	GCCTACCCGG	TA----GGCC	TGTAGATTTC	CCGCGCGATT	700
*Metarhizium_brunneum*_ARSEF_4179	GGAACT----	GATTGCAAAT	CAATTCAACC	AAATCTGCCA	A-GTCCACAG	ATAAACTATA	GCCTACCCGG	TA----GGCC	TGTAGATTTC	CCGCGCGATT	700

mcar-IDF1	----------	----------	----------	----------	----------	----------	----------	----------	----------	----------	800
mcar-IDR1	----------	----------	----------	----------	----------	----------	----------	----------	----------	----------	800
*Metarhizium_caribense*_INISAV_LBM-30	T-CCTGTAAA	GACCAAAAAG	TTGGGTATTT	TGGTACTTAA	TCTATGTAAG	TATAAGTTGA	TTTTTTTG-T	TTTTTTATAT	AAATAAATTT	TGCGGAAAAT	800
*Metarhizium_caribense*_INISAV_LBM-41	T-CCTGTAAA	GACCAAAAAG	TTGGGTATTT	TGGTACTTAA	TCTATGTAAG	TATAAGTTGA	TTTTTTTG-T	TTTTTTATAT	AAATAAATTT	TGCGGAAAAT	800
*Metarhizium_caribense*_INISAV_LBM-42	T-CCTGTAAA	GACCAAAAAG	TTGGGTATTT	TGGTACTTAA	TCTATGTAAG	TATAAGTTGA	TTTTTTTG-T	TTTTTTATAT	AAATAAATTT	TGCGGAAAAT	800
*Metarhizium_caribense*_ARSEF_1448	T-CCTGTAAA	GACCAAAAAG	TTGGGTATTT	TGGTACTTAA	TTTATGTAAG	TATAAGTTGA	TTTTTTTG-T	TTTTTTATAT	AAATAAATTT	TGCGGAAAAT	800
*Metarhizium_caribense*_ARSEF_5197	T-CCTGTAAA	GACCAAAAAG	TTGGGTATTT	TGGTACTTAA	TCTATGTAAG	TATAAGTTGA	TTTTTTTG-T	TTTTTTATAT	AAATAAATTT	TGCGGAAAAT	800
*Metarhizium_pingshaense*_TYPE_CBS_257.90	----------	----------	----------	----------	--------AG	TATAAGTTGA	TTTTTTT--T	T-ATATATAT	AAATAAATTC	TGCGGAAAAT	800
*Metarhizium_pingshaense*_ARSEF_2231	----------	----------	----------	----------	--------AG	TATAAGTTGA	TTTTTTT--T	TTATATATAT	AAATAAATTT	TGCGGAAAAT	800
*Metarhizium_pingshaense*_ARSEF_2809	----------	----------	----------	----------	--------AG	TATAAGTTGA	TCTTTTT--T	TTATATATGT	AAATAAATTT	TGCGGAAAAT	800
*Metarhizium_pingshaense*_ARSEF_3180	----------	----------	----------	----------	----------	----------	----------	----------	--------TT	TGCGGAAAAT	800
*Metarhizium_pingshaense*_ARSEF_3210	----------	----------	----------	----------	--------AG	TATAAGTTGA	TTTTTTT--T	TTATATATAT	AAATAAATTT	TGCGGAAAAT	800
*Metarhizium_pingshaense*_ARSEF_3604	T-CCTGTAAA	GACCAAAAAG	TTGGGTATTT	TAGTACCTAA	TTTATATAAG	TATAAGTTGA	TTTTTTTG-T	TTTTTTATAT	AAATAAATTT	TGCGGAAAAT	800
*Metarhizium_pingshaense*_ARSEF_4290	----------	----------	----------	----------	----------	----------	----------	----------	--------TT	TGCGGAAAAT	800
*Metarhizium_pingshaense*_ARSEF_4342	----------	----------	----------	----------	----------	----------	----------	----------	--------TT	TGCGGAAAAT	800
*Metarhizium_pingshaense*_ARSEF_7422	T-CCCGTAAA	GACCAAAAAG	TTGGGTATTT	TAGTACTTAA	TTTATATAAG	TATAAGTTGA	TTTTTTTG-T	TTTTTTATAT	AAATAAATTT	TGCGGAAAAT	800
*Metarhizium_pingshaense*_ARSEF_7929	----------	----------	----------	----------	--------AG	TATAAGTTGA	TTTTTTT--T	TTATATATAT	AAATAAATTT	TGCGGAAAAT	800
*Metarhizium_pingshaense*_ARSEF_9975	----------	----------	----------	----------	--------AG	TATAAGTTGA	TTTTTTTT-T	TTATATATAT	AAATAAATTT	TGCGGAAAAT	800
*Metarhizium_robertsii*_TYPE_ARSEF_2575	TTCCCGTAAA	GACCAAAAAG	TCAGGTATTT	TGGTACTTAA	TTTATATAAG	TACAAGTTGA	TTTTTTTG-T	TTTTTTATAT	AAATAAATTT	TGCGGAAAAT	800
*Metarhizium_robertsii*_ARSEF_727	T-CCCGTAAA	GACCAAAAAG	TTAGGTATTT	TGGTACTTAA	TTTATATAAG	TACAAGTTGA	TTTTTTTG-T	TTTTTTATAT	AAATAAATTT	TGCGGAAAAT	800
*Metarhizium_robertsii*_ARSEF_7501	TTCCCGTAAA	GACCAAAAAG	TCAGGTATTT	TGGTACTTAA	TTTATATAAG	TACAAGTTGA	TTTTTTTG-T	TTTTTTATAT	AAATAAATTT	TGCGGAAAAT	800
*Metarhizium_robertsii*_ARSEF_8070	T-CCCGTAAA	GACCAAAAAG	TTAGGTATTT	TGGTACTTAA	TTTATATAAG	TACAAGTTGA	TTTTTTTG-T	TTTTTTATAT	AAATAAATTT	TGCGGAAAAT	800
*Metarhizium_robertsii*_ARSEF_8685	TTCCCGTAAA	GACCAAAAAG	TCAGGTATTT	TGGTACTTAA	TTTATATAAG	TACAAGTTGA	TTTTTTTG-T	TTTTTTATAT	AAATAAATTT	TGCGGAAAAT	800
*Metarhizium_anisopliae*_TYPE_ARSEF_7487	TTCCCGTAAA	GACCAAAAAG	TCAGGTGTTT	TAGTATTTAA	TTTATATAGG	CGTGAGTTGA	TTTTTTTG-T	TTTTCTATAT	TAATAAATTT	TGCGGAAAAT	800
*Metarhizium_anisopliae*_ARSEF_798	TTCCCGCAAA	GACCAAAAAG	TCAGGTGTTT	TAGTATTTAA	TTTATATAGG	CGTGAGTTGA	TTTTTTTG-T	TTTTTTATAT	TAATAAATTT	TGCGGAAAAT	800
*Metarhizium_anisopliae*_ARSEF_1080	TTCCCGCAAA	GACCAAAAAG	TCAGGTGTTT	TAGTATTTAA	TTTATATAGG	CGTGAGTTGA	TTTTTTTG-T	TTTTTTATAT	TAATAAATTT	TGCGGAAAAT	800
*Metarhizium_anisopliae*_ARSEF_6347	TTCCCGCAAA	GACCAAAAAG	TCAGGTGTTT	TAGTATTTAA	TTTATATAGG	CGTGAGTTGA	TTTTTTTG-T	TTTTTTATAT	TAATAAATTT	TGCGGAAAAT	800
*Metarhizium_anisopliae*_ARSEF_7450	TTCCCGTAAA	GACCAAAAAG	TCAGGTGTTT	TAGTATTTAA	TTTATATAGG	CGTGAGTTGA	TTTTTTTG-T	TTTTCTATAT	TAATAAATTT	TGCGGAAAAT	800
*Metarhizium_humberi*_ESALQ_1638	TTCCCGCAAA	GACCAAAAAG	TCGGGTGTTT	TAGTATTTGA	TTTGTATGGG	CGTGAGTTGA	TTTTTTTA-T	TTTCCCATAT	AAATAAATTT	TGCGGAAAAT	800
*Metarhizium_brunneum*_TYPE_ARSEF_2107	T-CCTGTAAA	AACAAAAAAG	TTAGGTGTTT	TAGTACTTAA	TTTATATAGG	TATAAGTTAA	TTTTTTTG-T	TTTCTTATAT	AAATAAAATT	TGCGGAAAAT	800
*Metarhizium_brunneum*_ARSEF_1066	T-CCTGTGAA	AACAAAAAAG	TTAGGTGTTT	TAGTACTTAA	TTTATGTAGG	TATGGGTTGA	TTTTTTTG-T	TTTCTTATAT	AAATAAAATT	TGCGGAAAAT	800
*Metarhizium_brunneum*_ARSEF_1278	T-CCTGTAAA	AACAAAAAAG	TTAGGTGTTT	TAGTACTTAA	TTTATATAGG	TATAAGTTGA	TTTTTTTG-T	TTTCTTATAT	AAATAAAATT	TGCGGAAAAT	800
*Metarhizium_brunneum*_ARSEF_3295	T-CCTGTGAA	GACCAAAAAG	TTAGGTGTTT	TAGTACTTAA	TTTATATAGG	TATAAGTTGA	TTTTTTTGTT	TTTTTTATAT	ATATAAATTT	TGCGGGAAAT	800
*Metarhizium_brunneum*_ARSEF_3826	T-CCTGTGAA	AACAAAAAAG	TTAGGTGTTT	TAGTACTTAA	TTTATATAGG	TATGGGTTGA	TTTTTTTG-T	TTTCTTATAT	AAATAAAATT	TGCGGAAAAT	800
*Metarhizium_brunneum*_ARSEF_4152	T-CCTGCGAA	GACCAAAAAG	TTAGGTGTTT	TGGTACTTAA	TTTATATAGA	GATGAGTTGA	TTTTTTTG-T	TTTTATATAT	AAATAAATTT	TGCGGAAAAT	800
*Metarhizium_brunneum*_ARSEF_4179	T-CCTGCGAA	GACCAAAAAG	TTAGGTGTTT	TGGTACTTAA	TTTATATAGA	GATGAGTTGA	TTTTTTTG-T	TTTTATATAT	AAATAAATTT	TGCGGAAAAT	800

mcar-IDF1	----------	----------	----------	----------	----------	----------	----------	----------	----------	----------	900
mcar-IDR1	----------	----------	----------	----------	-**CAAGGTAGT**	**TGGTGGGTAT**	**ATAAGA**----	----------	----------	----------	900
*Metarhizium_caribense*_INISAV_LBM-30	AAAAAATAAG	CTATAAAAGC	CTGGTAGCGG	AGGCGGGCTC	TCAAGGTAGT	TGGTGGGTAT	ATAAGAGGGA	GGGCTGGACA	CGCGTTGCGC	CGCGTATTTC	900
*Metarhizium_caribense*_INISAV_LBM-41	AAAAAATAAG	CTATAAAAGC	CTGGTAGCGG	AGGCGGGCTC	TCAAGGTAGT	TGGTGGGTAT	ATAAGAGGGA	GGGCTGGACA	CGCGTTGCGC	CGCGTATTTC	900
*Metarhizium_caribense*_INISAV_LBM-42	AAAAAATAAG	CTATAAAAGC	CTGGTAGCGG	AGGCGGGCTC	TCAAGGTAGT	TGGTGGGTAT	ATAAGAGGGA	GGGCTGGACA	CGCGTTGCGC	CGCGTATTTC	900
*Metarhizium_caribense*_ARSEF_1448	AAAAAATAAG	CTATAAAAGC	CTGGTAGCGG	AGGCGGGCTC	TCAAGGTAGT	TGGTGGGTAT	ATAATAGGGA	GGGCTGGACA	CGCGTTGCGT	CGCGTATTTC	900
*Metarhizium_caribense*_ARSEF_5197	AAAAAATAAG	CTATAAAAGC	CTGGTAGCGG	AGGCGGGCTC	TCAAGGTAGT	TGGTGGGTAT	ATAAGAGGGA	GGGCTGGACA	CGCGTTGCGC	CGCGTATTTC	900
*Metarhizium_pingshaense*_TYPE_CBS_257.90	AAAAAATAAG	CTATAAAGGC	CTGGTAGCGG	AGGCGAGCTC	TTAAGGTAGT	GGGTGGGTAT	ATAAGAGGGA	GGGCTGGACA	CGCGTTGCGC	CGCGTATTTC	900
*Metarhizium_pingshaense*_ARSEF_2231	AAAAAATAAG	CTATAAAGGC	CTGGTAGCGG	AGGCGAGCTC	TTAAGGTATT	GGGTGGGTGT	ATAAGAGGGA	GGGCTGGACA	CGCGTTGCGC	CGCGTATTTC	900
*Metarhizium_pingshaense*_ARSEF_2809	AAAAAATAAG	CTATAAAAGC	CTGGTAGCGG	AGGCGAGCTC	TTAAGATAGT	TGGTGGGTAT	ATAAGAGGGA	GGGCTGGACA	CGCGTTACGC	CGCGTATTTC	900
*Metarhizium_pingshaense*_ARSEF_3180	AAAAAATAAG	CTATAAAAGC	CTGGTAGCGG	AGGCGGGCTC	TTAAGGTAGT	TGGTGGGTAT	ATAAGAGGGA	GGGCTGGACA	CGCGTTGCGT	CGCGTATTTC	900
*Metarhizium_pingshaense*_ARSEF_3210	AAAAAATAAG	CTATAAAGGC	CTGGTAGCGG	AGGCGAGCTC	TTAAGGTAGT	GGGTGGGTAT	ATAAGAGGGA	GGGCTGGACA	CGCGTTGCGC	CGCGTATTTC	900
*Metarhizium_pingshaense*_ARSEF_3604	AAAAAATAAG	CTATAAAAGC	CTGGTAGCGG	AGGCGGGCTC	TTAAGGTAGT	TGGCGGGCAT	ATAAGAGGGA	GGGCTGGACA	CGCGTTGCGC	CGCGTATTTC	900
*Metarhizium_pingshaense*_ARSEF_4290	AAAAAATAAG	CTATAAAAGC	CTGGTAGCGG	AGGCGGGCTC	TTAAGGTAGT	TGGTGGGTAT	ATAAGAGGGA	GGGCTGGACA	CGCGTTGCGT	CGCGTATTTC	900
*Metarhizium_pingshaense*_ARSEF_4342	AAAAAATAAG	CTATAAAAGC	CTGGTAGCGG	AGGCGGGCTC	CTAAGGTAGT	TGGTGGGTAT	ATAAGAGGGA	GGGCTGGACA	CGCGTTGCGT	CGCGTATTTC	900
*Metarhizium_pingshaense*_ARSEF_7422	AAAAAATAAG	CTATAAAAGC	CTGGTAGCGC	AGGCGGGCTC	TTAAGGTAGT	TGGTGGGTAT	ATAAGAGGGA	GGGCTGGAGA	CGCGTTGCGC	CCCGTATTTC	900
*Metarhizium_pingshaense*_ARSEF_7929	AAAAAATAAG	CTATAAAGGC	CTGGTAGCGG	AGGCGAGCTC	TTAAGGTAGT	GGGTGGGTAT	ATAAGAGGGA	GGGCTGGACA	CGCGTTGCGC	CGCGTATTTC	900
*Metarhizium_pingshaense*_ARSEF_9975	AAAAAAAAAG	CTATAAAGGC	CTGGTAGCGG	AGGCGAGCTC	TTAAGGTAGT	GGGTGGGTAT	ATAAGAGGGA	GGGCTGGACA	CGCGTTGCGC	CGCGTATTTC	900
*Metarhizium_robertsii*_TYPE_ARSEF_2575	AAAAAATAAG	CTATAAAAGC	CTGGTAGCGG	AGGCGGGCCC	TTAGGGTAGT	TGGTGGGTAT	ATGAGAGGGA	GGGCTGGACA	CGCGTCGCGT	CGCGTATTTC	900
*Metarhizium_robertsii*_ARSEF_727	AAAAAATAAG	CTATAAAAGC	CTGGTAGCGG	AGGCGGGCCC	TTAGGGTAGT	TGGTGGGTAT	ATGAGAGGGA	GGGCTGGACA	CGCGTCGCGT	CGCGTATTTC	900
*Metarhizium_robertsii*_ARSEF_7501	AAAAAATAAG	CTATAAAAGC	CTGGTAGCGG	AGGCGGGCCC	TTAGGGTAGT	TGGTGGGTAT	ATGAGAGGGA	GGGCTGGACA	CGCGTCGCGT	CGCGTATTTC	900
*Metarhizium_robertsii*_ARSEF_8070	AAAAAATAAG	CTATAAAAGC	CTGGTAGCGG	AGGCGGGCCC	TTAGGGTAGT	TGGTGGGTAT	ATGAGAGGGA	GGGCTGGACA	CGCGTCGCGT	CGCGTATTTC	900
*Metarhizium_robertsii*_ARSEF_8685	AAAAAATAAG	CTATAAAAGC	CTGGTAGCGG	AGGCGGGCCC	TTAGGGTAGT	TGGTGGGTAT	ATGAGAGGGA	GGGCTGGACA	CGCGTCGCGT	CGCGTATTTC	900
*Metarhizium_anisopliae*_TYPE_ARSEF_7487	AAAAAATAAG	CTATAAAAAC	CTGGTAACAG	AGGCGGGCCC	TTAAGGTAAT	TGGTGGGTAT	ATAAGAGGGA	GGGCTGGACA	CGCGTTGCGT	TGTGTATTTC	900
*Metarhizium_anisopliae*_ARSEF_798	AAAAAATAAG	CTATAAAAAC	CTGGTAACAA	AGGCGGGCTT	TTAAGGTAAT	TGGTGGGTAT	ATAAGAGGGA	GGGCTGGACA	CGCGTTGCGT	TGTGTATTTC	900
*Metarhizium_anisopliae*_ARSEF_1080	AAAAAATAAG	CTATAAAAAC	CTGGTAACAA	AGGCGGGCTT	TTAAGGTAAT	TGGTGGGTAT	ATAAGAGGGA	GGGCTGGACA	CGCGTTGCGT	TGTGTATTTC	900
*Metarhizium_anisopliae*_ARSEF_6347	AAAAAATAAG	CTATAAAAAC	CTGGTAACAA	AGGCGGGCTT	TTAAGGTAAT	TGGTGGGTAT	ATAAGAGGGA	GGGCTGGACA	CGCGTTGCGT	TGTGTATTTC	900
*Metarhizium_anisopliae*_ARSEF_7450	AAAAAATAAG	CTATAAAAAC	CTGGTAACAG	AGGCGGGCCC	TTAAGGTAAT	TGGTGGGTAT	ATAAGAGGGA	GGGCTGGACA	CGCGTTGCGT	TGTGTATTTC	900
*Metarhizium_humberi*_ESALQ_1638	AAAAAATAAG	CTATAAAAGC	CTGGTAACAG	AGGCGGGCCC	TTAAGGTAGT	TGGTGGGTAT	ATAAGAGGAA	GGGCTGGACA	CGCGTTGCGT	CGCGTATTTC	900
*Metarhizium_brunneum*_TYPE_ARSEF_2107	AAAAAATAAA	CCACGAGAGC	CCGGTAGCGG	AGGCAAGCCC	TTAAGGTAGT	CGGTAAGTAT	ATAAGAGAGA	GGGCTGGACA	CGCGTCGCGT	CGCGTATTTC	900
*Metarhizium_brunneum*_ARSEF_1066	AAAAAATAAA	CCACGAGAGC	CCGGTAGCGG	AGGCGAGCCC	TTAAGGTAGT	CGGTAAGTAT	ATAAGAGAGA	GGGCTGGACA	CGCGTCGCGT	CGCGTATTTC	900
*Metarhizium_brunneum*_ARSEF_1278	AAAAAATAAA	CCACGAGAGC	CCGGTAGCGG	AGGCGAGCCC	TTAAGGTAGT	CGGTAAGTAT	ATAAGAGAGA	GGGCTGGACA	CGCGTCGCGT	CGCGTATTTC	900
*Metarhizium_brunneum*_ARSEF_3295	AAAAAATAAA	CCACGAGAGC	CCGGTAGCGG	AGGCGAGCCC	TTAAGGTAGT	CGGTAAGTAT	ATAAGAGAGA	GGGCTGGACA	CGCGTCGCGT	CGCGTATTTC	900
*Metarhizium_brunneum*_ARSEF_3826	AAAAAATAAA	CCACGAGAGC	CCGGTAGCGG	AGGCGAGCCC	TTAAGGTAGT	CGGTAAGTAT	ATAAGAGAGA	GGGCTGGACA	CGCGTCGCGT	CGCGTATTTC	900
*Metarhizium_brunneum*_ARSEF_4152	AAAAAATAAA	CCACGAGAGC	CTAGTAGCGG	AGGCGGGCCC	TCGAGGTGGT	CGGTAGGTAT	ATAAGAGAGA	GGGCTGGACA	CGCGTCGCGT	CGCGTATTTC	900
*Metarhizium_brunneum*_ARSEF_4179	AAAAAATAAA	CCACGAGAGC	CTAGTAGCGG	AGGCGGGCCC	TCGAGGTGGT	CGGTAGGTAT	ATAAGAGAGA	GGGCTGGACA	CGCGTCGCGT	CGCGTATTTC	900

```
mcar-IDF1                                   ---------- ---------- ---------- ---------- ---------- ---------- ---------- ---------- ---------- ----------  1000
mcar-IDR1                                   ---------- ---------- ---------- ---------- ---------- ---------- ---------- ---------- ---------- ----------  1000
Metarhizium_caribense_INISAV_LBM-30         CACCGCACCC AATACTTTTA ACTTTAGGGT AGGCTGCTTG TTTAGAGGCG TGCTGAATTA AATGGTCTCT CAAGTGAGGG GATTTCTCTG CTGGCAGTTG  1000
Metarhizium_caribense_INISAV_LBM-41         CACCGCACCC AATACTTTTA ACTTTAGGGT AGGCTGCTTG TTTAGAGGCG TGCTGAATTA AATGGTCTCT CAAGTGAGGG GATTTCTCTG CTGGCAGTTG  1000
Metarhizium_caribense_INISAV_LBM-42         CACCGCACCC AATACTTTTA ACTTTAGGGT AGGCTGCTTG TTTAGAGGCG TGCTGAATTA AATGGTCTCT CAAGTGAGGG GATTTCTCTG CTGGCAGTTG  1000
Metarhizium_caribense_ARSEF_1448            CACCGCACCC AATACTTTTA GCTTTAGGGT AGGCTGCTTG TTTAGAGGCG TGCTGAATTA AATGGTCTCT CAAGTGAGGG GATTTCTCTG CTGGCAGTTG  1000
Metarhizium_caribense_ARSEF_5197            CACCGCACCC AATACTTTTA ACTTTAGGGT AGGCTGCTTG TTTAGAGGCG TGCTGAATTA AATGGTCTCT CAAGTGAGGG GATTTCTCTG CTGGCAGTTG  1000

Metarhizium_pingshaense_TYPE_CBS_257.90     CACCGCACCC AATACTTTTA GCTTTAGGGT AGGCTGCTTG TTTAGAGGCG TGCTGAATTA AATGGTCTCT CAAGTGAGGG GATTTCTCTG CTGGCAGTTG  1000
Metarhizium_pingshaense_ARSEF_2231          CACCGCACCC AATACTTTTA GCTTTAGGGT AGGCTGCTTG TTTAGAGGCG TGCTGAATTA AATGGTCTCT CAAGTGAGGG GATTTCTCTG CTGGCAGTTG  1000
Metarhizium_pingshaense_ARSEF_2809          CACCGCACCC AATACTTTTA GCTTTAGGGT AGGCTGCTTG TTTAGAGGCG TGCTGAATTA AATGGTCTCT CAAGTGAGGG GATTTCTCTG CTGGCAGTTG  1000
Metarhizium_pingshaense_ARSEF_3180          CACCGCACCC AATACTTTTA GCTTTAGGGT AGGCTGCTTG TTTAGAGGCG TGCTGAATTA AATGGTCTCT CAAGTGAGGG GATTTCTCTG CTGGCAGTTG  1000
Metarhizium_pingshaense_ARSEF_3210          CACCGCACCC AATACTTTTA GCTTTAGGGT AGGCTGCTTG TTTAGAGGCG TGCTGAATTA AATGGTCTCT CAAGTGAGGG GATTTCTCTG CTGGCAGTTG  1000
Metarhizium_pingshaense_ARSEF_3604          CACCGCACCC AATACTTTTA GCTTTAGGGT AGGCTGCTTG TTTAGAGGCG TGCTGAATTA AATGGTCTCT CAAGTGAGGG GATTTCTCTG CTGGCAGTTG  1000
Metarhizium_pingshaense_ARSEF_4290          CACCGCACCC AATACTTTTA GCTTTAGGGT AGGCTGCTTG TTTAGAGGCG TGCTGAATTA AATGGTCTCT CAAGTGAGGG GATTTCTCTG GCTGGAGTTG  1000
Metarhizium_pingshaense_ARSEF_4342          CACCGCACCC AATACTTTTA GCTTTAGGGT AGGCTGCTTG TTTAGAGGCG TGCTGAATTA AATGGTCTCT CAAGTGAGGG GATTTCTCTG CTGGCAGTTG  1000
Metarhizium_pingshaense_ARSEF_7422          CACCGTACCC AATACTTTTA GCTTTAGGGT AGGCTGCTTG TTTAAAGGCG TGCTGAATTA AATGGTCTCT TAAGTGAGGG GATTTCTCTG CTGGCAGTTG  1000
Metarhizium_pingshaense_ARSEF_7929          CACCGCACCC AATACTTTTA GCTTTAGGGT AGGCTGCTTG TTTAGAGGCG TGCTGAATTA AATGGTCTCT CAAGTGAGGG GATTTCTCTG CTGGCAGTTG  1000
Metarhizium_pingshaense_ARSEF_9975          CACCGCACCC AATACTTTTA GCTTTAGGGT AGGCTGCTTG TTTAGAGGCG TGCTGAATTA AATGGTCTCT CAAGTGAGGG GATTTCTCTG CTGGCAGTTG  1000

Metarhizium_robertsii_TYPE_ARSEF_2575       CGCCGCAGCC AATACTTTTA GCTTTAGGGT AGGCTGCTTC TTTAGAGGCG TGCTGAATTA AATGGTCTCT TGAGTGAGGG GATTTCTCTG CTGGCAGTTG  1000
Metarhizium_robertsii_ARSEF_727             CGCCGCAGCC AATACTTTTA GCTTTAGGGT AGGCTGCTTC TTTAGAGGCG TGCTGAATTA AATGGTCTCT TGAGTGAGGG GATTTCTCTG CTGGCAGTTG  1000
Metarhizium_robertsii_ARSEF_7501            CGCCGCAGCC AATACTTTTA GCTTTAGGGT AGGCTGCTTC TTTAGAGGCG TGCTGAATTA AATGGTCTCT TGAGTGAGGG GATTTCTCTG CTGGCAGTTG  1000
Metarhizium_robertsii_ARSEF_8070            CGCCGCAGCC AATACTTTTA GCTTTAGGGT AGGCTGCTTC TTTAGAGGCG TGCTGAATTA AATGGTCTCT TGAGTGAGGG GATTTCTCTG CTGGCAGTTG  1000
Metarhizium_robertsii_ARSEF_8685            CGCCGCAGCC AATACTTTTA GCTTTAGGGT AGGCTGCTTC TTTAGAGGCG TGCTGAATTA AATGGTCTCT TGAGTGAGGG GATTTCTCTG CTGGCAGTTG  1000

Metarhizium_anisopliae_TYPE_ARSEF_7487      CACCACACCC AATACTTTTA GCTTTAGGGT AGGCTGCTTG TTCAGAGGCG TGCTGAATTA AATGGTCTCT TGAGTGAGGG GATTTCTCTG CTGGCAGTTG  1000
Metarhizium_anisopliae_ARSEF_798            TACCATACCC AATACTTTTA GCTTTAGGGT AGGCTGCTTG TTTAGAGGCG TGCTGAATTA AATGGTCTCT TGAGTGAGGG GATTTCTCTG CTGGCAGTTG  1000
Metarhizium_anisopliae_ARSEF_1080           TACCATACCC AATACTTTTA GCTTTAGGGT AGGCTGCTTG TTTAGAGGCG TGCTGAATTA AATGGTCTCT TGAGTGAGGG GATTTCTCTG CTGGCAGTTG  1000
Metarhizium_anisopliae_ARSEF_6347           TACCATACCC AATACTTTTA GCTTTAGGGT AGGCTGCTTG TTTAGAGGCG TGCTGAATTA AATGGTCTCT TGAGTGAGGG GATTTCTCTG CTGGCAGTTG  1000
Metarhizium_anisopliae_ARSEF_7450           CACCACACCC AATACTTTTA GCTTTAGGGT AGGCTGCTTG TTCAGAGGCG TGCTGAATTA AATGGTCTCT TGAGTGAGGG GATTTCTCTG CTGGCAGTTG  1000

Metarhizium_humberi_ESALQ_1638              CACCGCACCC AATACTTTTA GCTTTAGGGT AGGCTGCTTG TTTAGAGGCG CGCTGGATTA AATGGTCTCT TGAGTGAGGG GATTTCTCTG CTGGCAGCTG  1000

Metarhizium_brunneum_TYPE_ARSEF_2107        CACCGCACCT AATACTTTTG GCTTTAGGGT AGGCTGCTTG TTTAGAGGCG CGCTGAATTA GATGGTCTCT TAAGTGAGGG GATTTCTCTG CTGGCAGTTG  1000
Metarhizium_brunneum_ARSEF_1066             CACCGCACCT AATACTTTTG GCTTTAGGGT AGGCTGCTTG TTTAGAGGCG CGCTGAATTA GATGGTCTCT TAAGTGAGGG GATTTCTCTG GTGGCAGTTG  1000
Metarhizium_brunneum_ARSEF_1278             CACCGCACCT AATACTTTTG GCTTTAGGGT AGGCTGCTTG TTTAGAGGCG CGCTGAATTA GATGGTCTCT TAAGTGAGGG GATTTCTCTG CTGGCAGTTG  1000
Metarhizium_brunneum_ARSEF_3295             CACCGCACCT AATACTTTTG GCTTTAGGGT AGGCTGCTTG TTTAGAGGCG CGCTGAATTA GATGGTCTCT TAAGTGAGGG GATTTCTCTG CTGGCAGTTG  1000
Metarhizium_brunneum_ARSEF_3826             CACCGCACCT AATACTTTTG GCTTTAGGGT AGGCTGCTTG TTTAGAGGCG CGCTGAATTA GATGGTCTCT TAAGTGAGGG GATTTCTCTG GTGGCAGTTG  1000
Metarhizium_brunneum_ARSEF_4152             CACCGCAGCC AATACCTTTA GCTTTAGGGT AGGCTGCTTG TTTAGAGGCG CGCTGGATTA GATGGTCTCT CAAGTGAGGG GATTTCTCTG CTGGCAGTTG  1000
Metarhizium_brunneum_ARSEF_4179             CACCGCAGCC AATACCTTTA GCTTTAGGGT AGGCTGCTTG TTTAGAGGCG CGCTGGATTA GATGGTCTCT CAAGTGAGGG GATTTCTCTG CTGGCAGTTG  1000
```

mcar-IDF1	---------- ---------- ---------- ---------- ---------- ---------- ---------- ---------- ---------- ----------	1100
mcar-IDR1	---------- ---------- ---------- ---------- ---------- ---------- ---------- ---------- ---------- ----------	1100
*Metarhizium_caribense*_INISAV_LBM-30	CTTGTGATCC GGGAGTCCGT GGCGGTAAAG TCAACTGTAA AGCTTGTGTG TGTG------ ---------- ----CCGGGG CCCTGTAAGT CCCGACCAGG	1100
*Metarhizium_caribense*_INISAV_LBM-41	CTTGTGATCC GGGAGTCCGT GGCGGTAAAG TCAACTGTAA AGCTTGTGTG TGTG------ ---------- ----CCGGGG CCCTGCAAGT CCCGACCAGG	1100
*Metarhizium_caribense*_INISAV_LBM-42	CTTGTGATCC GGGAGTCCGT GGCGGTAAAG TCAACTGTAA AGCTTGTGTG TGTG------ ---------- ----CCGGGG CCCTGTAAGT CCCGACCAGG	1100
*Metarhizium_caribense*_ARSEF_1448	CTTGTGATCC GGGAGTCCGT GGCGGTAAAG TCAACTGTAA AGCTTGTGTG TGTG------ ---------- ----CCGGGG CCCTGTAAGT CCCGACCAGG	1100
*Metarhizium_caribense*_ARSEF_5197	CTTGTGATCC GGGAGTCCGT GGCGGTAAAG TCAACTGTAA AGCTTGTGTG TGTG------ ---------- ----CCGGGG CCCTGTAAGT CCCGACCAGG	1100
*Metarhizium_pingshaense*_TYPE_CBS_257.90	CTTGTGATCC GGGAGTCCGT GGCGGTAAAG TCAACTGTAA GGCTTGTGTG TGTG------ ---------- ----CCGGGG CCCTGTAGGT CCCGACCAGG	1100
*Metarhizium_pingshaense*_ARSEF_2231	CTTGTGATCC GGGAGTCCGT GGCGGTAAAG TCAACTGTAA GGCTTGTGTG TGTG------ ---------- ----CCGGGG CCCTGTAGGT CCCGACCAGG	1100
*Metarhizium_pingshaense*_ARSEF_2809	CTTGTGATCC GGGAGTCCGT GGCGGTAAAG TCAACTGTAA GGCTTGTGTG TGTG------ ---------- ----CCGGGG CCCTGTAGGT CCCGACCAGG	1100
*Metarhizium_pingshaense*_ARSEF_3180	CTTGTGATCC GGGAGTCCGT GGCGGTAAAG TCAACTGTAA GGCTTGTGTG TGTG------ ---------- ----CCGGGG CCCTGTAGGT CCCGACCAGG	1100
*Metarhizium_pingshaense*_ARSEF_3210	CTTGTGATCC GGGAGTCCGT GGCGGTAAAG TCAACTGTAA GGCTTGTGTG TGTG------ ---------- ----CCGGGG CCCTGTAGGT CCCGACCAGG	1100
*Metarhizium_pingshaense*_ARSEF_3604	CTTGTGATCC GGGAGTCCGT GGCGGTAAAG TCAACTGTGA GGCTTGTGTG TGTG------ ---------- ----CCGGGG CCCTGTAGGT CCCGACCAGG	1100
*Metarhizium_pingshaense*_ARSEF_4290	CTTGTGATCC GGGAGTCCGT GGCGGTAAAG TCAACTGTAA GGCTTGTGTG TGTG------ ---------- ----CCGGGG CCCTGTAGGT CCCGACCAGG	1100
*Metarhizium_pingshaense*_ARSEF_4342	CTTGTGATCC GGGAGTCCGT GGCGGTAAAG TCAACTGTAA GGCTTGTGTG TGTG------ ---------- ----CCGGGG CCCTGTAGGT CCCGACCAGG	1100
*Metarhizium_pingshaense*_ARSEF_7422	CTTGTGATCC GGGAGTCCGT GGCGGTAAAG TCAACTGTAA GGCTTGCGTG TGTG------ ---------- ----CCGGGG CCCTGTAGGT CCCGACCAGG	1100
*Metarhizium_pingshaense*_ARSEF_7929	CTTGTGATCC GGGAGTCCGT GGCGGTAAAG TCAACTGTAA GGCTTGTGTG TGTG------ ---------- ----CCGGGG CCCTGTAGGT CCCGACCAGG	1100
*Metarhizium_pingshaense*_ARSEF_9975	CTTGTGATCC GGGAGTCCGT GGCGGTAAAG TCAACTGTAA GGCTTGTGTG TGTG------ ---------- ----CCGGGG CCCTGTAGGT CCCGACCAGG	1100
*Metarhizium_robertsii*_TYPE_ARSEF_2575	CCTGTGATCC GGGAGTCCGT GGCGGTAAAG TCAACTGTAA GGCTTGTGTG TGTG------ ---------- ----CCGGGG CCCTGTAAGT CCCGACCAGG	1100
*Metarhizium_robertsii*_ARSEF_727	CCTGTGATCC GGGAGTCCGT GGCGGTAAAG TCAACTGTAA GGCTTGTGTG TGTG------ ---------- ----CCGGGG CCCTGTAAGT CCCGACCAGG	1100
*Metarhizium_robertsii*_ARSEF_7501	CCTGTGATCC GGGAGTCCGT GGCGGTAAAG TCAACTGTAA GGCTTGTGTG TGTG------ ---------- ----CCGGGG CCCTGTAAGT CCCGACCAGG	1100
*Metarhizium_robertsii*_ARSEF_8070	CCTGTGATCC GGGAGTCCGT GGCGGTAAAG TCAACTGTAA GGCTTGTGTG TGTG------ ---------- ----CCGGGG CCCTGTAAGT CCCGACCAGG	1100
*Metarhizium_robertsii*_ARSEF_8685	CCTGTGATCC GGGAGTCCGT GGCGGTAAAG TCAACTGTAA GGCTTGTGTG TGTG------ ---------- ----CCGGGG CCCTGTAAGT CCCGACCAGG	1100
*Metarhizium_anisopliae*_TYPE_ARSEF_7487	CCTGTGATCC GGGAGTCCGT GGCGGTAAAG TCAACTGTAA GGCTTGTGTG TGTG------ ---------- ----CCGGGG CCCTGTAAGT CCCGACCAGG	1100
*Metarhizium_anisopliae*_ARSEF_798	CCTGTGATCC GGGAGTCCGT GGCGGTAAAG TCAACTGTAA GGCTTGTGTG TGTG------ ---------- ----CCGGGG CCCTGTAAGT CCCGACCAGG	1100
*Metarhizium_anisopliae*_ARSEF_1080	CCTGTGATCC GGGAGTCCGT GGCGGTAAAG TCAACTGTAA GGCTTGTGTG TGTG------ ---------- ----CCGGGG CCCTGTAAGT CCCGACCAGG	1100
*Metarhizium_anisopliae*_ARSEF_6347	CCTGTGATCC GGGAGTCCGT GGCGGTAAAG TCAACTGTAA GGCTTGTGTG TGTG------ ---------- ----CCGGGG CCCTGTAAGT CCCGACCAGG	1100
*Metarhizium_anisopliae*_ARSEF_7450	CCTGTGATCC GGGAGTCCGT GGCGGTAAAG TCAACTGTAA GGCTTGTGTG TGTG------ ---------- ----CCGGGG CCCTGTAAGT CCCGACCAGG	1100
*Metarhizium_humberi*_ESALQ_1638	CCTGTAATCC GGGAGTCCGT GGCGGTAAAG TCAACTGTAA GGCTTGTGTG TGTG------ ---------- ----CCGGGG CCCTGTAAGT CCCGACCAGG	1100
*Metarhizium_brunneum*_TYPE_ARSEF_2107	CCTGTAATCC GGGAGTCCGT GGTGGTAAAG TCAGCTATAA GGCTTGTGTG TGTGTGGGTG CGCGGGTGTG TGTGCCGGGG CCCTGTAAGT CCCGACCAGG	1100
*Metarhizium_brunneum*_ARSEF_1066	CCTGTAATCC GGGAGCCCGT GGTGGTAAAG TCAGCTATAA GGCTTGTGTG TGTGTGGGTG CGCGGGTGTG TGTGCCGGGG CCCTGTAAGT CCCGACCAGG	1100
*Metarhizium_brunneum*_ARSEF_1278	CCTGTAATCC GGGAGTCCGT GGTGGTAAAG TCAGCTATAA GGCTTGTGTG TGTG------ ---------- ----CCGGGG CCCTGTAAGT CCCGACCAGG	1100
*Metarhizium_brunneum*_ARSEF_3295	CCTGTAATCC GGGAGTCCGT GGTGGTAAAG TCAGCTATAA GGCTTGTGTG TGTGTGGGTG CGCGGGTGTG TGTGCCGGGG CCCTGTAAGT CCCGACCAGG	1100
*Metarhizium_brunneum*_ARSEF_3826	CCTGTAATCC GGGAGCCCGT GGTGGTAAAG TCAGCTATAA GGCTTGTGTG TGTGTGGGTG CGCGGGTGTG TGTGCCGGGG CCCTGTAAGT CCCGACCAGG	1100
*Metarhizium_brunneum*_ARSEF_4152	CCTATAATCC GGGAGTCCGT GGTGGTAAAA TCAGCTATAA AGCTTGTGTG TATG------ --------TG TGTGCCGGGG CCCTGTAAGT CCCGACCAGG	1100
*Metarhizium_brunneum*_ARSEF_4179	CCTATAATCC GGGAGTCCGT GGTGGTAAAA TCAGCTATAA AGCTTGTGTG TATG------ --------TG TGTGCCGGGG CCCTGTAAGT CCCGACCAGG	1100

```
mcar-IDF1                                  ---------- ---------- ---------- ---------- ---------- ---------- ---------- ---------- ---------- ----------  1200
mcar-IDR1                                  ---------- ---------- ---------- ---------- ---------- ---------- ---------- ---------- ---------- ----------  1200
Metarhizium_caribense_INISAV_LBM-30        AACCCTACAG GTCACGTGCC GATGTAATAA GACAGATAAG ATATACGGCA CGTGCTAATA CACGTGC--- ---------C AATACAAGT- ----AACGAT  1200
Metarhizium_caribense_INISAV_LBM-41        AACCCTACAG GTCACGTGCC GATGTAATAA GACAGATAAG ATATACGGCA CGTGCTAATA CACGTGC--- ---------C AATACAAGT- ----AACGAT  1200
Metarhizium_caribense_INISAV_LBM-42        AACCCTACAG GTCACGTGCC GATGTAATAA GACAGATAAG ATATACGGCA CGTGCTAATA CACGTGC--- ---------C AATACAAGT- ----AACGAT  1200
Metarhizium_caribense_ARSEF_1448           AACCCTACAG GTCACGTGCC GATATAATAA GACAGATAAG ATATACGGCA CGTGCTAATA CACGTGC--- ---------C AATACAAGT- ----AACGAT  1200
Metarhizium_caribense_ARSEF_5197           AACCCTACAG GTCACGTGCC GATGTAATAA GACAGATAAG ATATACGGCA CGTGCTAATA CACGTGC--- ---------C AATACAAGT- ----AACGAT  1200

Metarhizium_pingshaense_TYPE_CBS_257.90    AACCCTACAG GCCACGTGCC GATATAATAA GATAGATAAG GTATACGGCA CGTGCTAATA CACGTGC--- ---------C AATACAAGT- ----AACGAT  1200
Metarhizium_pingshaense_ARSEF_2231         AACCCTACAG GTCACGTGCC GATACAATAA GATAGATAAG GTATACGGCA CGTGCTAATA CACGTGC--- ---------C AATACAAGT- ----AACGAT  1200
Metarhizium_pingshaense_ARSEF_2809         AACCCCACAG GTCACGTGCC ---------- ---------- ---------- ------AATA CACGTGC--- ---------C AATATAAGT- ----AACGAT  1200
Metarhizium_pingshaense_ARSEF_3180         AACCCTACAG GTCACGTGCC GATATAATAA GACAGATAAG ATATACGGCA CGTGCTAATA CACGTGCTAA TACACGTGCC AATACAAGT- ----AACGAT  1200
Metarhizium_pingshaense_ARSEF_3210         AACCCTACAG GTCACGTGCC GATACAATAA GATAGATAAG GTATACGGCA CGTGCTAATA CACGTGC--- ---------C AATACAAGT- ----AACGAT  1200
Metarhizium_pingshaense_ARSEF_3604         AACTCTACAG GTCACGTGCC GATATAACAA GACAGATAAG ATCTACGGCA CGTGCTAATA CACGTGC--- ---------C AATACAAGT- ----AACGAT  1200
Metarhizium_pingshaense_ARSEF_4290         AACCCTACAG GTCACGTGCC GATATAATAA GACAGATAAG ATATACGGCA CGTGCTAATA CACGTGC--- ---------C AATACAAGT- ----AACGAT  1200
Metarhizium_pingshaense_ARSEF_4342         AACCCTACAG GTCACGTGCC GATATAATAA GACAGATAAG ATATACGGCA CGTGCTAATA CACGTGC--- ---------C AATACAAGT- ----AACGAT  1200
Metarhizium_pingshaense_ARSEF_7422         AACCCTACAG GTCACGTGCC GATATAATAA GACAGATAAG ATATACGGCA CGTGCTAATA CACGTGC--- ---------C AATACAAGT- ----AACGAT  1200
Metarhizium_pingshaense_ARSEF_7929         AACCCTACAG GTCACGTGCC GATACAATAA GATAGATAAG GTATACGGCA CGTGCTAATA CACGTGC--- ---------C AATACAAGT- ----AACGAT  1200
Metarhizium_pingshaense_ARSEF_9975         AACCCTACAG GTCACGTGCC GATACAATAA GATAGATAAG GTATACGGCA CGTGCTAATA CACGTGC--- ---------C AATACAAGT- ----AACGAT  1200

Metarhizium_robertsii_TYPE_ARSEF_2575      AACCCTACAG GTCACGTGCC GATACAATAA GACAGATAAG ATATACGGCA CGTGCTAATA CACGTGC--- ---------C AATACAAGT- ----GACGAT  1200
Metarhizium_robertsii_ARSEF_727            AACCCTACAG GTCACGTGCC GATACAATAA GACAGATAAG ATATACGGCA CGTGCTAATA CACGTGC--- ---------C AATACAAGT- ----GACGAT  1200
Metarhizium_robertsii_ARSEF_7501           AACCCTACAG GTCACGTGCC GATACAATAA GACAGATAAG ATATACGGCA CGTGCTAATA CACGTGC--- ---------C AATACAAGT- ----GACGAT  1200
Metarhizium_robertsii_ARSEF_8070           AACCCTACAG GTCACGTGCC GATACAATAA GACAGATAAG ATATACGGCA CGTGCTAATA CACGTGC--- ---------C AATACAAGT- ----GACGAT  1200
Metarhizium_robertsii_ARSEF_8685           AACCCTACAG GTCACGTGCC GATACAATAA GACAGATAAG ATATACGGCA CGTGCTAATA CACGTGC--- ---------C AATACAAGT- ----GACGAT  1200

Metarhizium_anisopliae_TYPE_ARSEF_7487     AACCCTACAG GTCACGTGCC GATACAATAA GACAGATAAG ATATGCGGCA CGTGCTAGTA CACGTGC--- ---------C AATACAAGT- ----GACGAT  1200
Metarhizium_anisopliae_ARSEF_798           AACCCTACAG GTTATGTGCC GATACAATAA GACAGATAAG ATATGCGGCA CGTGCTAGTA CACGTGC--- ---------C AATACAAGT- ----GACGAT  1200
Metarhizium_anisopliae_ARSEF_1080          AACCCTACAG GTTATGTGCC GATACAATAA GACAGATAAG ATATGCGGCA CGTGCTAGTA CACGTGC--- ---------C AATACAAGT- ----GACGAT  1200
Metarhizium_anisopliae_ARSEF_6347          AACCCTACAG GTTATGTGCC GATACAATAA GACAGATAAG ATATGCGGCA CGTGCTAGTA CACGTGC--- ---------C AATACAAGT- ----GACGAT  1200
Metarhizium_anisopliae_ARSEF_7450          AACCCTACAG GTCACGTGCC GATACAATAA GACAGATAAG ATATGCGGCA CGTGCTAGTA CACGTGC--- ---------C AATACAAGT- ----GACGAT  1200

Metarhizium_humberi_ESALQ_1638             AACCCTACAG GTCACGTGCC GATACGATAA GACAGATAAG ATATACGGCA CGTGCTAGTA CACGTGC--- ---------C AATACAAGT- ----GACGAT  1200

Metarhizium_brunneum_TYPE_ARSEF_2107       AACCCTACAG GTCACGTGCC GACACAGTAA GACAGATAAG ATATACGGCA CGTGTTAGTA CACGTGC--- ---------C AATACAAGT- ----GACGAT  1200
Metarhizium_brunneum_ARSEF_1066            AACCCTACAG GTCACGTGCC GACACAGTAA GACAGATAAG ATATACGGCA GGTGTTAGTA CACGTGC--- ---------C AATACAAGT- ----GACGAT  1200
Metarhizium_brunneum_ARSEF_1278            AACCCTGCAG GTCACGTGCC GACACAGTAA GACAGATAAG ATATACGGCA CGTGTTAGTA CACGTGC--- ---------C AATACAAGT- ----GACGAT  1200
Metarhizium_brunneum_ARSEF_3295            AACCCTACAG GTCACGTGCC GACACAGTAA GACAGATAAG ATATACGGCA CGTGTTAGTA CACGTGC--- ---------C AATACAAGT- ----GACGAT  1200
Metarhizium_brunneum_ARSEF_3826            AACCCTACAG GTCACGTGCC GACACAGTAA GACAGATAAG ATATACGGCA GGTGTTAGTA CACGTGC--- ---------C AATACAAGT- ----GACGAT  1200
Metarhizium_brunneum_ARSEF_4152            AACCTTACAG GTCACGTGCC GATACGATAA GACAGATAAG ATATACAGCA CGTGCTAGTA CACGTGC--- ---------C AGTATAAGTA ACGAGACGAT  1200
Metarhizium_brunneum_ARSEF_4179            AACCTTACAG GTCACGTGCC GATACGATAA GACAGATAAG ATATACAGCA CGTGCTAGTA CACGTGC--- ---------C AGTATAAGTA ACGAGACGAT  1200
```

mcar-IDF1	---------- ---------- ---------- ---------- ---------- ---------- ---------- ---------- ---------- ----------	1300
mcar-IDR1	---------- ---------- ---------- ---------- ---------- ---------- ---------- ---------- ---------- ----------	1300
*Metarhizium_caribense*_INISAV_LBM-30	AAGATCAACA AGATACCTGC ACGTGCCTAG TCACGGAGAC TTACACGTGT CCCGCTGCCA AGATCCCCGA GGT------- --TGCCGACC TCGCCGACTA	1300
*Metarhizium_caribense*_INISAV_LBM-41	AAGATCAACA AGATACCTGC ACGTGCCTAG TCACGGAGAC TTACACGTGT CCCGCTGCCA AGATCCCCGA GGT------- --TGCCGACC TCGCCGACTA	1300
*Metarhizium_caribense*_INISAV_LBM-42	AAGATCAACA AGATACCTGC ACGTGCCTAG TCACGGAGAC TTACACGTGT CCCGCTGCCA AGATCCCCGA GGT------- --TGCCGACC TCGCCGACTA	1300
*Metarhizium_caribense*_ARSEF_1448	AAGATCAACA AGATACCTGC ACGTGCCTAG TCACGGAGAC TTACACGTGT CCCGCTGCCA AGATCCCCGA GGT------- --TGCCGACC TCGCCGACTA	1300
*Metarhizium_caribense*_ARSEF_5197	AAGATCAACA AGATACCTGC ACGTGCCTAG TCACGGAGAC TTACACGTGT CCCGCTGCCA AGATCCCCGA GGT------- --TGCCGACC TCGCCGACTA	1300
*Metarhizium_pingshaense*_TYPE_CBS_257.90	AAGATCAACA AGATACCTGC ACGTGCCTAG TCACGGAGAC TTACACGTGT CCCGCTGCCA AGATCCCCGA GGT------- --TGCCGACC TCGCCGACTA	1300
*Metarhizium_pingshaense*_ARSEF_2231	AAGATCAACA AGATACCTGC ACGTGCCTAG TCACGGAGAC TTACACGTGT CCCGCTGCCA AGATCCCCGA GGT------- --TGCCGACC TCGCCGACTA	1300
*Metarhizium_pingshaense*_ARSEF_2809	AAGATCAACA AGATACCTGC ACGTGCCTAG TCACGGAGAC TTACACGTGT CCCGCTGCCA AGATCCCCGA GGT------- --TGCCGACC TCGCCGACCA	1300
*Metarhizium_pingshaense*_ARSEF_3180	AAGATCAACA AGATACCTGC ACGTGCCTAG TCACGGAGAC TTACACGTGT CCCGCTGCCA AGATCCCCGA GAT------- --TGCCGACC TCGCCGACTA	1300
*Metarhizium_pingshaense*_ARSEF_3210	AAGATCAACA AGATACCTGC ACGTGCCTAG TCACGGAGAC TTACACGTGT CCCGCTGCCA AGATCCCCGA GGT------- --TGCCGACC TCGCCGACTA	1300
*Metarhizium_pingshaense*_ARSEF_3604	AAGATCAACA AGATACCTGC ACGTGCCTAG TCACGGAGAC TTACACGTGT CCCGCTGCCA AGATCCCCGA GGT------- --TGCCGACC TCGCCGACTA	1300
*Metarhizium_pingshaense*_ARSEF_4290	AAGATCAACA AGATACCTGC ACGTGCCTAG TCACGGAGAC TTACACGTGT CCCGCTGCCA AGATCCCCGA GAT------- --TGCCGACC TCGCCGACTA	1300
*Metarhizium_pingshaense*_ARSEF_4342	AAGATCAACA AGATACCTGC ACGTGCCTAG TCACGGAGAC TTACACGTGT CCCGCTGCCA AGATCCCCGA GAT------- --TGCCGACC TCGCCGACTA	1300
*Metarhizium_pingshaense*_ARSEF_7422	AAGATCAACA AGATACCTGC ACGTGCCTAG TCACGGAGAC TTACACGTGT CCCGCTGCCA AGATCCCCGA GGT------- --TGCCGACC TCGCCGACTA	1300
*Metarhizium_pingshaense*_ARSEF_7929	AAGATCAACA AGATACCTGC ACGTGCCTAG TCACGGAGAC TTACACGTGT CCCGCTGCCA AGATCCCCGA GGT------- --TGCCGACC TCGCCGACTA	1300
*Metarhizium_pingshaense*_ARSEF_9975	AAGATCAACA AGATACCTGC ACGTGCCTAG TCACGGAGAC TTACACGTGT CCCGCTGCCA AGATCCCCGA GGT------- --TGCCGACC TCGCCGACTA	1300
*Metarhizium_robertsii*_TYPE_ARSEF_2575	AAGATCAACA AGATACCTGC ACGTGCCTAG TCACGGAGAC TTACACGTGT CCCGCTGCCA AGATCCCCGA GAT------- --TGCCGACC TCGCCGACTA	1300
*Metarhizium_robertsii*_ARSEF_727	AAGATCAACA AGATACCTGC ACGTGCCTAG TCACGGAGAC TTACACGTGT CCCGCTGCCA AGATCCCCGA GAT------- --TGCCGACC TCGCCGACTA	1300
*Metarhizium_robertsii*_ARSEF_7501	AAGATCAACA AGATACCTGC ACGTGCCTAG TCACGGAGAC TTACACGTGT CCCGCTGCCA AGATCCCCGA GAT------- --TGCCGACC TCGCCGACTA	1300
*Metarhizium_robertsii*_ARSEF_8070	AAGATCAACA AGATACCTGC ACGTGCCTAG TCACGGAGAC TTACACGTGT CCCGCTGCCA AGATCCCCGA GAT------- --TGCCGACC TCGCCGACTA	1300
*Metarhizium_robertsii*_ARSEF_8685	AAGATCAACA AGATACCTGC ACGTGCCTAG TCACGGAGAC TTACACGTGT CCCGCTGCCA AGATCCCCGA GAT------- --TGCCGACC TCGCCGACTA	1300
*Metarhizium_anisopliae*_TYPE_ARSEF_7487	AAGATCGATA AGATACCTGC ACGTGCCTAG TCACGGAGAC TTACACGTGT TCCGCTGCCA AGGTCCCCGA GGTCCCCGCG ATTGCCGACC TCGCCGACTA	1300
*Metarhizium_anisopliae*_ARSEF_798	AAGATCGATA AGATACCTGC ACGTGCCTAG TCACGGAGAC TTACACGTGT TCCGCTGCCA AGGTCCCCGA GGTCCCCGCG ATTGCCGACC TCGCCGACTA	1300
*Metarhizium_anisopliae*_ARSEF_1080	AAGATCGATA AGATACCTGC ACGTGCCTAG TCACGGAGAC TTACACGTGT TCCGCTGCCA AGGTCCCCGA GGTCCCCGCG ATTGCCGACC TCGCCGACTA	1300
*Metarhizium_anisopliae*_ARSEF_6347	AAGATCGATA AGATACCTGC ACGTGCCTAG TCACGGAGAC TTACACGTGT TCCGCTGCCA AGGTCCCCGA GGTCCCCGCG ATTGCCGACC TCGCCGACTA	1300
*Metarhizium_anisopliae*_ARSEF_7450	AAGATCGATA AGATACCTGC ACGTGCCTAG TCACGGAGAC TTACACGTGT TCCGCTGCCA AGGTCCCCGA GGTCCCCGCG ATTGCCGACC TCGCCGACTA	1300
*Metarhizium_humberi*_ESALQ_1638	AAGATCGACA AGATACCTGC ACGTGCCTAG TCACGGAGAC TTACACGTGT TCCGCTGCCA AGGTCCCCGA GGTCCCCGAG ATTGCCGACC TCGCCGACTA	1300
*Metarhizium_brunneum*_TYPE_ARSEF_2107	AAGATCGATA AGATACCTGC ACGTGCCCAG TCACGGAGAC TTACACGTGT CCCGCTGCCA AGGTCCCCGA GGTCCCCGAG GTTGCCGACC TCGCCGACTA	1300
*Metarhizium_brunneum*_ARSEF_1066	AAGATCGATA AGATACCTGC ACGTGCCCAG TCACGGAGAC TTACACGTGT CCCGCTGCCA AGGTCCCCGA GGT------- --TGCCGACC TCGCCGACTA	1300
*Metarhizium_brunneum*_ARSEF_1278	AAGATCGATA AGATACCTGC ACGTGCCCAG TCACGGAGAC TTACACGTGT CCCGCTGCCA AGGTCCCCGA GGTCCCCGAG GTTGCCGACC TCGCCGACTA	1300
*Metarhizium_brunneum*_ARSEF_3295	AAGATCGATA AGATACCTGC AC-------- ---------- ---------- ---------- ---------- ---------- ---------- ----------	1300
*Metarhizium_brunneum*_ARSEF_3826	AAGATCGATA AGATACCTGC ACGTGCCCAG TCACGGAGAC TTACACGTGT CCCGCTGCCA AGGTCCCCGA GGT------- --TGCCGACC TCGCCGACTA	1300
*Metarhizium_brunneum*_ARSEF_4152	AAGATCGATA AGATACCTGC ACGTGCCTAG TCACGGAGAC TTACACGTGT CCCGCCGCCA AGGTCCCCAA GGTCCCCGAG GTTGCCGACC TCGCCGACTA	1300
*Metarhizium_brunneum*_ARSEF_4179	AAGATCGATA AGATACCTGC ACGTGCCTAG TCACGGAGAC TTACACGTGT CCCGCCGCCA AGGTCCCCAA GGTCCCCGAG GTTGCCGACC TCGCCGACTA	1300

```
mcar-IDF1                                ---------- ---------- ---------- ---------- ---------- ---------- ---------- ---------- ---------- ----------  1400
mcar-IDR1                                ---------- ---------- ---------- ---------- ---------- ---------- ---------- ---------- ---------- ----------  1400
Metarhizium_caribense_INISAV_LBM-30      CTCCTGGTGT AATGTGCCGG TGTAGTTGTA TATAATTTCT TACTAGTCTC AATAATAGTA CACTTGTTTA TCGCGATGCA CTCACTTGTG --------GT  1400
Metarhizium_caribense_INISAV_LBM-41      CTCCTGGTGT AATGTGCCGG TGTAGTTGTA TATAATTTCT TACTAGTCTC AATAATAGTA CACTTGTTTA TCGCGATGCA CTCACTTGTG --------GT  1400
Metarhizium_caribense_INISAV_LBM-42      CTCCTGGTGT AATGTGCCGG TGTAGTTGTA TATAATTTCT TACTAGTCTC AATAATAGTA CACTTGTTTA TCGCGATGCA CTCACTTGTG --------GT  1400
Metarhizium_caribense_ARSEF_1448         CTCCTGGTGT AATGTGCCGG TGTAGTTGTA TATAATTTCT TACTAGTCTC AATAATAGTA CACTTGTTTA TCGCGATGCA CTCACTTGTG --------GT  1400
Metarhizium_caribense_ARSEF_5197         CTCCTGGTGT AATGTGCCGG TGTAGTTGTA TATAATTTCT TACTAGTCTC AATAATAGTA CACTTGTTTA TCGCGATGCA CTCACTTGTG --------GT  1400

Metarhizium_pingshaense_TYPE_CBS_257.90  CTCCTGGTGT AATGTGCCGG TGTAGTTATA TATAATTTCT TACTAGTCTC AATAATAGTA CACTCGTTTA TCGCGATGCA CTCACTTGTG --------GT  1400
Metarhizium_pingshaense_ARSEF_2231       CTCCTGGTGT AATGTGCCGG TGTAGTTATA TATAATTTCT TACTAGTCTC AATAATAGTA AAATCGTTTA TCGCGATGCA CTCACTTGTG --------GT  1400
Metarhizium_pingshaense_ARSEF_2809       CTCCTGGTGT AATGTGCCGG TGTAGTTATA TATAATTTCT TACTAGTCTC AATAATAGTA CACTC----- ---------- ---ACTTGTG --------GT  1400
Metarhizium_pingshaense_ARSEF_3180       CTCCTGGTGT AATGTGCCGG TGTAGTTATA TATAATTTCT TACTAGTCTC AATAATAGTA CACTCGTTTA TCGCGATGCA CTCACTTGTG ----------  1400
Metarhizium_pingshaense_ARSEF_3210       CTCCTGGTGT AATGTGCCGG TGTAGTTATA TATAATTTCT TACTAGTCTC AATAATAGTA CACTCGTTTA TCGCGATGCA CTCACTTGTG --------GT  1400
Metarhizium_pingshaense_ARSEF_3604       CTCCTGGTGT AATGTGCCGG TGTAGTTATA TATAATTTCT TACTAGTCTC AATAATAGTA CACTCGTTTA TCGCGATGCA CTCACTTGTG --------GT  1400
Metarhizium_pingshaense_ARSEF_4290       CTCCTGGTGT AATGTGCCGG TGTAGTTATA TATAATTTCT TACTAGTCTC AATAATAGTA CACTCGTTTA TCGCGATGCA CTCACTTGTG ----------  1400
Metarhizium_pingshaense_ARSEF_4342       CTCCTGGTGT AATGTGCCGG TGTAGTTATA TATAATTTCT TACTAGTCTC AATAATAGTA CACTCGTTTA TCGCGATGCA CTCACTTGTG ----------  1400
Metarhizium_pingshaense_ARSEF_7422       CTCCTGGCGT AATGTGCCGG TGTAGTTATA TATAATTTCT TACTAGTCTC AATAATAGTA CACCCGTTTA TCGCGATGCA CTCACTTGTG ----------  1400
Metarhizium_pingshaense_ARSEF_7929       CTCCTGGTGT AATGTGCCGG TGTAGTTATA TATAATTTCT TACTAGTCTC AATAATAGTA CACTCGTTTA TCGCGATGCA CTCACTTGTG --------GT  1400
Metarhizium_pingshaense_ARSEF_9975       CTCCTGGTGT AATGTGCCGG TGTAGTTATA TATAATTTCT TACTAGTCTC AATAATAGTA CACTCGTTTA TCGCGATGCA CTCACTTGTG --------GT  1400

Metarhizium_robertsii_TYPE_ARSEF_2575    CTCTTGGTGT AATGTGCCGG TGT------- ---AATTTCT TGCTAGTCTC AATAATAGTA CACTCGTTTA TCGCGATGCA CTTACTTGTA ----------  1400
Metarhizium_robertsii_ARSEF_727          CTCTTGGTGT AATGTGCCGG TGT------- ---AATTTCT TACTAGTCTC AATAATAGTA CACTCGTTTA TCGCGATGCA CTTACTTGTA ----------  1400
Metarhizium_robertsii_ARSEF_7501         CTCTTGGTGT AATGTGC--- ---------- ---------- ---------- ---------- ---------- ---------- ---------- ----------  1400
Metarhizium_robertsii_ARSEF_8070         CTCTTGGTGT AATGTGCCGG TGT------- ---AATTTCT TACTAGTCTC AATAATAGTA CACTCGTTTA TCGCGATGCA CTTACTTGTA ----------  1400
Metarhizium_robertsii_ARSEF_8685         CTCTTGGTGT AATGTGCCGG TGT------- ---AATTTCT TACTAGTCTC AATAATAGTA CACTCGTTTA TCGCGATGCA CTTACTTGTA ----------  1400

Metarhizium_anisopliae_TYPE_ARSEF_7487   CTCCTGGTGT AATATACCGG TGTAGTTGTA TATAATTTCT TACTAGTCTT AATAGTAGTA CACTTGTTTA TCGCGATACA CTTACTTGTG GTGCGGCTGT  1400
Metarhizium_anisopliae_ARSEF_798         CACCTGGTGT AATATGCCGG TGTAGTTGTG TATAATTTCT TACTAGTCTT AATAGTAGTA CACTTGTTTA TCGCGATACA CTTACTTGTG --------GT  1400
Metarhizium_anisopliae_ARSEF_1080        CTCCTGGTGT AATATGCCGG TGTAGTTGTG TATAATTTCT TACTAGTCTT AATAGTAGTA CACTTGTTTA TCGCGATACA CTTACTTGTG --------GT  1400
Metarhizium_anisopliae_ARSEF_6347        CTCCTGGTGT AATATGCCGG TGTAGTTGTG TATAATTTCT TACTAGTCTT AATAGTAGTA CACTTGTTTA TCGCGATACA CTTACTTGTG --------GT  1400
Metarhizium_anisopliae_ARSEF_7450        CTCCTGGTGT AATATACCGG TGTAGTTGTA TATAATTTCT TACTAGTCTT AATAGTAGTA CACTTGTTTA TCGCGATACA CTTACTTGTG GTGCGGCTGT  1400

Metarhizium_humberi_ESALQ_1638           CTCCTGGTGT AATGTGCCGG TGTAGTTATA TATAATTTCT TACTAGTCTT AATAATAGTA CACTTGTTTA TCGCGATACA CTTACTTGTG GTGCGGCTGT  1400

Metarhizium_brunneum_TYPE_ARSEF_2107     CTCCTGGTGT AATGTGCCGG TGCAGTTGCA TATAATTCCT TACTAGTCTC AATAATAGTA CACTCGTTTA TCGCGATGCA CTCACTTGTG --------GT  1400
Metarhizium_brunneum_ARSEF_1066          CTCCTGGTGT AATGTGCCGG TGCA------ ---------- ---------- ---------- ---------- ---------- ---------- ----------  1400
Metarhizium_brunneum_ARSEF_1278          CTCCTGGTGT AATGTGCCGG TGCAGTTGCA TATAATTCCT TACTAGTCTC AATAATAGTA CACTCGTTTA TCGCGATGCA CTCACTTGTG --------GT  1400
Metarhizium_brunneum_ARSEF_3295          ---------- ---------- ---------- ---------- ---------- ---------- ---------- ---------- ---------- ----------  1400
Metarhizium_brunneum_ARSEF_3826          CTCCTGGTGT AATGTGCCGG TGCA------ ---------- ---------- ---------- ---------- ---------- ---------- ----------  1400
Metarhizium_brunneum_ARSEF_4152          CTCCTGGTGT AATATGCCGG TACAGTTGCA TATAATTCCC TACTAGTCTC AATAATAGTA CACTCGTTTA TCGCGATGCA CTTACTTGTG --------GT  1400
Metarhizium_brunneum_ARSEF_4179          CTCCTGGTGT AATATGCCGG TACAGTTGCA TATAATTCCC TACTAGTCTC AATAATAGTA CACTCGTTTA TCGCGATGCA CTTACTTGTG --------GT  1400
```

mcar-IDF1	---------- ---------- ---------- ---------- ---------- ---------- ---------- ---------- ---------- ----------	1500
mcar-IDR1	---------- ---------- ---------- ---------- ---------- ---------- ---------- ---------- ---------- ----------	1500
*Metarhizium_caribense*_INISAV_LBM-30	GCGGCTGTGC GGCTGTGCTG ACGTACTTGT GGTGGTGCAC TTACTCTACA ATAGTGCTGG TACTTGCGGT CACATACAAG TTCCTAGTAT TTCGGCGTAG	1500
*Metarhizium_caribense*_INISAV_LBM-41	GCGGCTGTGC GGCTGTGCTG ACGTACTTGT GGTGGTGCAC TTACTCTACA ATAGTGCTGG TACTTGCGGT CACATACAAG TTCCTAGTAT TTCGGCGTAG	1500
*Metarhizium_caribense*_INISAV_LBM-42	GCGGCTGTGC GGCTGTGCTG ACGTACTTGT GGTGGTGCAC TTACTCTACA ATAGTGCTGG TACTTGCGGT CACATACAAG TTCCTAGTAT TTCGGCGTAG	1500
*Metarhizium_caribense*_ARSEF_1448	GCGGCTGTGC GGCTGTGCTG ACGTACTTGT GGTGGTGCAC TTACTCTACA ATAGTGCTGG TACTTGCGGT CACATACAAG TTCCTAGTAT TTCGGCGTAG	1500
*Metarhizium_caribense*_ARSEF_5197	GCGGCTGTGC GGCTGTGCTG ACGTACTTGT GGTGGTGCAC TTACTCTACA ATAGTGCTGG TACTTGCGGT CACATACAAG TTCCTAGTAT TTCGGCGTAG	1500
*Metarhizium_pingshaense*_TYPE_CBS_257.90	GCGGGTGTGC GGGTGTGCTG ACGTACTTGT GGTGGTGCAC TTACTCTACA ATAGTGCTGG TACTTGCGGT CATATACAAG TTCCTAGTAT TTTGGCGTAG	1500
*Metarhizium_pingshaense*_ARSEF_2231	GCGGGTGTGC GGGTGTGCTG ACGTACTTGT GGTGGTGCAC TTACTCTACA ATAGTGCTGG TACTTGCGGT CATATACAAG TTCCTAGTAT TTTGGCGTAG	1500
*Metarhizium_pingshaense*_ARSEF_2809	GCGGCTGTGC GGCTGTGCTG ACGTGCTTGT GGTGGTGCAC TTACTCTACA ATAGTGCTGG TACTTGCGGT CATATACAAG TTCCTAGTAT TTTGGCGTAG	1500
*Metarhizium_pingshaense*_ARSEF_3180	---------- ---------- ---------- -GTGGTGCAC TTACTCTACA ATAGTGCTGG TACTTGCGGT CATATACAAG TTCCTAGTAT TTTGGCGTAG	1500
*Metarhizium_pingshaense*_ARSEF_3210	GCGGGTGTGC GGGTGTGCTG ACGTACTTGT GGTGGTGCAC TTACTCTACA ATAGTGCTGG TACTTGCGGT CATATACAAG TTCCTAGTAT TTTGGCGTAG	1500
*Metarhizium_pingshaense*_ARSEF_3604	GCGGCTGTGC GGCTGTGCTG ACGTACTTGT GGTGGTGCAC TTACTCTACA ATAGTGCTGG TACTTGCGGT CATATACAAG TTCCTAGTAT TTTGGCGTAG	1500
*Metarhizium_pingshaense*_ARSEF_4290	---------- ---------- ---------- -GTGGTGCAC TTACTCTACA ATAGTGCTGG TACTTGCGGT CATATACAAG TTCCTAGTAT TTTGGCGTAG	1500
*Metarhizium_pingshaense*_ARSEF_4342	---------- ---------- ---------- -GTGGTGCAC TTACTCTACA ATAGTGCTGG TACTTGCGGT CATATACAAG TTCCTAGTAT TTTGGCGTAG	1500
*Metarhizium_pingshaense*_ARSEF_7422	---------- ---------- ---------- -GTGGTGCAC TTACTCTACA ATAGTGCTGG TACTTGCGGT CATATACAAG TTCCTAGTAT TTTGGCGTAG	1500
*Metarhizium_pingshaense*_ARSEF_7929	GCGGGTGTGC GGGTGTGCTG ACGTACTTGT GGTGGTGCAC TTACTCTACA ATAGTGCTGG TACTTGCGGT CATATACAAG TTCCTAGTAT TTTGGCGTAG	1500
*Metarhizium_pingshaense*_ARSEF_9975	GCGGGTGTGC GGGTGTGCTG ACGTACTTGT GGTGGTGCAC TTACTCTACA ATAGTGCTGG TACTTGCGGT CATATACAAG TTCCTAGTAT TTTGGCGTAG	1500
*Metarhizium_robertsii*_TYPE_ARSEF_2575	------GTGC GGTTGTGCTG ACGTACTTGT GGTGGTGTAC TTACTCTATA ATAGTGCTGG TACTTGCGGT CACATACAAG TTCCTAGTAT TTTGGCGTAG	1500
*Metarhizium_robertsii*_ARSEF_727	------GTGC GGTTGTGCTG ACGTACTTGT GGTGGTGTAC TTACTCTATA ATAGTGCTGG TACTTGCGGT CACATACAAG TTCCTAGTAT TTTGGCGTAG	1500
*Metarhizium_robertsii*_ARSEF_7501	---------- ---------- ---------- ---------- ---------- -------TGG TACTTGCGGT CACATACAAG TTCCTAGTAT TTTGGCGTAG	1500
*Metarhizium_robertsii*_ARSEF_8070	------GTGC GGTTGTGCTG ACGTACTTGT GGTGGTGTAC TTACTCTATA ATAGTGCTGG TACTTGCGGT CACATACAAG TTCCTAGTAT TTTGGCGTAG	1500
*Metarhizium_robertsii*_ARSEF_8685	------GTGC GGTTGTGCTG ACGTACTTGT GGTGGTGTAC TTACTCTATA ATAGTGCTGG TACTTGCGGT CACATACAAG TTCCTAGTAT TTTGGCGTAG	1500
*Metarhizium_anisopliae*_TYPE_ARSEF_7487	GCGGCTGTGC GGCTGTGCTG ACGTACTTGT AGTGGTGCAC TTACTCTACA ATAGTGCTGG TACTTACGGT CGCACACAAA TCCCTAGTAT TTTGGTGTAG	1500
*Metarhizium_anisopliae*_ARSEF_798	GCGGCTGTGC GGCTGTGCTG ATGTACTTGT AGTGGTGCAC TTACTCTACA ATAGTGCTGG TACTTACGGT CGCACACAAA TCCCTAGTAT TTTGGTGTAG	1500
*Metarhizium_anisopliae*_ARSEF_1080	GCGGCTGTGC GGCTGTGCTG ATGTACTTGT AGTGGTGCAC TTACTCTACA ATAGTGCTGG TACTTACGGT CGCACACAAA TCCCTAGTAT TTTGGTGTAG	1500
*Metarhizium_anisopliae*_ARSEF_6347	GCGGCTGTGC GGCTGTGCTG ATGTACTTGT AGTGGTGCAC TTACTCTACA ATAGTGCTGG TACTTACGGT CGCACACAAA TCCCTAGTAT TTTGGTGTAG	1500
*Metarhizium_anisopliae*_ARSEF_7450	GCGGCTGTGC GGCTGTGCTG ACGTACTTGT AGTGGTGCAC TTACTCTACA ATAGTGCTGG TACTTACGGT CGCACACAAA TCCCTAGTAT TTTGGTGTAG	1500
*Metarhizium_humberi*_ESALQ_1638	GCGGCTGTGC GGCTGTGCTG ACGTACTTGT AGTGGTGTAC TTACTCTACA ATAGTGCTGG TACTTACGGT CGCACACAAA TCCCTAGTAT TTTAGTGTAG	1500
*Metarhizium_brunneum*_TYPE_ARSEF_2107	ACGGCTGTGC GGCTGTGCTG ACGTACTTGT GGT------- ---------- ---------- ---------- ---------- ---------- ----------	1500
*Metarhizium_brunneum*_ARSEF_1066	---------- ---------- ---------- ---------- ---------- ---------- ---------- ---------- ---------- ----------	1500
*Metarhizium_brunneum*_ARSEF_1278	ACGGCTGTGC GGCTGTGCTG ACGTACTT-- ---------- ---------- ---------- ---------- ---------- ---------- ----------	1500
*Metarhizium_brunneum*_ARSEF_3295	---------- ---------- ---------- ---------- ---------- ---------- ---------- ---------- ---------- ----------	1500
*Metarhizium_brunneum*_ARSEF_3826	---------- ---------- ---------- ---------- ---------- ---------- ---------- ---------- ---------- ----------	1500
*Metarhizium_brunneum*_ARSEF_4152	GCGGCTGTGC GGCTGTGCTG ACGTACTTGT GGTAGTGCAC TTACTCTACA ATAGTGCTGG TACTTGCGGT CACATACAAT TTCCCAGTAT TTTGGCGTAG	1500
*Metarhizium_brunneum*_ARSEF_4179	GCGGCTGTGC GGCTGTGCTG ACGTACTTGT GGTAGTGCAC TTACTCTACA ATAGTGCTGG TACTTGCGGT CACATACAAT TTCCCAGTAT TTTGGCGTAG	1500

```
mcar-IDF1                                  ---------- ---------- ---------- ---------- ---------- ---------- ---------- ---------- ---------- ----------  1600
mcar-IDR1                                  ---------- ---------- ---------- ---------- ---------- ---------- ---------- ---------- ---------- ----------  1600
Metarhizium_caribense_INISAV_LBM-30        CTGTGGTCGT GGT------- ---------- ---------- ---------- ---------- ---------- ---------- ---------- -------GGA  1600
Metarhizium_caribense_INISAV_LBM-41        CTGTGGTCGT GGT------- ---------- ---------- ---------- ---------- ---------- ---------- ---------- -------GGA  1600
Metarhizium_caribense_INISAV_LBM-42        CTGTGGTCGT GGT------- ---------- ---------- ---------- ---------- ---------- ---------- ---------- -------GGA  1600
Metarhizium_caribense_ARSEF_1448           TTGTGGTCGT GGT------- ---------- ---------- ---------- ---------- ---------- ---------- ---------- -CGTGGTGGA  1600
Metarhizium_caribense_ARSEF_5197           CTGTGGTCGT GGT------- ---------- ---------- ---------- ---------- ---------- ---------- ---------- -------GGA  1600

Metarhizium_pingshaense_TYPE_CBS_257.90    TTGTGGCCGT GGTCGTGCAG TTACAGCAGT GTACTTACCC TGTAGTACTG CTGGCACCGG CCACCGTCGC GTGCACGCGG CTGCCGTCGG CCGTGGTAGA  1600
Metarhizium_pingshaense_ARSEF_2231         TTGTGGCCGT GGTCGTGCAG TTACAGCAGT GTACTTACCC TGTAGTACTG CTGGCACCGG CCACCGTCGC GTGCACGCGG CTGCCGTCGG CCGTGGTAGA  1600
Metarhizium_pingshaense_ARSEF_2809         TTGTGGTCGT GGTCGTGCAG TTACAGCAGT GTACTTACCC TGTAGTACTG CTGGCACCGG CCACCGTCGC GTGCACGCGG CTGCCGTCGG CCGTGGTAGA  1600
Metarhizium_pingshaense_ARSEF_3180         TTGTGGTCGT GGTCGTGCAG TTACAGCAGT GTACTTACCC TGTAGTACTG CTGGCACCGG CCACCGTCGC GTGCACGCGG CTGCCGTCGG CCGTGGTAGA  1600
Metarhizium_pingshaense_ARSEF_3210         TTGTGGCCGT GGTCGTGCAG TTACAGCAGT GTACTTACCC TGTAGTACTG CTGGCACCGG CCACCGTCGC GTGCACGCGG CTGCCGTCGG CCGTGGTAGA  1600
Metarhizium_pingshaense_ARSEF_3604         TTGTGGTCGT GGTCGTGCAG TTACAGCAGT GTACTTACCC TGTAGTACTG CTGGCACCGG CCA------- ---------- ---CCGTCGG CCGTGGTAGA  1600
Metarhizium_pingshaense_ARSEF_4290         TTGTGGTCGT GGTCGTGCAG TTACAGCAGT GTACTTACCC TGTAGTACTG CTGGCACCGG CCACCGTCGC GTGCACGCGG CTGCCGTCGG CCGTGGTAGA  1600
Metarhizium_pingshaense_ARSEF_4342         TTGTGGTCGT GGTCGTGCAG TTACAGCAGT GTACTTACCC TGTAGTACTG CTGGCACCGG CCACCGTCGC GTGCACGCGG CTGCCGTCGG CCGTGGTAGA  1600
Metarhizium_pingshaense_ARSEF_7422         TTGTGGTCGT GGTCGTGCAG TTACAGCAGT GTACTTACCC TGTAGTACTG CTGGCACCGG CCACCGTCGC GTGCACGCGG CTGCCGTCGG CCGTGGTAGA  1600
Metarhizium_pingshaense_ARSEF_7929         TTGTGGCCGT GGTCGTGCAG TTACAGCAGT GTACTTACCC TGTAGTACTG CTGGCACCGG CCACCGTCGC GTGCACGCGG CTGCCGTCGG CCGTGGTAGA  1600
Metarhizium_pingshaense_ARSEF_9975         TTGTGGCCGT GGTCGTGCAG TTACAGCAGT GTACTTACCC TGTAGTACTG CTGGCACCGG CCACCGTCGC GTGCACGCGG CTGCCGTCGG CCGTGGTAGA  1600

Metarhizium_robertsii_TYPE_ARSEF_2575      TTGTGGTCGT GGTCGTGCAG TTACAGCAGT GCACTTACCC TGTAGTACTG CTGGCACCGG CCACCGTCGC GTGCACGCGG CTGCCGTCGG CCGTGGTGGA  1600
Metarhizium_robertsii_ARSEF_727            TTGTGGTCGT GGTCGTGCAG TTACAGCAGT GCACTTACCC TGTAGTACTG CTGGCACCGG CCACCGTCGC GTGCACGCGG CTGCCGTCGG CCGTGGTGGA  1600
Metarhizium_robertsii_ARSEF_7501           TTGTGGTCGT GGTCGTGCAG TTACAGCAGT GCACTTACCC TGTAGTACTG CTGGCACCGG CCACCGTCGC GTGCACGCGG CTGCCGTCGG CCGTGGTGGA  1600
Metarhizium_robertsii_ARSEF_8070           TTGTGGTCGT GGTCGTGCAG TTACAGCAGT GCACTTACCC TGTAGTACTG CTGGCACCGG CCACCGTCGC GTGCACGCGG CTGCCGTCGG CCGTGGTGGA  1600
Metarhizium_robertsii_ARSEF_8685           TTGTGGTCGT GGTCGTGCAG TTACAGCAGT GCACTTACCC TGTAGTACTG CTGGCACCGG CCACCGTCGC GTGCACGCGG CTGCCGTCGG CCGTGGTGGA  1600

Metarhizium_anisopliae_TYPE_ARSEF_7487     TTGTGATCGT GGTGGTGCAG TTACAGCAGT GCACTTACCC TGTAGTACTG CTGGCACCGG CCA------- ---------- ---CCGTCGG CCGTGGCGGA  1600
Metarhizium_anisopliae_ARSEF_798           TTGTGGTCGT GGTGGTGCAG TTACAGCAGT GCACTTACCC TGTAGTACTG CTGGCACCGG CCA------- ---------- ---CCGTCGG CCGTGGCGGA  1600
Metarhizium_anisopliae_ARSEF_1080          TTGTGGTCGT GGTGGTGCAG TTACACCAGT GCACTTACCC TGTAGTACTG CTGGCACCGG CCA------- ---------- ---CCGTCGG CCGTGGCGGA  1600
Metarhizium_anisopliae_ARSEF_6347          TTGTGGTCGT GGTGGTGCAG TTACAGCAGT GCACTTACCC TGTAGTACTG CTGGCACCGG CCA------- ---------- ---CCGTCGG CCGTGGCGGA  1600
Metarhizium_anisopliae_ARSEF_7450          TTGTGATCGT GGTGGTGCAG TTACAGCAGT GCACTTACCC TGTAGTACTG CTGGCACCGG CCA------- ---------- ---CCGTCGG CCGTGGCGGA  1600

Metarhizium_humberi_ESALQ_1638             TTGTGGTCGT GGTGGTGCAG TTACAGCAGT GTACTTACCC TGTAGTACTG CTGGCGCCGG CCA------- ---------- ---CCGTCGG CCGTGGCGGA  1600

Metarhizium_brunneum_TYPE_ARSEF_2107       ---------- ---------- ---------- ---------- ---------- ---------- ---------- ---------- ---------- -------GGA  1600
Metarhizium_brunneum_ARSEF_1066            ---------- ---------- ---------- ---------- ---------- ---------- TCACCGTCGC GTGCACGCGG CTGCCGTCGG CCGTGGTGGA  1600
Metarhizium_brunneum_ARSEF_1278            --------GT GGTGGTGGAG TTACAGCAGT GCACTTACCC TGTAGCACTG CTGGTACCGG CCACCGTCGC GTGCACGCGG CTGCCGTCGG CCGTGGTGGA  1600
Metarhizium_brunneum_ARSEF_3295            ---------- ---------- ---------- ----TTACCC TGTAGCACTG CTGGTACCGG CCACCGCCGC GTGCACGCGG CTGCCGTCGG CCGTGGTGGA  1600
Metarhizium_brunneum_ARSEF_3826            ---------- ---------- ---------- ---------- ---------- ---------- TCACCGTCGC GTGCACGCGG CTGCCGTCGG CCGTGGTGGA  1600
Metarhizium_brunneum_ARSEF_4152            TTGTGGTCGT GGTGGTGCAG TTACAGCAGT GCACTTACCC TGTAGTACTG CTGGTACCGG CCACCGTCGC GTGCGCGCGG CTGCCGTCGG CCGTGGTGGA  1600
Metarhizium_brunneum_ARSEF_4179            TTGTGGTCGT GGTGGTGCAG TTACAGCAGT GCACTTACCC TGTAGTACTG CTGGTACCGG CCACCGTCGC GTGCGCGCGG CTGCCGTCGG CCGTGGTGGA  1600
```

mcar-IDF1	---------- ---------- ---------- ---------- ---------- ---------- ---------- ---------- ---------- ----------	1700
mcar-IDR1	---------- ---------- ---------- ---------- ---------- ---------- ---------- ---------- ---------- ----------	1700
*Metarhizium_caribense*_INISAV_LBM-30	GGGTTACCCG GGACCACGGT GCATTTTTCC -GTTTTTTCC ATTTTTTTCA AAATCGACGA CCAAAAGGTT GGTAGGTCTG GGTTGCCTTG CTGGCCAATA	1700
*Metarhizium_caribense*_INISAV_LBM-41	GGGTTACCCG GGACCACGGT GCATTTTTCC -GTTTTTTCC ATTTTTTTCA AAATCGACGA CCAAAAGGTT GGTAGGTCTG GGTTGCCTTG CTGGCCAATA	1700
*Metarhizium_caribense*_INISAV_LBM-42	GGGTTACCCG GGACCACGGT GCATTTTTCC -GTTTTTTCC ATTTTTTTCA AAATCGACGA CCAAAAGGTT GGTAGGTCTG GGTTGCCTTG CTGGCCAATA	1700
*Metarhizium_caribense*_ARSEF_1448	GGGTTACCCG GGACCACGGT GCATTTTTCC -GTTTTTTCC ATTTTTTTCA AAATCGACGA CCAAAAGGTT GGTAGGTCTG GGTTGCCTTG CTGGCCAATA	1700
*Metarhizium_caribense*_ARSEF_5197	GGGTTACCCG GGACCACGGT GCATTTTTCC -GTTTTTTCC ATTTTTTTCA AAATCGACGA CCAAAAGGTT GGTAGGTCTG GGTTGCCTTG CTGGCCAATA	1700
*Metarhizium_pingshaense*_TYPE_CBS_257.90	GGGTTACCCG GGACCACGGT GCATTTTTCC -GTTTTTTCC ATTTTTT-CA AAATCGACGA CCAAAAAGTT GGTAGGTCTG GGTTGCCTTG CTGGCCAATA	1700
*Metarhizium_pingshaense*_ARSEF_2231	GGGTTACCCG GGACCACGGT GCATTTTTCC -GTTTTTTCC ATTTTTT-CA AAATCGACGA CCAAAAAGTT GGTAGGTCTG GGTTGCCTTG CTGGCCAATA	1700
*Metarhizium_pingshaense*_ARSEF_2809	GGGTTACCCG GGACCACGGT GCATTTTTCC -GTTTTTTCC ATTTTTT-CA AAATCGACGA CCAAAAAGTT GGTAGGTCTG GGTTGCCTTG CTGGCCAATA	1700
*Metarhizium_pingshaense*_ARSEF_3180	GGGTTACCCG GGACCACGGT GCATTTTTCC -GTTTTTTCC ATTTTTT-CA AAATCGACGA CCAAAAAGTT GGTAGGTCTG GGTTGCCTTG CTGGCCAATA	1700
*Metarhizium_pingshaense*_ARSEF_3210	GGGTTACCCG GGACCACGGT GCATTTTTCC -GTTTTTTCC ATTTTTT-CA AAATCGACGA CCAAAAAGTT GGTAGGTCTG GGTTGCCTTG CTGGCCAATA	1700
*Metarhizium_pingshaense*_ARSEF_3604	GGGTTACCCG GGACCACGGT GCATTTTTCC -GTTTTTTCC ATTTCTT-CA AAATCGACGA CCAAAAAGTT GGTAGGTCTG GGTTGCCTTG CTGGCCAATA	1700
*Metarhizium_pingshaense*_ARSEF_4290	GGGTTACCCG GGACCACGGT GCATTTTTCC -GTTTTTTCC ATTTTTT-CA AAATCGACGA CCAAAAAGTT GGTAGGTCTG GGTTGCCTTG CTGGCCAATA	1700
*Metarhizium_pingshaense*_ARSEF_4342	GGGTTACCCG GGACCACGGT GCATTTTTCC -GTTTTTTCC ATTTTTT-CA AAATCGACGA CCAAAAAGTT GGTAGGTCTG GGTTGCCTTG CTGGCCAATA	1700
*Metarhizium_pingshaense*_ARSEF_7422	GGGTTACCCG GGACCACGGT GCATTTTTCC -GTTTTTTCC ATTTTTT-CA AAATCGACGA CCAAAAAGTT GGTAGGTCTG GGTTGCCTTG CTGGCCAATA	1700
*Metarhizium_pingshaense*_ARSEF_7929	GGGTTACCCG GGACCACGGT GCATTTTTCC -GTTTTTTCC ATTTTTT-CA AAATCGACGA CCAAAAAGTT GGTAGGTCTG GGTTGCCTTG CTGGCCAATA	1700
*Metarhizium_pingshaense*_ARSEF_9975	GGGTTACCCG GGACCACGGT GCATTTTTCC -GTTTTTTCC ATTTTTT-CA AAATCGACGA CCAAAAAGTT GGTAGGTCTG GGTTGCCTTG CTGGCCAATA	1700
*Metarhizium_robertsii*_TYPE_ARSEF_2575	GGGTTACCCG GGACCACGGT GCATTTTTCC -GTTTTTTTC ATTTTTTTCA AAATCGACGA CCAAAAAGTT GGTAGGTCTG GGTTGCCTTG CTGGCCAATA	1700
*Metarhizium_robertsii*_ARSEF_727	GGGTTACCCG GGACCACGGT GCATTTTTCC -GTTTTTTTC ATTTTTTTCA AAATCGACGA CCAAAAAGTT GGTAGGTCTG GGTTGCCTTG CTGGCCAATA	1700
*Metarhizium_robertsii*_ARSEF_7501	GGGTTACCCG GGACCACGGT GCATTTTTCC -GTTTTTTTC ATTTTTTTCA AAATCGACGA CCAAAAAGTT GGTAGGTCTG GGTTGCCTTG CTGGCCAATA	1700
*Metarhizium_robertsii*_ARSEF_8070	GGGTTACCCG GGACCACGGT GCATTTTTCC -GTTTTTTTC ATTTTTTTCA AAATCGACGA CCAAAAAGTT GGTAGGTCTG GGTTGCCTTG CTGGCCAATA	1700
*Metarhizium_robertsii*_ARSEF_8685	GGGTTACCCG GGACCACGGT GCATTTTTCC -GTTTTTTTC ATTTTTTTCA AAATCGACGA CCAAAAAGTT GGTAGGTCTG GGTTGCCTTG CTGGCCAATA	1700
*Metarhizium_anisopliae*_TYPE_ARSEF_7487	GGGTTACCCG GGACCACAGT GCATTTTTCC -GTTTTTTCC ATTTTTT-CA AAATCGACGA CCAAAAAGTT GGTAGGTCTG GGTTGCCTTG CTGGCCAATA	1700
*Metarhizium_anisopliae*_ARSEF_798	GGGTTACCCG GGACCACAGT GCATTTTTCC -GTTTTTTCC ATTTTTT-CA AAATCGACGA CCAAAAAGTT GGTAGGTCTG GGTTGCCTTG CTGGCCAATA	1700
*Metarhizium_anisopliae*_ARSEF_1080	GGGTTACCCG GGACCACAGT GCATTTTTCC -GTTTTTTCC ATTTTTT-CA AAATCGACGA CCAAAAAGTT GGTAGGTCTG GGTTGCCTTG CTGGCCAATA	1700
*Metarhizium_anisopliae*_ARSEF_6347	GGGTTACCCG GGACCACAGT GCATTTTTCC -GTTTTTTCC ATTTTTT-CA AAATCGACGA CCAAAAAGTT GGTAGGTCTG GGTTGCCTTG CTGGCCAATA	1700
*Metarhizium_anisopliae*_ARSEF_7450	GGGTTACCCG GGACCACAGT GCATTTTTCC -GTTTTTTCC ATTTTTT-CA AAATCGACGA CCAAAAAGTT GGTAGGTCTG GGTTGCCTTG CTGGCCAATA	1700
*Metarhizium_humberi*_ESALQ_1638	GGGTTACCCG GGACCACAGT GCATTTTTCC -GTTTTTTCC ATTTTTT-CA AAATCGACGA CCAAAAAGTT GGTAGGTCTG GGTTGCCTTG CTGGCCAATA	1700
*Metarhizium_brunneum*_TYPE_ARSEF_2107	GGGTTACCCG GGACCACGGT GCATTTTTCC -GTTTTTTCC ATTTTTT-CA AAATCGACGA CCAAAAGGTT GGCAGGTCTG GGTTGCCTTG CTGGCCAATA	1700
*Metarhizium_brunneum*_ARSEF_1066	GGGTTACCCG GGACCACGGT GCATTTTTCC -GTTTTTTCC ATTTTTT-CA AAATCGACGA CCAAAAGGTT GGTAGGTCTG GGTTGCCTTG CTGGCCAATA	1700
*Metarhizium_brunneum*_ARSEF_1278	GGGTTACCCG GGACCACGGT GCATTTTTCC -GTTTTTTCC ATTTTTT-CA AAATCGACGA CCAAAAGGTT GGTAGGTCTG GGTTGCCTTG CTGGCCAATA	1700
*Metarhizium_brunneum*_ARSEF_3295	GGGTTACCCG GGACCACGGT GCATTTTTCC TTTTTTTTCC ATTTTTT-CA AAATCGACGA CCAAAAGGTT GGTAGGTCTG GGTTGCCTGG CCGGCCAATA	1700
*Metarhizium_brunneum*_ARSEF_3826	GGGTTACCCG GGACCACGGT GCATTTTTCC -GTTTTTTCC ATTTTTT-CA AAATCGACGA CCAAAAGGTT GGTAGGTCTG GGTTGCCTTG CTGGCCAATA	1700
*Metarhizium_brunneum*_ARSEF_4152	GGGCTACCCG GGACCACGGT GCAATTTTCC -TTTTTTTCC ATTTTTT-CA AAATCGACGA CCAAAAGGTT GGTAGGTCTG GGTTGCCTTG CTGGCCAATA	1700
*Metarhizium_brunneum*_ARSEF_4179	GGGCTACCCG GGACCACGGT GCAATTTTCC -TTTTTTTCC ATTTTTT-CA AAATCGACGA CCAAAAGGTT GGTAGGTCTG GGTTGCCTTG CTGGCCAATA	1700

mcar-IDF1	---------- ---------- ---------- ---------- ---------- ---------- ---------- ---------- ---------- ----------	1800
mcar-IDR1	---------- ---------- ---------- ---------- ---------- ---------- ---------- ---------- ---------- ----------	1800
*Metarhizium_caribense*_INISAV_LBM-30	GAGATTTGTA TGGCGCTACT ATAGGGTAGG ---------- ---------- ---------- ---------- ---------- ---------- ----------	1800
*Metarhizium_caribense*_INISAV_LBM-41	GAGATTTGTA TGGCGCTACT ATAGGGTAGG ---------- ---------- ---------- ---------- ---------- ---------- ----------	1800
*Metarhizium_caribense*_INISAV_LBM-42	GAGATTTGTA TGGCGCTACT ATAGGGTAGG ---------- ---------- ---------- ---------- ---------- ---------- ----------	1800
*Metarhizium_caribense*_ARSEF_1448	GAGATTTGTA TGGCGCTACT ATAGGGTAGG ---------- ---------- ---------- ---------- ---------- ---------- ----------	1800
*Metarhizium_caribense*_ARSEF_5197	GAGATTTGTA TGGCGCTACT ATAGGGTAGG ---------- ---------- ---------- ---------- ---------- ---------- ----------	1800
*Metarhizium_pingshaense*_TYPE_CBS_257.90	GAGATTTGTA TGGCGCTACT ATAGGGTAGG ---------- ---------- ---------- ---------- ---------- ---------- ----------	1800
*Metarhizium_pingshaense*_ARSEF_2231	GAGATTTGTA TGGCGCTACT ATAGGGTAGG ---------- ---------- ---------- ---------- ---------- ---------- ----------	1800
*Metarhizium_pingshaense*_ARSEF_2809	GAGATTTGTA TGGCGCTACT ATAGGGTAGG ---------- ---------- ---------- ---------- ---------- ---------- ----------	1800
*Metarhizium_pingshaense*_ARSEF_3180	GAGATTTGTA TGGCGCTACT ATAGGGTAGG ---------- ---------- ---------- ---------- ---------- ---------- ----------	1800
*Metarhizium_pingshaense*_ARSEF_3210	GAGATTTGTA TGGCGCTACT ATAGGGTAGG ---------- ---------- ---------- ---------- ---------- ---------- ----------	1800
*Metarhizium_pingshaense*_ARSEF_3604	GAGATTTGTA TGGCGCTACT ATAGGGTAGG ---------- ---------- ---------- ---------- ---------- ---------- ----------	1800
*Metarhizium_pingshaense*_ARSEF_4290	GAGATTTGTA TGGCGCTACT ATAGGGTAGG ---------- ---------- ---------- ---------- ---------- ---------- ----------	1800
*Metarhizium_pingshaense*_ARSEF_4342	GAGATTTGTA TGGCGCTACT ATAGGGTAGG ---------- ---------- ---------- ---------- ---------- ---------- ----------	1800
*Metarhizium_pingshaense*_ARSEF_7422	CAGATTTGTA TGGCGCTACT ATAGGGTAAG ---------- ---------- ---------- ---------- ---------- ---------- ----------	1800
*Metarhizium_pingshaense*_ARSEF_7929	GAGATTTGTA TGGCGCTACT ATAGGGTAGG ---------- ---------- ---------- ---------- ---------- ---------- ----------	1800
*Metarhizium_pingshaense*_ARSEF_9975	GAGATTTGTA TGGCGCTACT ATAGGGTAGG ---------- ---------- ---------- ---------- ---------- ---------- ----------	1800
*Metarhizium_robertsii*_TYPE_ARSEF_2575	GAGATTTGTA TGGCGCTACT ATAGGGTAGG ---------- ---------- ---------- ---------- ---------- ---------- ----------	1800
*Metarhizium_robertsii*_ARSEF_727	GAGATTTGTA TGGCGCTACT ATAGGGTAGG ---------- ---------- ---------- ---------- ---------- ---------- ----------	1800
*Metarhizium_robertsii*_ARSEF_7501	GAGATTTGTA TGGCGCTACT ATAGGGTAGG ---------- ---------- ---------- ---------- ---------- ---------- ----------	1800
*Metarhizium_robertsii*_ARSEF_8070	CAGATTTGTA TGGCGCTACT ATAGGGTAGG ---------- ---------- ---------- ---------- ---------- ---------- ----------	1800
*Metarhizium_robertsii*_ARSEF_8685	GAGATTTGTA TGGCGCTACT ATAGGGTAGG ---------- ---------- ---------- ---------- ---------- ---------- ----------	1800
*Metarhizium_anisopliae*_TYPE_ARSEF_7487	GAGATTTGTA T--------- --AGGGTAGG CCGGGGCAGG TACTTGCAGT CGCATACAAA TTCTCTAGTA ATTTGATGTA CCTCCGCGCG AATGGCAGTT	1800
*Metarhizium_anisopliae*_ARSEF_798	GAGATTTGTA TGGCGCTACT ATAGGGTAGG CTG-TGCCGG TACTTGCTGT CGCATACAAA TTCCCTAGTA ACTGGAAGTA CCTC------ ----GCAGTT	1800
*Metarhizium_anisopliae*_ARSEF_1080	GAGATTTGTA TGGCGCTACT ATAGGGTAGG CTG-TGCCGG TACTTGCTGT CGCATACAAA TTCCCTAGTA ACTGGAAGTA CCTC------ ----GCAGTT	1800
*Metarhizium_anisopliae*_ARSEF_6347	GAGATTTGTA TGGCGCTACT ATAGGGTAGG CTG-TGCCGG TACTTGCTGT CGCATACAAA TTCCCTAGTA ACTGGAAGTA CCTC------ ----GCAGTT	1800
*Metarhizium_anisopliae*_ARSEF_7450	GAGATTTGTA T--------- --AGGGTAGG CCGGGGCAGG TACTTGCAGT CGCATACAAA TTCTCTAGTA ATTTGATGTA CCTCCGCGCG AATGGCAGTT	1800
*Metarhizium_humberi*_ESALQ_1638	GAGATTGGTA T--------- --AGGGTAGG CTGGTACCGG TACTTGCAGT CGCATACAAA TTCTCTAGTA ATTTGATGTA CCTCCGCGCG AATGGCAGTT	1800
*Metarhizium_brunneum*_TYPE_ARSEF_2107	GAGATTTGTA CGGCGCTACT ATAGGGTAGG ---------- ---------- ---------- ---------- ---------- ---------- ----------	1800
*Metarhizium_brunneum*_ARSEF_1066	GAGATTTGTA CGGCGCTACT ATAGGGTAGG ---------- ---------- ---------- ---------- ---------- ---------- ----------	1800
*Metarhizium_brunneum*_ARSEF_1278	GAGATTTGTA CGGCGCTACT ATAGGGTAGG ---------- ---------- ---------- ---------- ---------- ---------- ----------	1800
*Metarhizium_brunneum*_ARSEF_3295	GAGATTTGTA CGGCGCTACT ATAGGGTAGG ---------- ---------- ---------- ---------- ---------- ---------- ----------	1800
*Metarhizium_brunneum*_ARSEF_3826	GAGATTTGTA CGGCGCTACT ATAGGGTAGG ---------- ---------- ---------- ---------- ---------- ---------- ----------	1800
*Metarhizium_brunneum*_ARSEF_4152	GAGATTTGTA CGGCGCTACT ATAGGGTAGG ---------- ---------- ---------- ---------- ---------- ---------- ----------	1800
*Metarhizium_brunneum*_ARSEF_4179	GAGATTTGTA CGGCGCTACT ATAGGGTAGG ---------- ---------- ---------- ---------- ---------- ---------- ----------	1800

mcar-IDF1	---------- ---------- ---------- ---------- ---------- ---------- ---------- ---------- ---------- ---	1893
mcar-IDR1	---------- ---------- ---------- ---------- ---------- ---------- ---------- ---------- ---------- ---	1893
*Metarhizium_caribense*_INISAV_LBM-30	---------- --TATAGGGT AGGCTG-TGC CGGTACTTGC GGTCGCACAC AAAT-CCCTA GCAGCCTGGA GTACCCGCGC GCGCGGGCAA CTT	1893
*Metarhizium_caribense*_INISAV_LBM-41	---------- --TATAGGGT AGGCTG-TGC CGGTACTTGC GGTCGCACAC AAAT-CCCTA GCAGCCTGGA GTACCCGCGC GCGCGGGCAA CTT	1893
*Metarhizium_caribense*_INISAV_LBM-42	---------- --TATAGGGT AGGCTG-TGC CGGTACTTGC GGTCGCACAC AAAT-CCCTA GCAGCCTGGA GTACCCGCGC GCGCGGGCAA CTT	1893
*Metarhizium_caribense*_ARSEF_1448	---------- --TATAGGGT AGGCTG-TGC CGGTACTTGC GGTCGCACAC AAAT-CCCTA GCAGCCTGGA GTACCCGCGC GCGCGGGCAA CTT	1893
*Metarhizium_caribense*_ARSEF_5197	---------- --TATAGGGT AGGCTG-TGC CGGTACTTGC GGTCGCACAC AAAT-CCCTA GCAGCCTGGA GTACCCGCGC GCGCGGGCAA CTT	1893
*Metarhizium_pingshaense*_TYPE_CBS_257.90	---------- --TATAGGGT AGGCTG-TAC CGGTACTTGC GGTCGCACAC AAAT-CCCTA GCAGCCTGGA GTACCCCCGC GCGCGGGCAA CTT	1893
*Metarhizium_pingshaense*_ARSEF_2231	---------- --TATAGGGT AGGCTG-TAC CGGTACTTGC GGTCGCACAC AAAT-CCCTA GCAGCCTGGA GTACCCCCGC GCGCGGGCAA CTT	1893
*Metarhizium_pingshaense*_ARSEF_2809	---------- --TACAGGGT AGGCTG-TAC CGGTACTTGC GGTCGCACAC AAAT-CCCTA GCAGCCTGGA GTACCCCCGC GCGCGGGCAA CTT	1893
*Metarhizium_pingshaense*_ARSEF_3180	---------- --TGTAGGGT AGGCTG-TAC CGGTACTTGC GGTCGCACAC AAAT-CCCTA GCAGCCTGGA GTACCCCCGC GCGCGGGCAA CTT	1893
*Metarhizium_pingshaense*_ARSEF_3210	---------- --TATAGGGT AGGCTG-TAC CGGTACTTGC GGTCGCACAC AAAT-CCCTA GCAGCCTGGA GTACCCCCGC GCGCGGGCAA CTT	1893
*Metarhizium_pingshaense*_ARSEF_3604	---------- --TATAGGGT AGGCTG-TAC CGGTACTTGC GGTCGCACAC AAAT-CCCTA GCAGCCTGGA GTACCCCCGC GCGCGGGCAA CTT	1893
*Metarhizium_pingshaense*_ARSEF_4290	---------- --TGTAGGGT AGGCTG-TAC CGGTACTTGC GGTCGCACAC AAAT-CCCTA GCAGCCTGGA GTACCCCCGC GCGCGGGCAA CTT	1893
*Metarhizium_pingshaense*_ARSEF_4342	---------- --TGTAGGGT AGGCTG-TAC CGGTACTTGC GGTCGCACAC AAAT-CCCTA GCAGCCTGGA GTACCCCCGC GCGCGGGCAA CTT	1893
*Metarhizium_pingshaense*_ARSEF_7422	---------- --CATAGGGT AGGCTG-TAC CGGTACTTGC GGTCGCACAC AAAT-CCCTA GCAGCCTGGA GTACCCCCGC GCGCGGGCAA CTT	1893
*Metarhizium_pingshaense*_ARSEF_7929	---------- --TATAGGGT AGGCTG-TAC CGGTACTTGC GGTCGCACAC AAAT-CCCTA GCAGCCTGGA GTACCCCCGC GCGCGGGCAA CTT	1893
*Metarhizium_pingshaense*_ARSEF_9975	---------- --TATAGGGT AGGCTG-TAC CGGTACTTGC GGTCGCACAC AAAT-CCCTA GCAGCCTGGA GTACCCCCGC GCGCGGGCAA CTT	1893
*Metarhizium_robertsii*_TYPE_ARSEF_2575	---------- --TATAGGGT AGGCTG-TGC CGGTACTTGC GGTCGCACAC AAAT-CCCTA GCAGCCTGAA GTACCCCCGC GCGCGGGCAA CTT	1893
*Metarhizium_robertsii*_ARSEF_727	---------- --TATAGGGT AGGCTG-TGC CGGTACTTGC GGTCGCACAC AAAT-CCCTA GCAGCCTGAA GTACCCCCGC GCGCGGGCAA CTT	1893
*Metarhizium_robertsii*_ARSEF_7501	---------- --TATAGGGT AGGCTG-TGC CGGTACTTGC GGTCGCACAC AAAT-CCCTA GCAGCCTGAA GTACCCCCGC GCGCGGGCAA CTT	1893
*Metarhizium_robertsii*_ARSEF_8070	---------- --TATAGGGT AGGCTG-TGC CGGTACTTGC GGTCGCACAC AAAT-CCCTA GCAGCCTGAA GTACCCCCGC GCGCGGGCAA CTT	1893
*Metarhizium_robertsii*_ARSEF_8685	---------- --TATAGGGT AGGCTG-TGC CGGTACTTGC GGTCGCACAC AAAT-CCCTA GCAGCCTGAA GTACCCCCGC GCGCGGGCAA CTT	1893
*Metarhizium_anisopliae*_TYPE_ARSEF_7487	TGGCAGGGTA GGTACCGGGT AGGCTGGTGC AGGTACTTGC AGTTGCATAC AAATTCTCTA GTAACTGGAA GTACCCCCGC GCGCGGGCAA CTT	1893
*Metarhizium_anisopliae*_ARSEF_798	TGGTAGGGCA GGTACCGGGT AGGCTG-TGC CGGTACTTGC TGTCGCATAC AAATTCCCTA GTAACTGGAA GTACCCCCGC GCGCGGGCAA CTT	1893
*Metarhizium_anisopliae*_ARSEF_1080	TGGTAGGGCA GGTACCGGGT AGGCTG-TGC CGGTACTTGC TGTCGCATAC AAATTCCCTA GTAACTGGAA GTACCCCCGC GCGCGGGCAA CTT	1893
*Metarhizium_anisopliae*_ARSEF_6347	TGGTAGGGCA GGTACCGGGT AGGCTG-TGC CGGTACTTGC TGTCGCATAC AAATTCCCTA GTAACTGGAA GTACCCCCGC GCGCGGGCAA CTT	1893
*Metarhizium_anisopliae*_ARSEF_7450	TGGCAGGGTA GGTACCGGGT AGGCTGGTGC AGGTACTTGC AGTTGCATAC AAATTCTCTA GTAACTGGAA GTACCCCCGC GCGCGGGCAA CTT	1893
*Metarhizium_humberi*_ESALQ_1638	TGGTAGGGTA GGTACCGGGT AGGCTGGTGC AGGTACTTGC AGTTGCATAC AGATTCCCTA GTAACTGGAA GTACCCCCGC GCGCGGGCAA CTT	1893
*Metarhizium_brunneum*_TYPE_ARSEF_2107	---------- --TACAGGGT AGGCTG-TGC CGGTACTTGC GGTCGCACAC AAAT-CCCTA GCAGCCTGAA GTACCCCCGC GCGCGGGCAA CTT	1893
*Metarhizium_brunneum*_ARSEF_1066	---------- --TACAGGGT AGGCTG-TGC CGGTACTTGC GGTCGCACAC AAAT-CCCTA GCAGCCTGAA GTACCCCCGC GCGCGGGCAA CTT	1893
*Metarhizium_brunneum*_ARSEF_1278	---------- --TACAGGGT AGGCTG-TGC CGGTACTTGC GGTCGCACAC AAAT-CCCTA GCAGCCCGAA GTACCCCCGC GCGCGGGCAA GTT	1893
*Metarhizium_brunneum*_ARSEF_3295	---------- --TACAGGGT AGGCTG-TGC CGGTACTTGC GGTCGCACAC AAAT-CCCTA GCAGCCTGAA GTACCCCCGC GCGCGGGCAG CTT	1893
*Metarhizium_brunneum*_ARSEF_3826	---------- --TACAGGGT AGGCTG-TGT CGGTACTTGC GGTCGCACAC AAAT-CCCTA GCAGCCTGAA GTACCCCCGC GCGCGGGCAA CTT	1893
*Metarhizium_brunneum*_ARSEF_4152	---------- --TACAGGGT AGGCTG-TGC CGGTACTTGC GGTCGCACAC AAAT-CCCTA GCAGCCTGAA GTACCCCCGC GCGCGGGCAG CTT	1893
*Metarhizium_brunneum*_ARSEF_4179	---------- --TACAGGGT AGGCTG-TGC CGGTACTTGC GGTCGCACAC AAAT-CCCTA GCAGCCTGAA GTACCCCCGC GCGCGGGCAG CTT	1893
