# Supplementary material for: Metarhizium caribense sp. nov., a Novel Species of Entomopathogenic Metarhizium Fungi Associated with Weevils Impairing Coffee, Sugar Cane and Sweet Potato Cultivation
Source: J Fungi (Basel). 2024 Aug 28;10(9):612. doi: 10.3390/jof10090612 (PMC11433170; doi:10.3390/jof10090612)
Supplement: Supplementary file 1 [file jof-10-00612-s001.zip › Suppl Figure S8.pdf]

Supplementary Figure S8. Alignment of 28Si4 intron sequences used for diagnostic primer design.

```
Meta28Si4long-F1              ---------- ---------- ---------- ---------- ---------- ---------- ---------- ---------- ---------- ----------   100
Mcar28Si4long-F1              ---------- ---------- ---------- ---------- ---------- ---------- ---------- ---------- ---------- ----------   100
M_caribense_INISAV_LBM-30     AGATTGTGTC TCATGTTCTA TACATAAGCG TATCTCACCG TCTGTATTAT GAGTGTTTCC ACGAGTAATT CAGACGTCTT CGGAGTTGGC AGAGGGAAAC   100
M_caribense_INISAV_LBM-41     AGATTGTGTC TCATGTTCTA TACATAAGCG TATCTCACCG TCTGTATTAT GAGTGTTTCC ACGAGTAATT CAGACGTCTT CGGAGTTGGC AGAGGGAAAC   100
M_caribense_INISAV_LBM-42     AGATTGTGTC TCATGTTCTA TACATAAGCG TATCTCACCG TCTGTATTAT GAGTGTTTCC ACGAGTAATT CAGACGTCTT CGGAGTTGGC AGAGGGAAAC   100
M_caribense_ARSEF_5197        AGATTGTGTC TCATGTTCTA TACATAAGCG TATCTCACCG TCTGTATTAT GAGTGTTTCC ACGAGTAATT CAGACGTCTT CGGAGTTGGC AGAGGGAAAC   100
Mmaj28Si4long-F1              ---------- ---------- ---------- ---------- ---------- ---------- ---------- ---------- ---------- ----------   100
Metarhizium_sp_EAMa_00/09-Su  AGATTGTGTC TCATGTTCTA TACATAAGCG TATCTCACCG TCTGTATTAT GAGTGTTTCC ACGAGTAATT CAGACGTCTT CGGAGTTGGC AGAGGGAAAC   100
Metarhizium_sp_EAMa_01/16-Su  AGATTGTGTC TCATGTTCTA TACATAAGCG TATCTCACCG TCTGTATTAT GAGTGTTTCC ACGAGTAATT CAGACGTCTT CGGAGTTGGC AGAGGGAAAC   100
Metarhizium_sp_EAMa_01/94-Su  AGATTGTGTC TCATGTTCTA TACATAAGCG TATCTCACCG TCTGTATTAT GAGTGTTTCC ACGAGTAATT CAGACGTCTT CGGAGTTGGC AGAGGGAAAC   100

Meta28Si4long-F1              ---------- ---------- ---------- ---------- ---------- ---------- ---------- ---------- ---------- ----------   200
Mcar28Si4long-F1              ---------- ---------- ---------- ---------- ---------- ---------- ---------- ---------- ---------- ----------   200
M_caribense_INISAV_LBM-30     ACCTCTACAG CCTCCTTCGG GTCTTCAAGC ATCGGAATGG TTCTTGGGGC GACTTTGGGA GGTTCTTGGA GTTTATCTTC ACTGATTCTT CCCAGGGAGG   200
M_caribense_INISAV_LBM-41     ACCTCTACAG CCTCCTTCGG GTCTTCAAGC ATCGGAATGG TTCTTGGGGC GACTTTGGGA GGTTCTTGGA GTTTATCTTC ACTGATTCTT CCCAGGGAGG   200
M_caribense_INISAV_LBM-42     ACCTCTACAG CCTCCTTCGG GTCTTCAAGC ATCGGAATGG TTCTTGGGGC GACTTTGGGA GGTTCTTGGA GTTTATCTTC ACTGATTCTT CCCAGGGAGG   200
M_caribense_ARSEF_5197        ACCTCTACAG CCTCCTTCGG GTCTTCAAGC ATCGGAATGG TTCTTGGGGC GACTTTGGGA GGTTCTTGGA GTTTATCTTC ACTGATTCTT CCCAGGGAGG   200
Mmaj28Si4long-F1              ---------- ---------- ---------- ---------- ---------- ---------- ---------- ---------- ---------- ----------   200
Metarhizium_sp_EAMa_00/09-Su  ACCTCTACAG CCTCCTTCGG GTCTTCAAGC ATCGGAATGG TTCTTGGGGC GACTTTGGGA GGTTCTTGGA GTTTATCTTC ACTGATCCTT CCCAGGGAGG   200
Metarhizium_sp_EAMa_01/16-Su  ACCTCTACAG CCTCCTTCGG GTCTTCAAGC ATCGGAATGG TTCTTGGGGC GACTTTGGGA GGTTCTTGGA GTTTATCTTC ACTGATCCTT CCCAGGGAGG   200
Metarhizium_sp_EAMa_01/94-Su  ACCTCTACAG CCTCCTTCGG GTCTTCAAGC ATCGGAATGG TTCTTGGGGC GACTTTGGGA GGTTCTTGGA GTTTATCTTC ACTGATCCTT CCCAGGGAGG   200

Meta28Si4long-F1              ---------- ---------- ---------- ---------- ---------- ---------- ---------- ---------- ---------- ----------   300
Mcar28Si4long-F1              ---------- ---------- ---------- ---------- ---------- ---------- ---------- ---------- ---------- ----------   300
M_caribense_INISAV_LBM-30     TGGTGGTCTG TCAGTAAGTC ATATTACTTG CCGTCACAAC TGTGGTGGTA AGCATTATGA GGCGCTGGCG GCAGCGGCCG TTCATCCTGA GAACTACCCA   300
M_caribense_INISAV_LBM-41     TGGTGGTCTG TCAGTAAGTC ATATTACTTG CCGTCACAAC TGTGGTGGTA AGCATTATGA GGCGCTGGCG GCAGCGGCCG TTCATCCTGA GAACTACCCA   300
M_caribense_INISAV_LBM-42     TGGTGGTCTG TCAGTAAGTC ATATTACTTG CCGTCACAAC TGTGGTGGTA AGCATTATGA GGCGCTGGCG GCAGCGGCCG TTCATCCTGA GAACTACCCA   300
M_caribense_ARSEF_5197        TGGTGGTCTG TCAGTAAGTC ATATTACTTG CCGTCACAAC TGTGGTGGTA AGCATTATGA GGCGCTGGCG GCAGCGGCCG TTCATCCTGA GAACTACCCA   300
Mmaj28Si4long-F1              ---------- ---------- ---------- ---------- ---------- ---------- ---------- ---------- ---------- ----------   300
Metarhizium_sp_EAMa_00/09-Su  TGGTGGTCTG TCAGTAAGTC ATATTACTTG CCGTCACAAC TGTGGTGGTA AGCATTATGA GGCGCTGGCG GCAGCGGCCG TTCATCCTGA GAACTACCCA   300
Metarhizium_sp_EAMa_01/16-Su  TGGTGGTCTG TCAGTAAGTC ATATTACTTG CCGTCACAAC TGTGGTGGTA AGCATTATGA GGCGCTGGCG GCAGCGGCCG TTCATCCTGA GAACTACCCA   300
Metarhizium_sp_EAMa_01/94-Su  TGGTGGTCTG TCAGTAAGTC ATATTACTTG CCGTCACAAC TGTGGTGGTA AGCATTATGA GGCGCTGGCG GCAGCGGCCG TTCATCCTGA GAACTACCCA   300

Meta28Si4long-F1              ---------- ---------- ---------- ---------- ---------- ---------- ---------- ---------- ---------- ----------   400
Mcar28Si4long-F1              ---------- ---------- ---------- ---------- ---------- ---------- ---------- ---------- ---------- ----------   400
M_caribense_INISAV_LBM-30     ATGTTTGACC ACGAACGGTA CAGGCGCCCG TGCGTCAGCA CTGGTACCTT TGAGGATGGG AAGAACCCTG GTCTTGTAGT AGCAGATCTC TGTGAAAAAG   400
M_caribense_INISAV_LBM-41     ATGTTTGACC ACGAACGGTA CAGGCGCCCG TGCGTCAGCA CTGGTACCTT TGAGGATGGG AAGAACCCTG GTCTTGTAGT AGCAGATCTC TGTGAAAAAG   400
M_caribense_INISAV_LBM-42     ATGTTTGACC ACGAACGGTA CAGGCGCCCG TGCGTCAGCA CTGGTACCTT TGAGGATGGG AAGAACCCTG GTCTTGTAGT AGCAGATCTC TGTGAAAAAG   400
M_caribense_ARSEF_5197        ATGTTTGACC ACGAACGGTA CAGGCGCCCG TGCGTCAGCA CTGGTACCTT TGAGGATGGG AAGAACCCTG GTCTTGTAGT AGCAGATCTC TGTGAAAAAG   400
Mmaj28Si4long-F1              ---------- ---------- ---------- ---------- ---------- ---------- ---------- ---------- ---------- ----------   400
Metarhizium_sp_EAMa_00/09-Su  ATGTTTGACC ACGAACGGTA CAGGCGCCCG TGCGTCAGCA CTGGTACCTT TGAGGATGGG AAGAACCCTG GTCTTGTAGT AGCAGATCTC TGTGAAAAAG   400
Metarhizium_sp_EAMa_01/16-Su  ATGTTTGACC ACGAACGGTA CAGGCGCCCG TGCGTCAGCA CTGGTACCTT TGAGGATGGG AAGAACCCTG GTCTTGTAGT AGCAGATCTC TGTGAAAAAG   400
Metarhizium_sp_EAMa_01/94-Su  ATGTTTGACC ACGAACGGTA CAGGCGCCCG TGCGTCAGCA CTGGTACCTT TGAGGATGGG AAGAACCCTG GTCTTGTAGT AGCAGATCTC TGTGAAAAAG   400
```

```
Meta28Si4long-F1              ---------- ---------- ---------- ---------- ---------- ---------- ---------- ---------- ---------- ----------   500
Mcar28Si4long-F1              ---------- ---------- ---------- ---------- ---------- ---------- ---------- ---------- ---------- ----------   500
M_caribense_INISAV_LBM-30     CCGGGCGATT CCTAGCCAGT ATCGGGAAGG GTGACCCAGA AGGGCATAGA GGCAGAGATT TTGACTTTGA GCCTAGCTCC TCAGTAGCAC CATCGTCAGT   500
M_caribense_INISAV_LBM-41     CCGGGCGATT CCTAGCCAGT ATCGGGAAGG GTGACCCAGA AGGGCATAGA GGCAGAGATT TTGACTTTGA GCCTAGCTCC TCAGTAGCAC CATCGTCAGT   500
M_caribense_INISAV_LBM-42     CCGGGCGATT CCTAGCCAGT ATCGGGAAGG GTGACCCAGA AGGGCATAGA GGCAGAGATT TTGACTTTGA GCCTAGCTCC TCAGTAGCAC CATCGTCAGT   500
M_caribense_ARSEF_5197        CCGGGCGATT CCTAGCCAGT ATCGGGAAGG GTGACCCAGA AGGGCATAGA GGCAGAGATT TTGACTTTGA GCCTAGCTCC TCAGTAGCAC CATCGTCAGT   500
Mmaj28Si4long-F1              ---------- ---------- ---------- ---------- ---------- ---------- ---------- ---------- ---------- ----------   500
Metarhizium_sp_EAMa_00/09-Su  CCGGGCGATT CCTAGCCAGT ATCGGGAAGG GTGACCCAGA AGGGCATAGA GGCAGAGACT TTGACTTTGA GCCTAGCTCC TCGGTAGCAC CATCGTCAGT   500
Metarhizium_sp_EAMa_01/16-Su  CCGGGCGATT CCTAGCCAGT ATCGGGAAGG GTGACCCAGA AGGGCATAGA GGCAGAGACT TTGACTTTGA GCCTAGCTCC TCGGTAGCAC CATCGTCAGT   500
Metarhizium_sp_EAMa_01/94-Su  CCGGGCGATT CCTAGCCAGT ATCGGGAAGG GTGACCCAGA AGGGCATAGA GGCAGAGACT TTGACTTTGA GCCTAGCTCC TCGGTAGCAC CATCGTCAGT   500

Meta28Si4long-F1              ---------- ---------- ---------- ---------- ---------- ---------- ---------- ---------- ---------- ----------   600
Mcar28Si4long-F1              ---------- ---------- ---------- ---------- ---------- ---------- ---------- ---------- ---------- ----------   600
M_caribense_INISAV_LBM-30     ACTCGGTGAT AATGGCTGTG GGAAGGAAAG CCAAGCTTCG GGGGACCTCG AGGTTTGTGA AATTCCGGAT AGTCAGGATT CACAAGGGGC CCGGCTGAGA   600
M_caribense_INISAV_LBM-41     ACTCGGTGAT AATGGCTGTG GGAAGGAAAG CCAAGCTTCG GGGGACCTCG AGGTTTGTGA AATTCCGGAT AGTCAGGATT CACAAGGGGC CCGGCTGAGA   600
M_caribense_INISAV_LBM-42     ACTCGGTGAT AATGGCTGTG GGAAGGAAAG CCAAGCTTCG GGGGACCTCG AGGTTTGTGA AATTCCGGAT AGTCAGGATT CACAAGGGGC CCGGCTGAGA   600
M_caribense_ARSEF_5197        ACTCGGTGAT AATGGCTGTG GGAAGGAAAG CCAAGCTTCG GGGGACCTCG AGGTTTGTGA AATTCCGGAT AGTCAGGATT CACAAGGGGC CCGGCTGAGA   600
Mmaj28Si4long-F1              ---------- ---------- ---------- ---------- ---------- ---------- ---------- ---------- ---------- ----------   600
Metarhizium_sp_EAMa_00/09-Su  ACTCGGTGAT AATGGCTGTG CGAAGGAAAG CCAAGCTTCG GGGGACCTCG AGGTTTGTGA AATTCCGGAT AGTCAGGAGT CACAAGGGGC CCGGCTGAGA   600
Metarhizium_sp_EAMa_01/16-Su  ACTCGGTGGT AATGGCTGTG CGAAGGAAAG CCAAGCTTCG GGGGACCTCG AGGTTTGTGA AATTCCGGAT AGTCAGGAGT CACAAGGGGC CCGGCTGAGA   600
Metarhizium_sp_EAMa_01/94-Su  ACTCGGTGAT AATGGCTGTG CGAAGGAAAG CCAAGCTTCG GGGGACCTCG AGGTTTGTGA AATTCCGGAT AGTCAGGAGT CACAAGGGGC CCGGCTGAAA   600

Meta28Si4long-F1              ---------- ---------- ---------- ---------- ---------- ---------- ---------- ---------- ---------- ----------   700
Mcar28Si4long-F1              ---------- ---------- ---------- ---------- ---------- ---------- ---------- ---------- ---------- ----------   700
M_caribense_INISAV_LBM-30     AGTTGGGTCC CGGGTTTTGG CGGCG---AT GAGGAGATCC GTGAAAGTGA TTTCATGGAA CTCGCGGAGG AGGAGGAAGC GATTGAACTC CCTGAGCAAT   700
M_caribense_INISAV_LBM-41     AGTTGGGTCC CGGGTTTTGG CGGCG---AT GAGGAGATCC GTGAAAGTGA TTTCATGGAA CTCGCGGAGG AGGAGGAAGC GATTGAACTC CCTGAGCAAT   700
M_caribense_INISAV_LBM-42     AGTTGGGTCC CGGGTTTTGG CGGCG---AT GAGGAGATCC GTGAAAGTGA TTTCATGGAA CTCGCGGAGG AGGAGGAAGC GATTGAACTC CCTGAGCAAT   700
M_caribense_ARSEF_5197        AGTTGGGTCC CGGGTTTTGG CGGCG---AT GAGGAGATCC GTGAAAGTGA TTTCATGGAA CTCGCGGAGG AGGAGGAAGC GATTGAACTC CCTGAGCAAT   700
Mmaj28Si4long-F1              ---------- ---------- ---------- ---------- ---------- ---------- ---------- ---------- ---------- ----------   700
Metarhizium_sp_EAMa_00/09-Su  AGTCGGGTCC CGGGTTTTGA CGGCGGTGAT GAGGAGATCC GTGAAAGTGA TTTCATGGAA CTCGCGGAGG AGGAGGAAGC GATTGAACTC CCTGAGCAAT   700
Metarhizium_sp_EAMa_01/16-Su  AGTCGGGTCC CGGGTTTTGA CGGCGGTGAT GAGGAGATCC GTGAAAGTGA TTTCATGGAA CTCGCGGAGG AGGAGGAAGC GATTGAACTC CCTGAGCAAT   700
Metarhizium_sp_EAMa_01/94-Su  AGTCGGGTCC CGGGTTTTGA CGGCGGTGAT GAGGAAATCC CTGAAAGTGA TTTCATGGAA TTCTCGGAGG AGGAGGAAGC GATTGAACTC CCTGAGCAAT   700

Meta28Si4long-F1              ---------- ---------- ---------- ---------- ---------- ---------- ---------- ---------- ---------- ----------   800
Mcar28Si4long-F1              ---------- ---------- ---------- ---------- ---------- ---------- ---------- ---------- ---------- ----------   800
M_caribense_INISAV_LBM-30     CCGGACCACC AGTAATGGCG GGCTGGGGCG TCCAGAGCAG CGCAATTTCT TCTGGGGAGA TGCCTTCGGG TGACTCTCCT CTCCAAGGCG ACAGGCGGTC   800
M_caribense_INISAV_LBM-41     CCGGACCACC AGTAATGGCG GGCTGGGGCG TCCAGAGCAG CGCAATTTCT TCTGGGGAGA TGCCTTCGGG TGACTCTCCT CTCCAAGGCG ACAGGCGGTC   800
M_caribense_INISAV_LBM-42     CCGGACCACC AGTAATGGCG GGCTGGGGCG TCCAGAGCAG CGCAATTTCT TCTGGGGAGA TGCCTTCGGG TGACTCTCCT CTCCAAGGCG ACAGGCGGTC   800
M_caribense_ARSEF_5197        CCGGACCACC AGTAATGGCG GGCTGGGGCG TCCAGAGCAG CGCAATTTCT TCTGGGGAGA TGCCTTCGGG TGACTCTCCT CTCCAAGGCG ACAGGCGGTC   800
Mmaj28Si4long-F1              ---------- ---------- ---------- ---------- ---------- ---------- ---------- ---------- ---------- ----------   800
Metarhizium_sp_EAMa_00/09-Su  CCGGACCACC AGTAATGGCG GGCTGGGGCG TCCAGAGCAG CGCAATTTCT TCTGGGGAGA TACCTTCGGG TGACTCTCCT CTCCAAAGCG ACGGGCGGTC   800
Metarhizium_sp_EAMa_01/16-Su  CCGGACCACC AGTAATGGCG GGCTGGGGCG TCCAGAGCAG CGCAATTTCT TCTGGGGAGA TACCTTCGGG TGACTCTCCT CTCCAAAGCG ACGGGCGGTC   800
Metarhizium_sp_EAMa_01/94-Su  CCGGACCACC AATAATGGCG GGCTGGGGCG TCCAGAGCAG CGCAATTTCT TCTGGGGAGA TACCTTCGGG TGACTCTCCT CTCCAAAGCG ACGGGCGGTC   800
```

Meta28Si4long-F1	----------	----------	----------	----------	----------	----------	----------	----------	----------	----------	900
Mcar28Si4long-F1	----------	----------	----------	----------	----------	----------	----------	----------	----------	----------	900
*M_caribense*_INISAV_LBM-30	GTCTACTAAG	ATGAGAGCAG	CAACATTAGG	TTATCTCTGG	GGGTTCGCTC	GTCGGCCCAG	CGAGTTACGG	GAGCAATTCC	GTTTACTTGT	AACCAGGTCT	900
*M_caribense*_INISAV_LBM-41	GTCTACTAAG	ATGAGAGCAG	CAACATTAGG	TTATCTCTGG	GGGTTCGCTC	GTCGGCCCAG	CGAGTTACGG	GAGCAATTCC	GTTTACTTGT	AACCAGGTCT	900
*M_caribense*_INISAV_LBM-42	GTCTACTAAG	ATGAGAGCAG	CAACATTAGG	TTATCTCTGG	GGGTTCGCTC	GTCGGCCCAG	CGAGTTACGG	GAGCAATTCC	GTTTACTTGT	AACCAGGTCT	900
*M_caribense*_ARSEF_5197	GTCTACTAAG	ATGAGAGCAG	CAACATTAGG	TTATCTCTGG	GGGTTCGCTC	GTCGGCCCAG	CGAGTTACGG	GAGCAATTCC	GTTTACTTGT	AACCAGGTCT	900
Mmaj28Si4long-F1	----------	----------	----------	----------	----------	----------	----------	----------	----------	----------	900
*Metarhizium*_sp_EAMa_00/09-Su	GTCTACTAAG	ATGAGAGCAG	CAACATTAGG	TTATCTCTGG	GGGTTCGCTC	GTCGGCCCAG	CGAGTTACGG	GAGCAATTCC	GTTTACTTGT	AACCAGGTCT	900
*Metarhizium*_sp_EAMa_01/16-Su	GTCTACTAAG	ATGAGAGCAG	CAACATTAGG	TTATCTCTGG	GGGTTCGCTC	GTCGGCCCAG	CGAGTTACGG	GAGCAATTCC	GTTTACTTGT	AACCAGGTCT	900
*Metarhizium*_sp_EAMa_01/94-Su	GTCTACTAAG	ATGAGAGCAG	CAACATTAGG	TTATCTCTGG	GGGTTCGCTC	GTCGGCCCAG	CGAGTTACGG	GAGCAATTCC	GTTTACTTGT	AACCAGGTCT	900

Meta28Si4long-F1	----------	----------	----------	----------	----------	----------	----------	----------	----------	----------	1000
Mcar28Si4long-F1	----------	----------	----------	----------	----------	----------	----------	----------	----------	----------	1000
*M_caribense*_INISAV_LBM-30	GACTTACATG	TGCTGCACCT	CTGTGGTTGT	GGGATGTGCT	ATACTACAGG	TAGTGGGGTT	CGGATTCCTG	GATGTGTAGA	ACATTCTCAC	CTCCGCTTAG	1000
*M_caribense*_INISAV_LBM-41	GACTTACATG	TGCTGCACCT	CTGTGGTTGT	GGGATGTGCT	ATACTACAGG	TAGTGGGGTT	CGGATTCCTG	GATGTGTAGA	ACATTCTCAC	CTCCGCTTAG	1000
*M_caribense*_INISAV_LBM-42	GACTTACATG	TGCTGCACCT	CTGTGGTTGT	GGGATGTGCT	ATACTACAGG	TAGTGGGGTT	CGGATTCCTG	GATGTGTAGA	ACATTCTCAC	CTCCGCTTAG	1000
*M_caribense*_ARSEF_5197	GACTTACATG	TGCTGCACCT	CTGTGGTTGT	GGGATGTGCT	ATACTACAGG	TAGTGGGGTT	CGGATTCCTG	GATGTGTAGA	ACATTCTCAC	CTCCGCTTAG	1000
Mmaj28Si4long-F1	----------	----------	----------	----------	----------	----------	----------	----------	----------	----------	1000
*Metarhizium*_sp_EAMa_00/09-Su	GACTTACATG	TGCTGCACCT	CTGTGGTTGT	GGGATGTGCT	ATACTACAGG	TAGTGGGGTT	CGGATTCCTG	GATGTGTAGA	ACATTCTCAC	CTCCGCTTAG	1000
*Metarhizium*_sp_EAMa_01/16-Su	GACTTACATG	TGCTGCACCT	CTGTGGTTGT	GGGATGTGCT	ATACTACAGG	TAGTGGGGTT	CGGATTCCTG	GATGTGTAGA	ACATTCTCAC	CTCCGCTTAG	1000
*Metarhizium*_sp_EAMa_01/94-Su	GACTTACATG	TGCTGCACCT	CTGTGGTTGT	GGGATGTGCT	ATACTACAGG	TAGTGGGGTT	CGGATTCCTG	GATGTGTAGA	ACATTCTCAC	CTCCGCTTAG	1000

Meta28Si4long-F1	----------	----------	----------	----------	----------	----------	----------	----------	----------	----------	1100
Mcar28Si4long-F1	----------	----------	----------	----------	----------	----------	----------	----------	----------	----------	1100
*M_caribense*_INISAV_LBM-30	GCTCGTCGAG	GGAAAACGGC	CACCACCGGA	ATTTCCATAT	TGTGCTCAGC	TTCACACCGG	TAGAGAATTA	TCCCGTTCAG	TGCGGTTTAC	TTCACCAGTC	1100
*M_caribense*_INISAV_LBM-41	GCTCGTCGAG	GGAAAACGGC	CACCACCGGA	ATTTCCATAT	TGTGCTCAGC	TTCACACCGG	TAGAGAATTA	TCCCGTTCAG	TGCGGTTTAC	TTCACCAGTC	1100
*M_caribense*_INISAV_LBM-42	GCTCGTCGAG	GGAAAACGGC	CACCACCGGA	ATTTCCATAT	TGTGCTCAGC	TTCACACCGG	TAGAGAATTA	TCCCGTTCAG	TGCGGTTTAC	TTCACCAGTC	1100
*M_caribense*_ARSEF_5197	GCTCGTCGAG	GGAAAACGGC	CACCACCGGA	ATTTCCATAT	TGTGCTCAGC	TTCACACCGG	TAGAGAATTA	TCCCGTTCAG	TGCGGTTTAC	TTCACCAGTC	1100
Mmaj28Si4long-F1	----------	----------	----------	----------	----------	----------	----------	----------	----------	----------	1100
*Metarhizium*_sp_EAMa_00/09-Su	GCTCGTCGAG	GGAAAACGGC	CACCACCGGA	ATTTCCACAT	TGTGCTCAGC	TTCACACCGG	TAGAGAATTA	TCCCGTTCAG	TGCGGTTTAC	TTCACCAGTC	1100
*Metarhizium*_sp_EAMa_01/16-Su	GCTCGTCGAG	GGAAAACGGC	CACCACCGGA	ATTTCCACAT	TGTGCTCAGC	TTCACACCGG	TAGAGAATTA	TCCCGTTCAG	TGCGGTTTAC	TTCACCAGTC	1100
*Metarhizium*_sp_EAMa_01/94-Su	GCTCGTCGAG	GGAAAACGGC	CACCACCGGA	ATTTCCACAT	TGTGCTCAGC	TTCACACCGG	TAGAGAATTA	TCCCGTTCAG	TGCGGTTTAC	TTCACCAGTC	1100

Meta28Si4long-F1	----------	----------	----------	----------	----------	----------	----------	----------	----------	----------	1200
Mcar28Si4long-F1	----------	----------	----------	----------	----------	----------	----------	----------	----------	----------	1200
*M_caribense*_INISAV_LBM-30	TGACAGCGAG	TATGGTATCG	GAGTATTCTG	ATGCGGCGAG	TTGGTTTATA	CGAGCAGATA	CGTTCTTCAT	AATACTTGCT	TCAACCTCGC	GGCAGGGGTC	1200
*M_caribense*_INISAV_LBM-41	TGACAGCGAG	TATGGTATCG	GAGTATTCTG	ATGCGGCGAG	TTGGTTTATA	CGAGCAGATA	CGTTCTTCAT	AATACTTGCT	TCAACCTCGC	GGCAGGGGTC	1200
*M_caribense*_INISAV_LBM-42	TGACAGCGAG	TATGGTATCG	GAGTATTCTG	ATGCGGCGAG	TTGGTTTATA	CGAGCAGATA	CGTTCTTCAT	AATACTTGCT	TCAACCTCGC	GGCAGGGGTC	1200
*M_caribense*_ARSEF_5197	TGACAGCGAG	TATGGTATCG	GAGTATTCTG	ATGCGGCGAG	TTGGTTTATA	CGAGCAGATA	CGTTCTTCAT	AATACTTGCT	TCAACCTCGC	GGCAGGGGTC	1200
Mmaj28Si4long-F1	----------	----------	----------	----------	----------	----------	----------	----------	----------	----------	1200
*Metarhizium*_sp_EAMa_00/09-Su	TGACAGTGGG	TATGGTGTCG	GAGTATTCTG	ATGCGGCGAG	TGGTTTTATA	CGAGCAGATA	CGTTCTTCAT	AATACTTGCT	TCAACCTCGC	GGCAGGTGTC	1200
*Metarhizium*_sp_EAMa_01/16-Su	TGACAGTGGG	TATGGTGTCG	GAGTATTCTG	ATGCGGCGAG	TGGTTTTATA	CGAGCAGATA	CGTTCTTCAT	AATACTTGCT	TCAACCTCGC	GGCAGGTGTC	1200
*Metarhizium*_sp_EAMa_01/94-Su	TGACAGTGGG	TATGGTGTCG	GAGTATTCTG	ATGCGGCGAG	TGGTTTTATA	CGAGCAGATA	CGTTCTTCAT	AATACTTGCT	TCAACCTCGC	GGCAGGTGTC	1200

```
Meta28Si4long-F1              ---------- -TAGGTGAAG GGGCTGCAAG TC-------- ---------- ---------- ---------- ---------- ---------- ----------  1300
Mcar28Si4long-F1              ---------- ---------- ---------- ---------- ---------- -AAGGCGT-- ATCGCAGTAT GACT------ ---------- ----------  1300
M_caribense_INISAV_LBM-30     CGGATCCGCT GTAGGTGAAG GGGCTGCAAG TCTTGGGCGG GTAGGTATTG TAAGGCGT-- ATCGCAGTAT GACTAACAGT TAATTTCTCT AGAGGGGTTT  1300
M_caribense_INISAV_LBM-41     CGGATCCGCT GTAGGTGAAG GGGCTGCAAG TCTTGGGCGG GTAGGTATTG TAAGGCGT-- ATCGCAGTAT GACTAACAGT TAATTTCTCT AGAGGGGTTT  1300
M_caribense_INISAV_LBM-42     CGGATCCGCT GTAGGTGAAG GGGCTGCAAG TCTTGGGCGG GTAGGTATTG TAAGGCGT-- ATCGCAGTAT GACTAACAGT TAATTTCTCT AGAGGGGTTT  1300
M_caribense_ARSEF_5197        CGGATCCGCT GTAGGTGAAG GGGCTGCAAG TCTTGGGCGG GTAGGTATTG TAAGGCGT-- ATCGCAGTAT GACTAACAGT TAATTTCTCT AGAGGGGTTT  1300
Mmaj28Si4long-F1              ---------- ---------- ---------- ---------- ---------- ---GGCGTGT ATTGCAGTAT AACC------ ---------- ----------  1300
Metarhizium_sp_EAMa_00/09-Su  TGGATCCGCT GTAGGTGAAG GGGCTGCAAG TCTTGGGCGG GTAGGTATTG TAAGGCGTGT ATTGCAGTAT AACCA-CAGT TAATTTCTCT AGAGGGGTTT  1300
Metarhizium_sp_EAMa_01/16-Su  TGGATCCGCT GTAGGTGAAG GGGCTGCAAG TCTTGGGCGG GTAGGTATTG TAAGGCGTGT ATTGCAGTAT AACCA-CAGT TAATTTCTCT AGAGGGGTTT  1300
Metarhizium_sp_EAMa_01/94-Su  TGGATCCGCT GTAGGTGAAG GGGCTGCAAG TCTTGGGCGG GTAGGTATTG TAAGGCGTGT ATTGCAGTAT AACCA-CAGT TAATTTCTCT AGAGGGGTTT  1300

Meta28Si4long-F1              ---------- ---------- ---------- ---------- ---------- ---------- ---------- ---------- ---------- ----------  1400
Mcar28Si4long-F1              ---------- ---------- ---------- ---------- ---------- ---------- ---------- ---------- ---------- ----------  1400
M_caribense_INISAV_LBM-30     CGCGATGCGT GTTGTATATA ATTTGTTTCT TGTTGGTGGG AATAGTCAGG TCTCAGGGAG GGGCTGAGAT TATGCTGGCT AGTCGAGATG GCATATCGCT  1400
M_caribense_INISAV_LBM-41     CGCGATGCGT GTTGTATATA ATTTGTTTCT TGTTGGTGGG AATAGTCAGG TCTCAGGGAG GGGCTGAGAT TATGCTGGCT AGTCGAGATG GCATATCGCT  1400
M_caribense_INISAV_LBM-42     CGCGATGCGT GTTGTATATA ATTTGTTTCT TGTTGGTGGG AATAGTCAGG TCTCAGGGAG GGGCTGAGAT TATGCTGGCT AGTCGAGATG GCATATCGCT  1400
M_caribense_ARSEF_5197        CGCGATGCGT GTTGTATATA ATTTGTTTCT TGTTGGTGGG AATAGTCAGG TCTCAGGGAG GGGCTGAGAT TATGCTGGCT AGTCGAGATG GCATATCGCT  1400
Mmaj28Si4long-F1              ---------- ---------- ---------- ---------- ---------- ---------- ---------- ---------- ---------- ----------  1400
Metarhizium_sp_EAMa_00/09-Su  CGCGATGCGT GTTGTATATA ATTTGTTTCT TGTTGGTGGG AATAGTCAGG TCTCAGGGAG GGGCTGAGAT TATGCTGGCT AGTCGAGATG GCATATCGCT  1400
Metarhizium_sp_EAMa_01/16-Su  CGCGATGCGT GTTGTATATA ATTTGTTTCT TGTTGGTGGG AATAGTCAGG TCTCAGGGAG GGGCTGAGAT TATGCTGGCT AGTCGAGATG GCATATCGCT  1400
Metarhizium_sp_EAMa_01/94-Su  CGCGATGCGT GTTGTATATA ATTTGTTTCT TGTTGGTGGG AATAGTCAGG TCTCAGGGAG GGGCTGAGAT TATGCTGGCT AGTCGAGATG GCATATCGCT  1400

Meta28Si4long-F1              ---------- ---------- ---------- ---------- ---------- ---------- ---------- ---------- ---------- ----------  1500
Mcar28Si4long-F1              ---------- ---------- ---------- ---------- ---------- ---------- ---------- ---------- ---------- ----------  1500
M_caribense_INISAV_LBM-30     GCAGGTGCCA TCCGGCGACA CAACCTGGAT CGGGGGAGGC TAAGGGCCGA AAGGTCTATG CTAATCCCGA GGGGAGCCCT ATGAGAGTGA TCTTGTAATG  1500
M_caribense_INISAV_LBM-41     GCAGGTGCCA TCCGGCGACA CAACCTGGAT CGGGGGAGGC TAAGGGCCGA AAGGTCTATG CTAATCCCGA GGGGAGCCCT ATGAGAGTGA TCTTGTAATG  1500
M_caribense_INISAV_LBM-42     GCAGGTGCCA TCCGGCGACA CAACCTGGAT CGGGGGAGGC TAAGGGCCGA AAGGTCTATG CTAATCCCGA GGGGAGCCCT ATGAGAGTGA TCTTGTAATG  1500
M_caribense_ARSEF_5197        GCAGGTGCCA TCCGGCGACA CAACCTGGAT CGGGGGAGGC TAAGGGCCGA AAGGTCTATG CTAATCCCGA GGGGAGCCCT ATGAGAGTGA TCTTGTAATG  1500
Mmaj28Si4long-F1              ---------- ---------- ---------- ---------- ---------- ---------- ---------- ---------- ---------- ----------  1500
Metarhizium_sp_EAMa_00/09-Su  GCAGGTGCCA TCCGGCGACA CAACCTGGAT CGGGGGAGGC TAAGGGCCGA GAGGTCTATG CTAATCCCGA GGGGAGCCCT ATGAGAGTGA TCTTGTAATG  1500
Metarhizium_sp_EAMa_01/16-Su  GCAGGTGCCA TCCGGCGACA CAACCTGGAT CGGGGGAGGC TAAGGGCCGA GAGGTCTATG CTAATCCCGA GGGGAGCCCT ATGAGAGTGA TCTTGTAATG  1500
Metarhizium_sp_EAMa_01/94-Su  GCAGGTGCCA TCCGGCGACA CAACCTGGAT CGGGGGAGGC TAAGGGCCGA GAGGTCTATG CTAATCCCGA GGGGAGCCCT ATGAGAGTGA TCTTGTAATG  1500

Meta28Si4long-F1              ---------- ---------- ---------- ---------- ---------- ---------- ---------- ---------- ---------- ----------  1600
Mcar28Si4long-F1              ---------- ---------- ---------- ---------- ---------- ---------- ---------- ---------- ---------- ----------  1600
M_caribense_INISAV_LBM-30     GCCCCTGTAG AGCGCGGAAA GGTGTGGGCA ACTCTCGAAT ATTCGAGGTT GCTTAAGGGA CGTGCCAGGC CCACGGGAAA CCGTGCCGTG TGCGAAGGAC  1600
M_caribense_INISAV_LBM-41     GCCCCTGTAG AGCGCGGAAA GGTGTGGGCA ACTCTCGAAT ATTCGAGGTT GCTTAAGGGA CGTGCCAGGC CCACGGGAAA CCGTGCCGTG TGCGAAGGAC  1600
M_caribense_INISAV_LBM-42     GCCCCTGTAG AGCGCGGAAA GGTGTGGGCA ACTCTCGAAT ATTCGAGGTT GCTTAAGGGA CGTGCCAGGC CCACGGGAAA CCGTGCCGTG TGCGAAGGAC  1600
M_caribense_ARSEF_5197        GCCCCTGTAG AGCGCGGAAA GGTGTGGGCA ACTCTCGAAT ATTCGAGGTT GCTTAAGGGA CGTGCCAGGC CCACGGGAAA CCGTGCCGTG TGCGAAGGAC  1600
Mmaj28Si4long-F1              ---------- ---------- ---------- ---------- ---------- ---------- ---------- ---------- ---------- ----------  1600
Metarhizium_sp_EAMa_00/09-Su  GCCCCTGTAG AGCGCGGAAA GGTGTGGGCA ACTCTCGAAT ATTCGAGGTT GCTTAAGGGA CGTGCCAGGC CCACGGGAAA CCGTGCCGTG TGCGAAGGAC  1600
Metarhizium_sp_EAMa_01/16-Su  GCCCCTGTAG AGCGCGGAAA GGTGTGGGCA ACTCTCGAAT ATTCGAGGTT GCTTAAGGGA CGTGCCAGGC CCACGGGAAA CCGTGCCGTG TGCGAAGGAC  1600
Metarhizium_sp_EAMa_01/94-Su  GCCCCTGTAG AGCGCGGAAA GGTGTGGGCA ACTCTCGAAT ATTCGAGGTT GCTTAAGGGA CGTGCCAGGC CCACGGGAAA CCGTGCCGTG TGCGAAGGAC  1600
```

```
Meta28Si4long-F1              ---------- ---------- ---------- ---------- ---------- ---------- ---------- ---------- ---------- ----------  1700
Mcar28Si4long-F1              ---------- ---------- ---------- ---------- ---------- ---------- ---------- ---------- ---------- ----------  1700
M_caribense_INISAV_LBM-30     CTGCAGTCTG GATCACATGG AAAAGCGTTT GAGTAGAGGG GGGAAATACC CTTTGCGAGC TTAGGTACTG ATCACATATG TAGGGGATGA AGTATCAACT  1700
M_caribense_INISAV_LBM-41     CTGCAGTCTG GATCACATGG AAAAGCGTTT GAGTAGAGGG GGGAAATACC CTTTGCGAGC TTAGGTACTG ATCACATATG TAGGGGATGA AGTATCAACT  1700
M_caribense_INISAV_LBM-42     CTGCAGTCTG GATCACATGG AAAAGCGTTT GAGTAGAGGG GGGAAATACC CTTTGCGAGC TTAGGTACTG ATCACATATG TAGGGGATGA AGTATCAACT  1700
M_caribense_ARSEF_5197        CTGCAGTCTG GATCACATGG AAAAGCGTTT GAGTAGAGGG GGGAAATACC CTTTGCGAGC TTAGGTACTG ATCACATATG TAGGGGATGA AGTATCAACT  1700
Mmaj28Si4long-F1              ---------- ---------- ---------- ---------- ---------- ---------- ---------- ---------- ---------- ----------  1700
Metarhizium_sp_EAMa_00/09-Su  CTGCAGTCTG GATCACATGG AAGAGCGTTT GAGCAGAGGG GGGAAATACC CTCTGCGAGC TTAGGTACTG ATCACATATG TAGGGGATGA AGTATCAACT  1700
Metarhizium_sp_EAMa_01/16-Su  CTGCAGTCTG GATCACATGG AAGAGCGTTT GAGCAGAGGG GGGAAATACC CTCTGCGAGC TTAGGTACTG ATCACATATG TAGGGGATGA AGTATCAACT  1700
Metarhizium_sp_EAMa_01/94-Su  CTGCAGTCTG GATCACATGG AAGAGCGTTT GAGCAGAGGG GGGAAATACC CTCTGCGAGC TTAGGTACTG ATCACATATG TAGGGGATGA AGTATCAACT  1700

Meta28Si4long-F1              ---------- ---------- ---------- ---------- ---------- ----- 1755
Mcar28Si4long-F1              ---------- ---------- ---------- ---------- ---------- ----- 1755
M_caribense_INISAV_LBM-30     GCCTAGCCGG AGTTGGGCTA TGATGGCACT GATAATTGGG TGTGTGATCG CCCCG 1755
M_caribense_INISAV_LBM-41     GCCTAGCCGG AGTTGGGCTA TGATGGCACT GATAATTGGG TGTGTGATCG CCCCG 1755
M_caribense_INISAV_LBM-42     GCCTAGCCGG AGTTGGGCTA TGATGGCACT GATAATTGGG TGTGTGATCG CCCCG 1755
M_caribense_ARSEF_5197        GCCTAGCCGG AGTTGGGCTA TGATGGCACT GATAATTGGG TGTGTGATCG CCCCG 1755
Mmaj28Si4long-F1              ---------- ---------- ---------- ---------- ---------- ----- 1755
Metarhizium_sp_EAMa_00/09-Su  GCCTAGCCGG AGTTGGGCTA TGAGGGCACT GATAATTGGG TGTGTGATCG CCCCG 1755
Metarhizium_sp_EAMa_01/16-Su  GCCTAGCCGG AGTTGGGCTA TGAGGGCACT GATAATTGGG TGTGTGATCG CCCCG 1755
Metarhizium_sp_EAMa_01/94-Su  GC-------- ---------- ---------- ---------- ---------- ----- 1755
```
