# Supplementary material for: Metarhizium caribense sp. nov., a Novel Species of Entomopathogenic Metarhizium Fungi Associated with Weevils Impairing Coffee, Sugar Cane and Sweet Potato Cultivation
Source: J Fungi (Basel). 2024 Aug 28;10(9):612. doi: 10.3390/jof10090612 (PMC11433170; doi:10.3390/jof10090612)
Supplement: Supplementary file 1 [file jof-10-00612-s001.zip › Suppl Table S1.pdf]

Supplementary Table S1. Fungal strains and GenBank accession numbers of marker sequences referred to in this study. "-T" following a strain designation denotes a nomenclatural type strain.

Genus, Species	Strain	rIGS complete	rIGS-ID800	DUF895 (=MzIGS3)	5TEF	EF1A	RPB1	RPB2	28Si4	Geographic Origin	Original Host / Isolation Substrate	References
Metarhizium acridum	ARSEF 7486 - T				EU248845	EU248845	EU248897	EU248925		Niger	Orthoptera	[6]
Metarhizium anisopliae	ARSEF 798	OR296677		KX342447	KX342696	OR296642	OR296653	OR296664		Colombia	*Aeneolamia varia*	[14], [19]
Metarhizium anisopliae	ARSEF 1080	OR296678		KX342449	KX342698	OR296643	OR296654	OR296665		USA	*Helicoverpa zea*	[14], [19]
Metarhizium anisopliae	ARSEF 6347	MH604976		PP034599	EU248881	OR296644	OR296655	OR296666		Colombia	*Hemiptera*	[6], [19], [22], this study
Metarhizium anisopliae	ARSEF 7450	MH604977		PP034600	EU248852	EU248852	EU248904	EU248932		Australia	Coleoptera	[6], [22], this study
Metarhizium anisopliae	ARSEF 7487 - T	MH604974		KC164558	DQ463996	DQ463996	DQ468355	DQ468370		Ethiopia	Orthoptera	[9], [18], [22]
Metarhizium anisopliae	INISAV LBM-5		OR441058	PP034589	MZ329397	MZ292434	MZ292440	MZ292446		Cuba	undetermined Hemipteran insect	[19], [31], this study
Metarhizium anisopliae	INISAV LBM-10		OR441060	PP034591	MZ329398	MZ292435	MZ292441	MZ292447		Cuba	undetermined Hemipteran insect	[19], [31], this study
Metarhizium anisopliae	INISAV LBM-11		OR441061	PP034592	MZ329399	MZ292436	MZ292442	MZ292448		Cuba	*Mocis latipes* (Lepidoptera: Erebidae)	[19], [31], this study
Metarhizium anisopliae	INISAV LBM-12		OR441062	PP034593	MZ329400	MZ292437	MZ292443	MZ292449		Cuba	*Corcyra cephalonica* (Lepidoptera: Pyralidae)	[19], [31], this study
Metarhizium anisopliae	INISAV LBM-267		OR441064	PP034598	MZ329402	MZ292439	MZ292445	MZ292451		Cuba	Leaf hopper (Hemiptera: Cicadellidae)	[19], [31], this study
Metarhizium brunneum	ARSEF 1066	OR296679		KX342514	KX342763	OR296645	OR296656	OR296667		Switzerland	*Melolontha melolontha*	[14], [19]
Metarhizium brunneum	ARSEF 1278	OR296680		KX342523	KX342772	OR296646	OR296657	OR296668		Canada	soil	[14], [19]
Metarhizium brunneum	ARSEF 2107 - T	MH604983		KC164556	EU248855	EU248855	EU248907	EU248935		USA	Coleoptera	[6], [18], [22]
Metarhizium brunneum	ARSEF 3295	OR296681		KX342510	KX342759	OR296647	OR296658	OR296669		Mexico	*Anticarsia gemmatalis*	[14], [19]
Metarhizium brunneum	ARSEF 3826	MH604984		KX342520	KX342769	OR296648	OR296659	OR296670		USA	*Ochlerotatus triseriatus*	[14], [19], [22]

Metarhizium brunneum	ARSEF 4152	MH604985		KX342508	KX342757	EU248853	EU248905	EU248933		Australia	soil	[6], [14], [22]
Metarhizium brunneum	ARSEF 4179	MH604986			EU248854	EU248854	EU248906	EU248934		Australia	soil	[6], [22]
Metarhizium caribense	ARSEF 1448	PP034620		KX342464	KX342713	PP034603	PP034627	PP034641		Brazil	*Scaptores castanea*	[14], this study
Metarhizium caribense	ARSEF 5197 - T	PP034621		KX342465	KX342714	PP034604	PP034628	PP034642	OR982706	USA	*Diaprepes abbreviata*	[14], this study
Metarhizium caribense	INISAV LBM-30	PP034617		PP034594	OR982696	PP034606	PP034630	PP034644	OR982703	Cuba	Soil	this study
Metarhizium caribense	INISAV LBM-41	PP034618		PP034595	OR982697	PP034607	PP034631	PP034645	OR982704	Cuba	*Hypothenemus hampei* (Coleoptera:Curculionidae)	this study
Metarhizium caribense	INISAV LBM-42	PP034619		PP034596	OR982698	PP034608	PP034632	PP034646	OR982705	Cuba	*Hypothenemus hampei* (Coleoptera:Curculionidae)	this study
Metarhizium guizhouense	CBS 258.90 - T	MH604996		KU980515	EU248862	EU248862	EU248914	EU248942		China	Lepidoptera	[6], [22], [49]
Metarhizium humberi	ESALQ 1638	JACEFI010 000005.1		KP028040	KP027955	JACEFI010 000008.1	JACEFI010 000006.1	JACEFI010 000009.1		Brazil	Soil	[15]
Metarhizium humberi	IP 1				JQ061188	MH837571	MH837553	MH837562		Brazil	Soil	[16], [51]
Metarhizium humberi	IP 151				JQ061208	MH837579	MH837561	MH837570		Brazil	Soil	[16], [51]
Metarhizium humberi	IP 46 - T				JQ061205	MH837574	MH837556	MH837565		Brazil	Soil	[16], [51]
Metarhizium humberi	IP 86				JQ061186	MH837576	MH837558	MH837567		Brazil	Soil	[16], [51]
Metarhizium majus	ARSEF 1914 - T	MH604993		KU980508	EU248868	EU248868	EU248920	EU248948		Philippines	Coleoptera	[6], [22], [49]
Metarhizium pinghaense	ARSEF 2231	PP034622	OR400502	KX342474	KX342723	PP034609	PP034633	PP034647		India	*Zygogramma bicolorata*	[14], [19], this study
Metarhizium pinghaense	ARSEF 2809	OR296682		KX342463	KX342712	OR296649	OR296660	OR296671		Philippines	Soil	[14], [19]
Metarhizium pinghaense	ARSEF 3180	PP034623	OR400503	KX342455	KX342704	PP034610	PP034634	PP034648		Philippines	Soil	[14], [19], this study
Metarhizium pinghaense	ARSEF 3210	MH604981		LC128473	DQ463995	DQ463995	DQ468354	DQ468369		India	Coleoptera	[9], [22]
Metarhizium pinghaense	ARSEF 3604	PP034624	OR400504	KX342459	KX342708	PP034611	PP034635	PP034649		Papua New Guinea	*Scapanes australis*	[14], [19], this study

Metarhizium pinghaense	ARSEF 4290	PP034625	OR400505	KX342456	KX342705	PP034612	PP034636	PP034650		Solomon Islands	Coleoptera	[14], [19], this study
Metarhizium pinghaense	ARSEF 4342	MH604980		LC709291	EU248851	EU248851	EU248903	EU248931		Solomon Islands	Coleoptera	[6], [22]
Metarhizium pinghaense	ARSEF 7422	OR296683		KX342457	KX342706	OR296650	OR296661	OR296672		Australia	Coleoptera	[14], [19]
Metarhizium pinghaense	ARSEF 7929	MH604979		LC709290	EU248847	EU248847	EU248899	OR296675		Australia	Isoptera	[6], [19], [22]
Metarhizium pinghaense	ARSEF 9975	PP034626	OR400508	PP034602	MK156097	PP034613	PP034637	PP034651		USA	Soil	[19], [50], this study
Metarhizium pinghaense	CBS 257.90 - T	MH604978		KC164559	EU248850	EU248850	EU248902	OR296676		China	Coleoptera	[6], [18], [19], [22]
Metarhizium robertsii	ARSEF 727	MH604970		KC164557	DQ463994	DQ463994	DQ468353	DQ468368		Brazil	Orthoptera	[9], [18], [22]
Metarhizium robertsii	ARSEF 2575 - T	MH604971		KX342480	KX342729	JELW0100 0003.1	JELW0100 0006.1	JELW0100 0009.1		USA	*Curculio caryae*	[14], [22]
Metarhizium robertsii	ARSEF 7501	MH604973		KX342492	KX342741	EU248849	EU248901	EU248929		Australia	Coleoptera	[6], [14], [22]
Metarhizium robertsii	ARSEF 8070	OR296684		KX342501	KX342750	OR296651	OR296662	OR296673		Australia	mound material of *Nasutitermes exitiosus*	[14], [19]
Metarhizium robertsii	ARSEF 8685	OR296685		PP034601	FJ229494	OR296652	OR296663	OR296674		Canada	Soil	[6], [19], this study
Metarhizium robertsii	INISAV LBM-7		OR441059	PP034590	OR423400	PP034605	PP034629	PP034643		Cuba	undetermined Homopteran insect	[19], this study
Metarhizium robertsii	INISAV LBM-146		OR441063	PP034597	MZ329401	MZ292438	MZ292444	MZ292450		Cuba	Leaf hopper (Hemiptera: Cicadellidae)	[19], [31], this study
Metarhizium sp.	EAMa 00/09-Su				OR982699	PP034614	PP034638	PP034652	DQ667592	Spain	Soil	[30], this study
Metarhizium sp.	EAMa 01/16-Su				OR982700	PP034615	PP034639	PP034653	DQ667593	Spain	Soil	[30], this study
Metarhizium sp.	EAMa 01/94-Su				OR982701	PP034616	PP034640	PP034654	OR982707	Spain	Soil	this study
