# Supplementary material for: Metarhizium caribense sp. nov., a Novel Species of Entomopathogenic Metarhizium Fungi Associated with Weevils Impairing Coffee, Sugar Cane and Sweet Potato Cultivation
Source: J Fungi (Basel). 2024 Aug 28;10(9):612. doi: 10.3390/jof10090612 (PMC11433170; doi:10.3390/jof10090612)
Supplement: Supplementary file 1 [file jof-10-00612-s001.zip › Suppl Table S2.pdf]

Supplementary Table S2. Preparative PCR and sequencing primers used and reaction-specific parameters applied in this study.

Primer Designation	Primer Sequence (5′ => 3′)	Annealing Temperature (°C)	Elongation Time (sec)	References
EF1A-983F	GCYCCYGGHCAYCGTGAYTTYAT	52	90	[52]
EF1A-2218R	ATGACACCRACRGCRACRGTYTG			
EF1A-1567R	ACHGTRCCRATACCACCSATCTT	sequencing primer		
EF1A-1577F	CARGAYGTBTACAAGATYGGTGG	sequencing primer		[31]
RPB1Af	GARTGYCCDGGDCAYTTYGG	50	90	[53]
RPB1Cr	CCNGCDATNTCRTTRTCCATRTA			
RPB2-5f	GAYGAYMGWGATCAYTTYGG	50	120	[54]
RPB2-7r	CCCATRGCTTGYTTRCCCAT			
RPB2-6f	TGGGGKWTGGTYTGYCCTGC	sequencing primer		[55]
RPB2-6r	GCAGGRCARACCAWMCCCCA	sequencing primer		
EF1T	ATGGGTAAGGARGACAAGAC	64	60	[9]
EF2T	GGAAGTACCAGTGATCATGTT			
MzIGS3-1F	CGTGGCTCCTGACCATGGTTGC	55	90	[18]
MzIGS3-4R	GCGGGGGAGCCGACTTGGA			
MzIGS3-3F	TGGTRTAACCRGCGAGACA	sequencing primer		
MzIGS3-5R	CCGTCCSGCATAGCCYTCA	sequencing primer		
Migs1-F1	CCTTGTTGTTACGATCTGCTGAG	55	90	[19]
Migs850-R1	TAAASAAGCAGCCTACCCTAAAGC			
Migs1-F2	CTGAGGGTTAGCCGTTCTTCG	64	120	
Migs 1200R	CCAGCAGAGAAATCCCCTCAC			
Migs 1100F	AGAGRGAGGGCTGGACACGCG	64	120	
Migs 2000R	GGCTCAATCGATGCAGCTCGGCGAG			
28Si-I29F	CTGCCCAGTGCTCTGAATGTC	52	180	[26]
28Si-E24R	GCTGAATTACCATTGCGGAGAGG			
28Si-F1	TGGTTTTAAGGTCCTTCGCG	sequencing primer		[this study]
28Si-F2	AAGCGTTCATAGCGACGTCG	sequencing primer		
28Si-R1	AGATTTCTGTTCTCCATGAG	sequencing primer		
28Si-R2	TTTCGCTAGATAGTAGATAG	sequencing primer		
