# Supplementary material for: Metarhizium caribense sp. nov., a Novel Species of Entomopathogenic Metarhizium Fungi Associated with Weevils Impairing Coffee, Sugar Cane and Sweet Potato Cultivation
Source: J Fungi (Basel). 2024 Aug 28;10(9):612. doi: 10.3390/jof10090612 (PMC11433170; doi:10.3390/jof10090612)
Supplement: Supplementary file 1 [file jof-10-00612-s001.zip › Suppl Table S3.pdf]

Supplementary Table S3. Diagnostic PCR primers used in this study.

Primer Designation	Nucleotide Sequence (5′ => 3′)	Expected Specificity	Expected Product Size (bp)	Reference
**Species-discriminating rIGS-based diagnostic PCR**				
mani-IDF1	GGCTATAGTYAACTTTTGGACTTGC	*M. anisopliae*	335	[19]
mani-IDR1	ACAAAAAAATCAACTCACGCCTATAT			
mbru-IDF1	TGACTTKTGGACWYGGCGGA	*M. brunneum*	277–405	[19]
mbru-IDR1	CGCTACYRGGCTCTCGTGGT			
mcar-IDF1	GGACTTGGCATATTTGCTTGAATTG	*M. caribense*	345	this study
mcar-IDR1	TCTTATATACCCACCAACTACCTTG			
mpin-IDF1	GTGCCGGGGCCCTGTAG	*M. pinghaense*	337–391	[19]
mpin-IDR1	GCCAAAATACTAGGAACTTGTATA			
mrob-IDF1	GCGGGTGTTGGGGTTAAT	*M. robertsii*	841–842	[19]
mrob-IDR1	CTAAAAGTATTGGCTGCGGC			
**28Si4 intron – based diagnostic PCR**				
Meta28Si4long-F1	TAGGTGAAGGGGCTGCAAGTC	Presence of long 28Si4 intron	624	this study
Mcar28Si4long-F1	AAGGCGTATCGCAGTATGACT	*M. caribense* variety of 28Si4 intron	584	this study
Mmaj28Si4long-F1	GGCGTGTATTGCAGTATAACC	*M. majus* variety of 28Si4 intron	582	this study
28Si-E24Rshort	GAATTACCATTGCGGAGAGG	Reverse primer	n.a.	this study
