# Supplementary material for: Metarhizium caribense sp. nov., a Novel Species of Entomopathogenic Metarhizium Fungi Associated with Weevils Impairing Coffee, Sugar Cane and Sweet Potato Cultivation
Source: J Fungi (Basel). 2024 Aug 28;10(9):612. doi: 10.3390/jof10090612 (PMC11433170; doi:10.3390/jof10090612)
Supplement: Supplementary file 1 [file jof-10-00612-s001.zip › Suppl Table S4.pdf]

**Supplementary Table S4**. Pairwise sequence similarity percentages of concatenated 5TEF-MzIGS3-rIGSID800 sequences as calculated from a p-distance matrix.

| **Pairwise sequence similarity (%)** | **1** | **2** | **3** | **4** | **5** | **6** | **7** | **8** | **9** | **10** | **11** | **12** | **13** | **14** | **15** | **16** | **17** | **18** | **19** |
|---|---|---|---|---|---|---|---|---|---|---|---|---|---|---|---|---|---|---|---|
| 1 *Metarhizium_caribense* INISAV LBM-30 | | | | | | | | | | | | | | | | | | | |
| 2 *Metarhizium_caribense* INISAV LBM-41 | 100 | | | | | | | | | | | | | | | | | | |
| 3 *Metarhizium_caribense* INISAV LBM-42 | 99.9 | 99.9 | | | | | | | | | | | | | | | | | |
| 4 *Metarhizium_caribense* ARSEF 5197 | 100 | 100 | 99.9 | | | | | | | | | | | | | | | | |
| 5 *Metarhizium_caribense* ARSEF 1448 | 99.5 | 99.5 | 99.4 | 99.5 | | | | | | | | | | | | | | | |
| 6 *Metarhizium_pinghaense*_TYPE_CBS_257.90 | 96.2 | 96.2 | 96.1 | 96.2 | 96.1 | | | | | | | | | | | | | | |
| 7 *Metarhizium_pinghaense*_ARSEF_2231 | 96.4 | 96.4 | 96.3 | 96.4 | 96.3 | 99.0 | | | | | | | | | | | | | |
| 8 *Metarhizium_pinghaense*_ARSEF_2809 | 96.1 | 96.1 | 96.1 | 96.1 | 96.1 | 98.5 | 98.9 | | | | | | | | | | | | |
| 9 *Metarhizium_pinghaense*_ARSEF_3210 | 96.4 | 96.4 | 96.4 | 96.4 | 96.4 | 99.1 | 99.9 | 98.9 | | | | | | | | | | | |
| 10 *Metarhizium_pinghaense*_ARSEF_7929 | 96.3 | 96.3 | 96.2 | 96.3 | 96.2 | 99.1 | 99.8 | 99.0 | 99.9 | | | | | | | | | | |
| 11 *Metarhizium_pinghaense*_ARSEF_9975 | 96.2 | 96.2 | 96.2 | 96.2 | 96.2 | 99.1 | 99.6 | 98.9 | 99.7 | 99.8 | | | | | | | | | |
| 12 *Metarhizium_pinghaense*_ARSEF_4342 | 96.2 | 96.2 | 96.1 | 96.2 | 96.1 | 97.8 | 97.9 | 97.6 | 98.0 | 97.8 | 97.8 | | | | | | | | |
| 13 *Metarhizium_pinghaense*_ARSEF_4290 | 96.4 | 96.4 | 96.4 | 96.4 | 96.4 | 98.0 | 98.1 | 97.8 | 98.2 | 98.1 | 98.0 | 99.8 | | | | | | | |
| 14 *Metarhizium_pinghaense*_ARSEF_3180 | 96.4 | 96.4 | 96.4 | 96.4 | 96.4 | 98.0 | 98.1 | 97.8 | 98.2 | 98.1 | 98.0 | 99.6 | 99.8 | | | | | | |
| 15 *Metarhizium_pinghaense*_ARSEF_3604 | 96.4 | 96.4 | 96.3 | 96.4 | 96.5 | 98.0 | 98.1 | 97.7 | 98.2 | 98.0 | 97.9 | 98.5 | 98.8 | 98.8 | | | | | |
| 16 *Metarhizium_pinghaense*_ARSEF_7422 | 96.5 | 96.5 | 96.5 | 96.5 | 96.6 | 98.2 | 98.2 | 97.9 | 98.3 | 98.2 | 98.1 | 98.4 | 98.7 | 98.7 | 98.7 | | | | |
| 17 *Metarhizium_anisopliae*_TYPE_ARSEF_7487 | 93.8 | 93.8 | 93.8 | 93.8 | 93.8 | 96.2 | 95.9 | 95.6 | 95.9 | 95.9 | 95.8 | 96.0 | 96.3 | 96.3 | 95.8 | 95.9 | | | |
| 18 *Metarhizium_humberi*_ESALQ_1638 | 93.3 | 93.3 | 93.3 | 93.3 | 93.3 | 96.0 | 95.7 | 95.5 | 95.8 | 95.8 | 95.7 | 95.9 | 96.1 | 96.1 | 95.5 | 95.4 | 97.2 | | |
| 19 *Metarhizium_robertsii*_TYPE_ARSEF_2575 | 94.4 | 94.4 | 94.4 | 94.4 | 94.5 | 96.9 | 96.5 | 96.2 | 96.7 | 96.6 | 96.3 | 96.6 | 96.7 | 96.7 | 96.5 | 96.6 | 96.8 | 96.4 | |
| 20 *Metarhizium_brunneum*_TYPE_ARSEF_2107 | 92.4 | 92.4 | 92.3 | 92.4 | 92.4 | 94.9 | 94.6 | 94.4 | 94.7 | 94.6 | 94.5 | 94.6 | 94.8 | 94.8 | 94.3 | 94.3 | 94.3 | 94.3 | 94.9 |
